# Supplementary figures and images for: Phylogenomic Analyses of Snodgrassella Isolates from Honeybees and Bumblebees Reveal Taxonomic and Functional Diversity
Source: mSystems. 2022 May 23;7(3):e01500-21. doi: 10.1128/msystems.01500-21 (PMC9239279; doi:10.1128/msystems.01500-21)

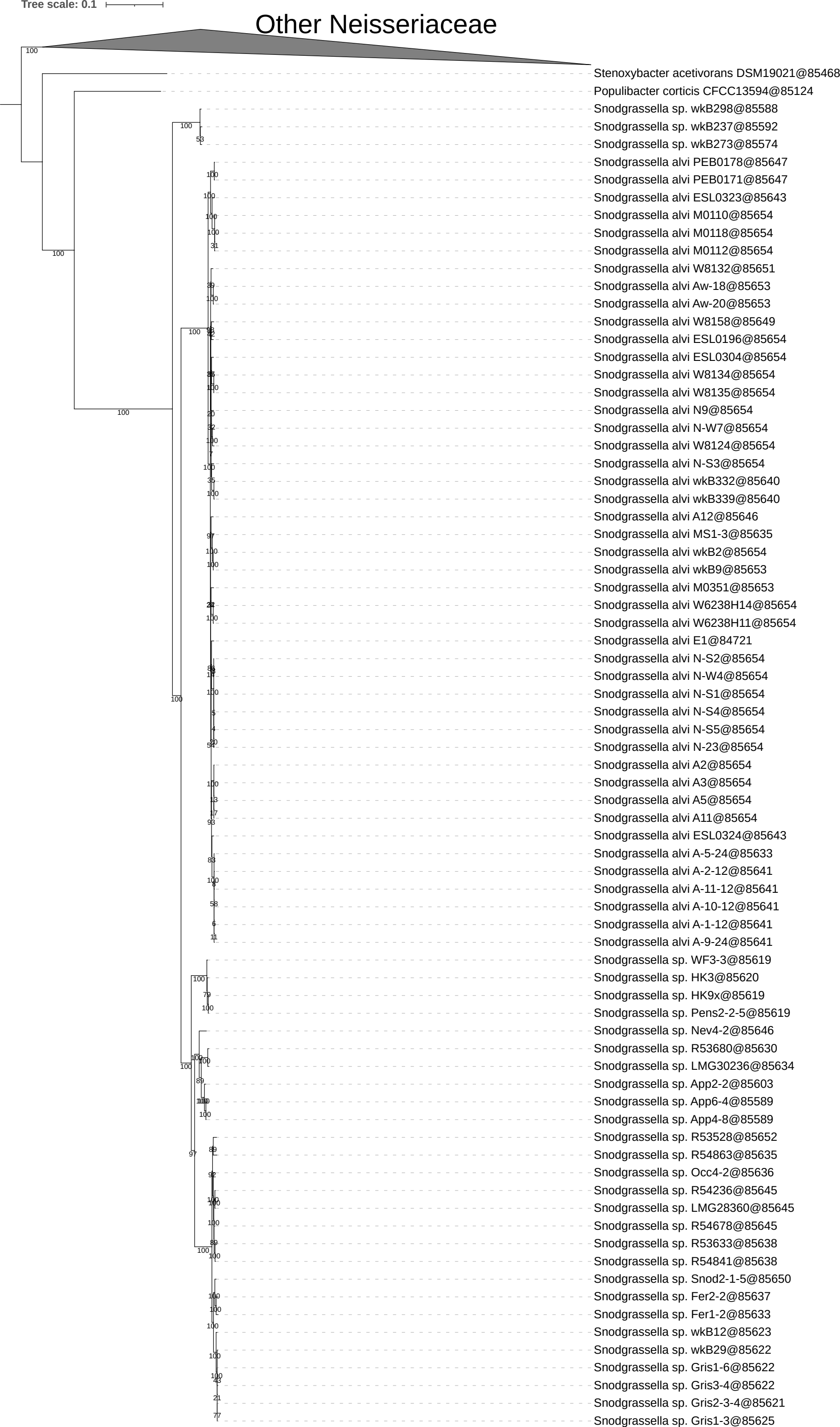

Supplement: FIG S1 [file msystems.01500-21-s0001.pdf]

Tree scale: 0.1

Tree scale: 100

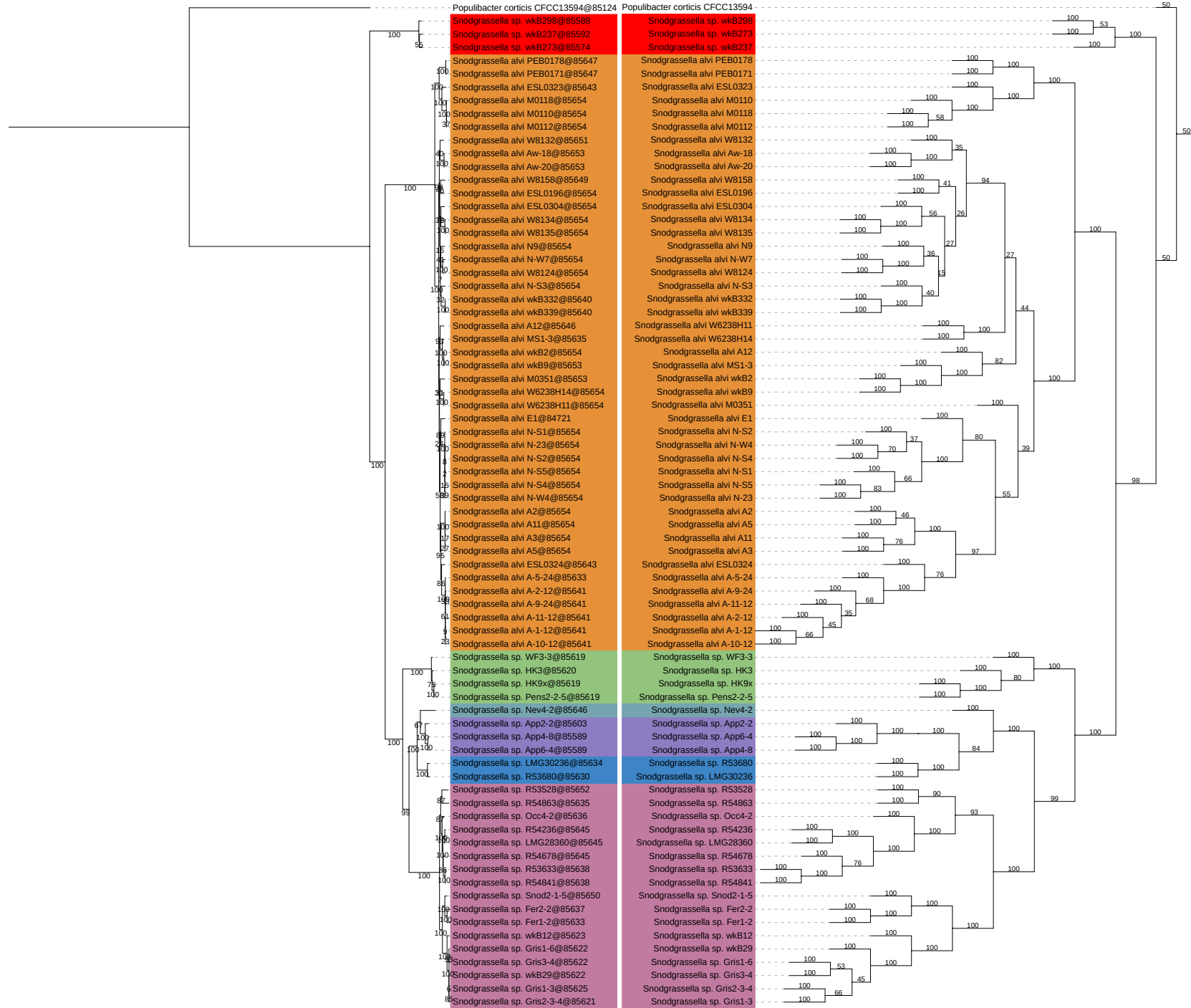

Supplement: FIG S2 [file msystems.01500-21-s0002.pdf]

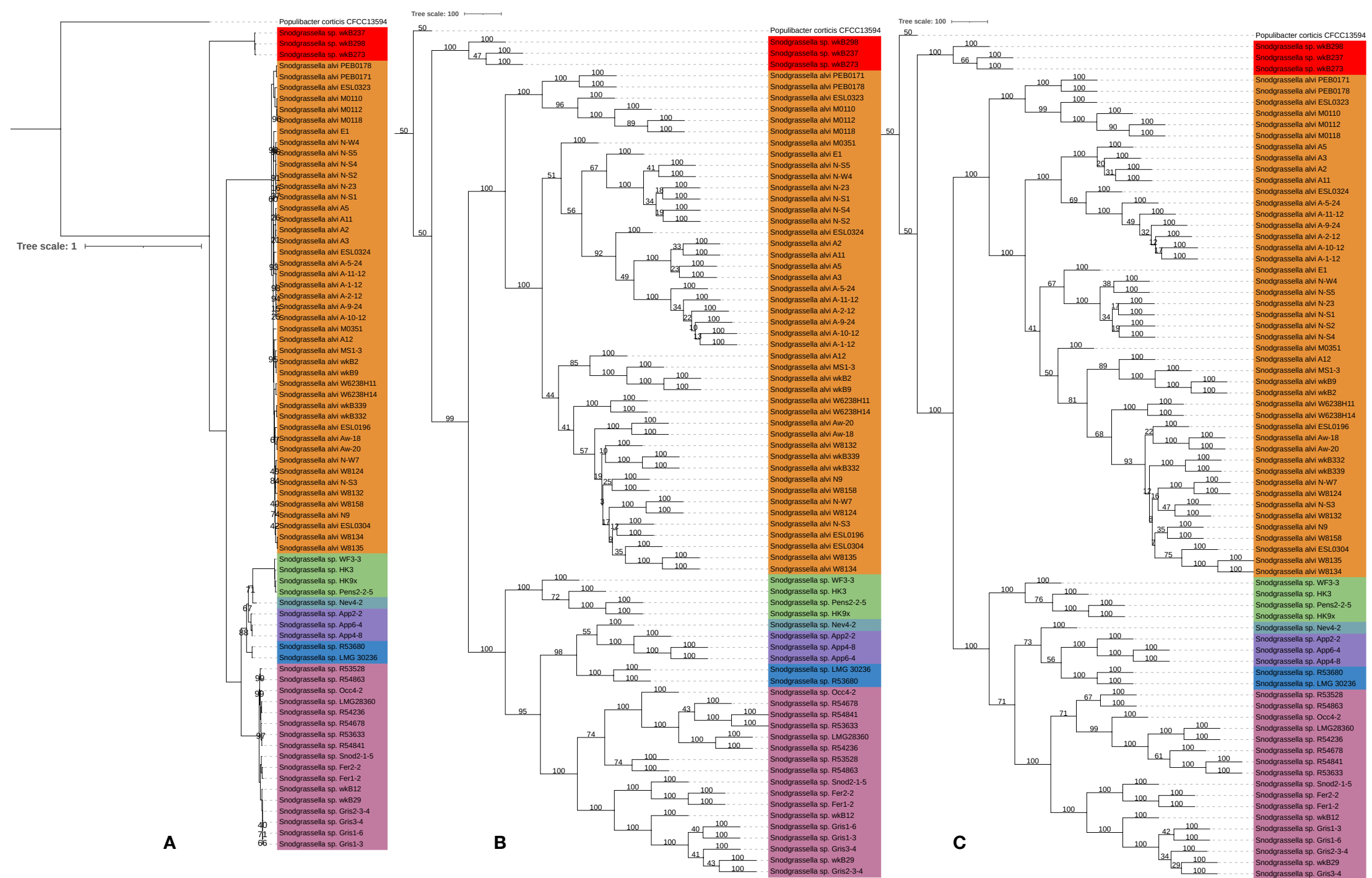

Supplement: FIG S3 [file msystems.01500-21-s0003.pdf]

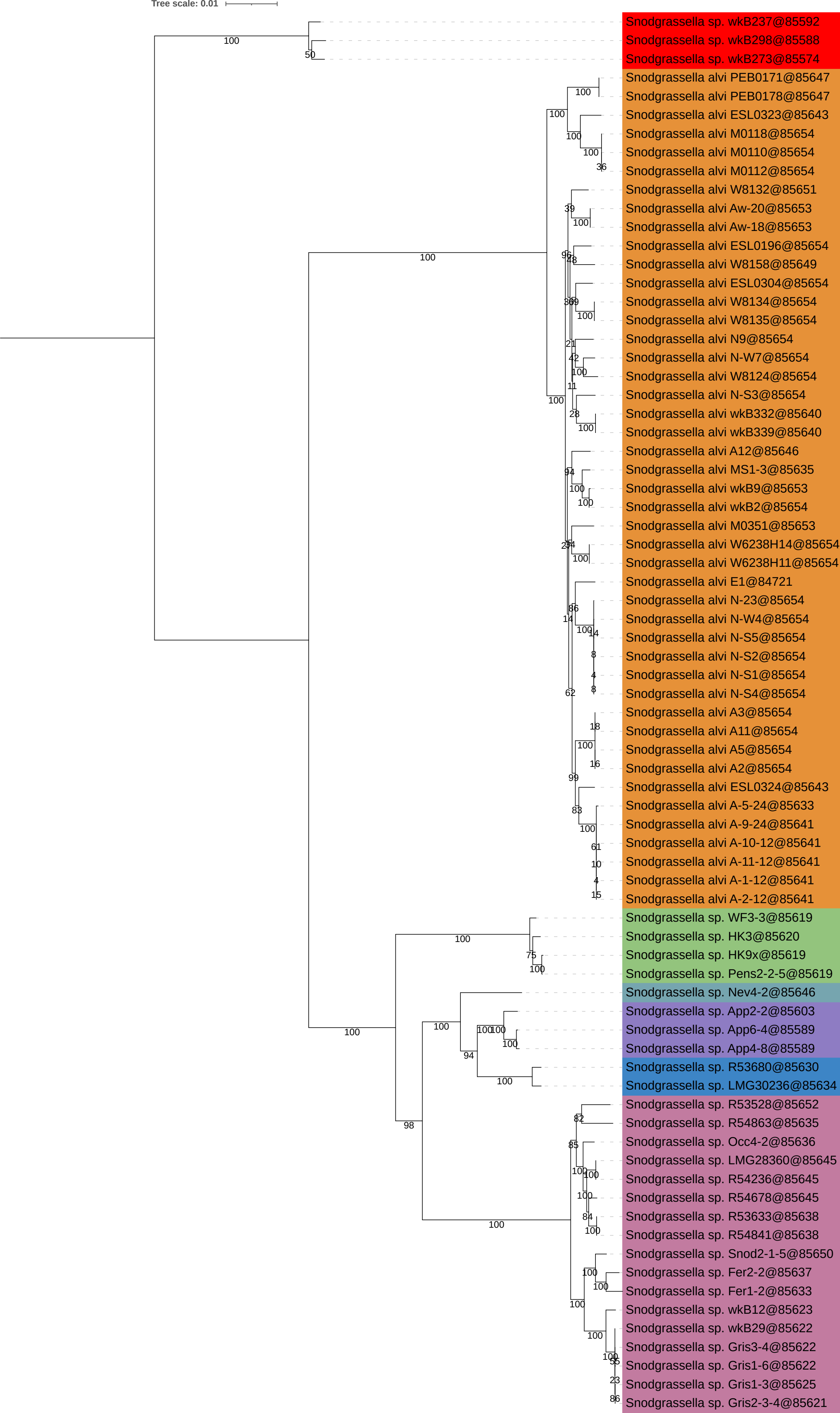

Supplement: FIG S4 [file msystems.01500-21-s0004.pdf]

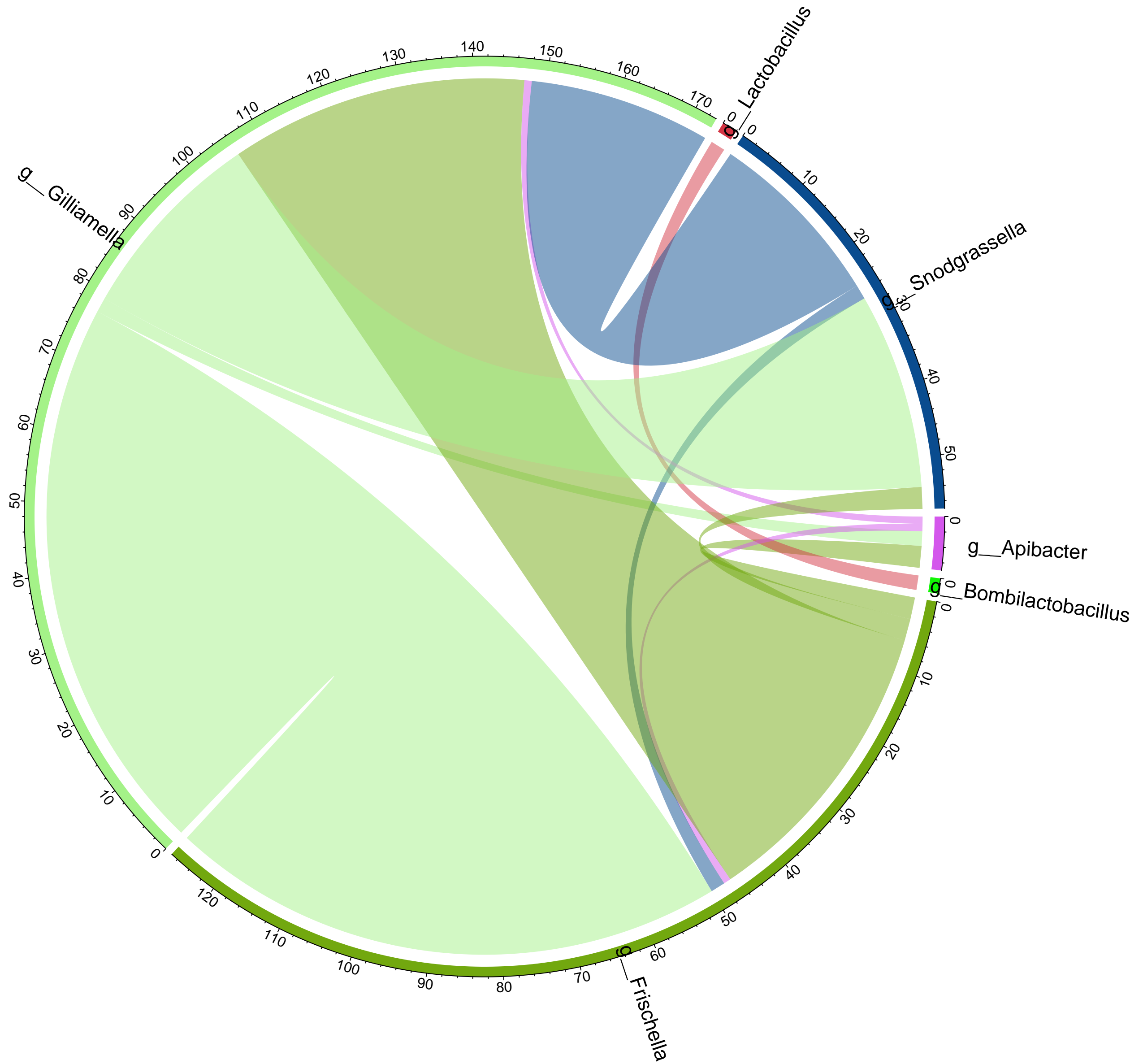

Supplement: FIG S5 [file msystems.01500-21-s0005.pdf]

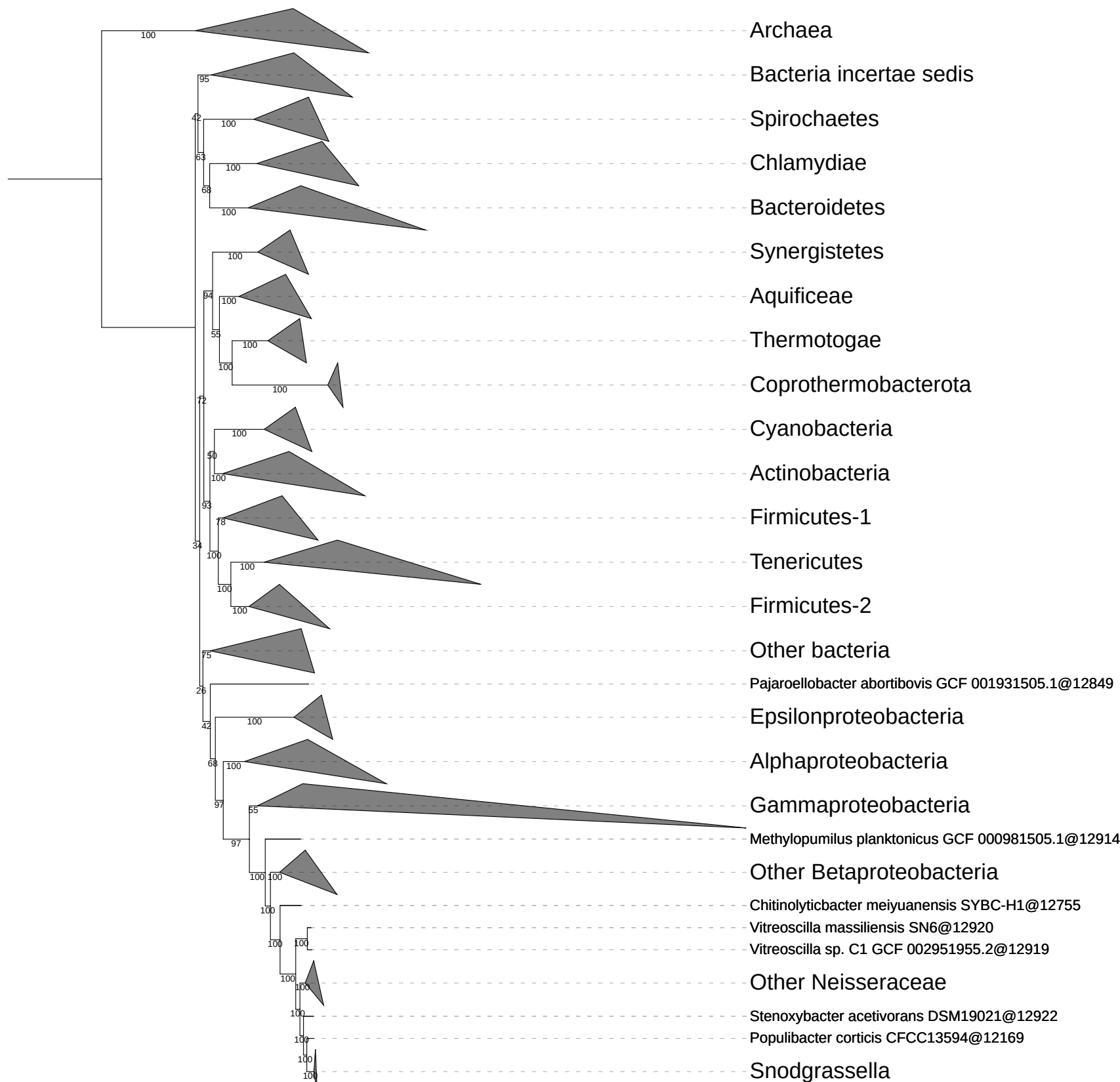

Supplement: FIG S6 [file msystems.01500-21-s0006.pdf]
