# Supplementary material for: Phylogenomic Analyses of Snodgrassella Isolates from Honeybees and Bumblebees Reveal Taxonomic and Functional Diversity
Source: mSystems. 2022 May 23;7(3):e01500-21. doi: 10.1128/msystems.01500-21 (PMC9239279; doi:10.1128/msystems.01500-21)
Supplement: TABLE S3 [file msystems.01500-21-s0010.pdf]

Recipent Gene	Recipient organim	Donor Gene	Donor organism	Identity	Mantis Function
GCF_002777655.1_00440	Snodgrassella sp._wkB273	GCF_001690235.1_00036	Gilliamella apicola	81.839	Function unknown
GCF_013346865.1_00187	Snodgrassella alvi_ESL0323	GCF_002142015.1_01688	Gilliamella sp.	97.362	Function unknown
GCF_013346865.1_00219	Snodgrassella alvi_ESL0323	GCF_007559165.1_00012	Gilliamella apicola	82.178	Function unknown
GCF_013346865.1_01261	Snodgrassella alvi_ESL0323	GCF_007559165.1_02380	Gilliamella apicola	96.247	Function unknown
GCF_013346865.1_01329	Snodgrassella alvi_ESL0323	GCF_007559165.1_00021	Gilliamella apicola	93.146	Function unknown
GCF_013346865.1_01329	Snodgrassella alvi_ESL0323	GCF_016101145.1_00402	Gilliamella sp.	93.146	Function unknown
GCA_914768015_01047	Snodgrassella sp._R54863	GCF_013346885.1_00120	Gilliamella sp.	98.105	hydrolase activity
GCF_002777825.1_02376	Snodgrassella sp._Nev4-2	GCF_001690435.1_01367	Gilliamella apicola	98.942	iron complex transport system permease protein
GCF_002777705.1_00456	Snodgrassella sp._HK3	GCF_001690825.1_00328	Gilliamella apicola	100.0	MFS transporter, DHA2 family, methylenomycin A resistance protein
GCF_002777705.1_01273	Snodgrassella sp._HK3	GCF_014489845.1_01897	Frischella japonica	76.329	Function unknown
GCF_002088675.1_01472	Snodgrassella alvi_A12	GCF_000599985.1_00868	Gilliamella apicola	99.669	Function unknown
GCF_000695565.1_00909	Snodgrassella sp._wkB12	GCF_000733115.1_01555	Gilliamella apicola	97.598	type I restriction-modification system endonuclease
GCA_914768095_02076	Snodgrassella sp._R53680	GCF_001690235.1_01649	Gilliamella apicola	82.52	Catalyzes the formation of dTDP-glucose, from dTTP and glucose 1-phosphate, as well as its pyrophosphorolysis
GCF_002777415.1_01301	Snodgrassella sp._Fer1-2	GCF_001690435.1_00001	Gilliamella apicola	86.637	FTH domain
GCF_002777415.1_01301	Snodgrassella sp._Fer1-2	GCF_001690435.1_00005	Gilliamella apicola	88.106	FTH domain
GCF_002777705.1_02401	Snodgrassella sp._HK3	GCF_001690515.1_01838	Gilliamella apicola	95.517	Function unknown
GCF_002777425.1_02070	Snodgrassella sp._App4-8	GCF_001690525.1_02016	Gilliamella apicola	91.033	Function unknown
GCF_002777325.1_00264	Snodgrassella sp._Pens2-2-5	GCF_001690535.1_01280	Gilliamella apicola	98.0	Function unknown
GCF_002777335.1_00579	Snodgrassella sp._Gris2-3-4	GCF_001690595.1_01096	Gilliamella apicola	97.382	Function unknown
GCF_002777335.1_01970	Snodgrassella sp._Gris2-3-4	GCF_001690595.1_01483	Gilliamella apicola	98.051	type I restriction-modification system endonuclease
GCF_002777425.1_01126	Snodgrassella sp._App4-8	GCF_001690605.1_00484	Gilliamella apicola	100.0	Function unknown
GCF_002777875.1_02191	Snodgrassella alvi_PEB0178	GCF_001690735.1_00403	Gilliamella apicola	94.895	Function unknown
GCF_002088675.1_01472	Snodgrassella alvi_A12	GCF_001690835.1_01215	Gilliamella apicola	100.0	Function unknown
GCF_000695565.1_00907	Snodgrassella sp._wkB12	GCF_001690875.1_00449	Gilliamella apicola	97.481	Type II restriction enzyme, methylase subunits
GCF_000695565.1_02166	Snodgrassella sp._wkB12	GCF_001690875.1_01109	Gilliamella apicola	99.596	Function unknown
GCF_002088525.1_01372	Snodgrassella alvi_N-W4	GCF_002142135.1_01470	Gilliamella apis	97.611	Function unknown
GCF_002777865.1_02181	Snodgrassella alvi_PEB0171	GCF_002142265.1_02366	Gilliamella apicola	91.09	Function unknown

GCF_013344955.1_00253	Snodgrassella alvi_ESL0324	GCF_003202705.1_01402	Frischella perrara	97.138	Function unknown
GCF_002088575.1_02062	Snodgrassella alvi_N9	GCF_003202705.1_01812	Frischella perrara	98.916	Function unknown
GCF_002088525.1_01404	Snodgrassella alvi_N-W4	GCF_003202915.1_02192	Gilliamella apicola	99.7	Function unknown
GCF_001690185.1_02392	Gilliamella apicola	GCF_002777775.1_01925	Snodgrassella sp._wkB237	86.813	Function unknown
GCF_001690175.1_02040	Gilliamella apis	GCF_002088735.1_01019	Snodgrassella alvi_A-1-12	100.0	TetR/AcrR family transcriptional regulator, tetracycline repressor protein
GCF_001693435.1_00071	Gilliamella apicola	GCF_013346865.1_02434	Snodgrassella alvi_ESL0323	97.639	Immunity protein 52
GCF_003202705.1_00934	Frischella perrara	GCF_016101535.1_01592	Snodgrassella alvi_M0118	99.656	Function unknown
GCF_900103255.1_00006	Gilliamella bombi	GCF_002777315.1_01293	Snodgrassella sp._App2-2	96.367	Function unknown
GCF_002141515.1_00438	Gilliamella apicola	GCF_002777315.1_01799	Snodgrassella sp._App2-2	96.667	Prokaryotic Cytochrome C oxidase subunit IV
GCF_001690515.1_01436	Gilliamella apicola	GCF_002777465.1_00385	Snodgrassella sp._App6-4	100.0	Function unknown
GCF_001690675.1_01788	Gilliamella apicola	GCF_002777465.1_00431	Snodgrassella sp._App6-4	100.0	Function unknown
GCF_014489845.1_01394	Frischella japonica	GCF_002777415.1_01776	Snodgrassella sp._Fer1-2	89.358	putative RNA 2'-phosphotransferase
GCF_013345045.1_01189	Gilliamella sp.	GCA_914768055_01086	Snodgrassella sp._R53528	84.387	CdiA C-terminal tRNase domain
GCF_001690675.1_00681	Gilliamella apicola	GCF_002777345.1_00217	Snodgrassella sp._Snod2-1-5	99.819	EVE domain
GCF_009795865.1_01153	Gilliamella sp.	GCF_002777825.1_00671	Snodgrassella sp._Nev4-2	93.601	Function unknown
GCF_900103085.1_01468	Gilliamella mensalis	GCA_914768045_01114	Snodgrassella sp._LMG_28360	100.0	Function unknown
GCF_000733115.1_01556	Gilliamella apicola	GCF_000695545.1_00581	Snodgrassella sp._wkB29	99.366	EVE domain
GCF_001690445.1_00463	Gilliamella apicola	GCF_016100865.1_00329	Snodgrassella alvi_W8132	88.207	MFS transporter, PAT family, beta-lactamase induction signal transducer AmpG
GCF_001690535.1_00001	Gilliamella apicola	GCA_914768055_01087	Snodgrassella sp._R53528	95.954	Function unknown
GCF_001690535.1_00196	Gilliamella apicola	GCF_002777345.1_00214	Snodgrassella sp._Snod2-1-5	96.744	Type II restriction enzyme, methylase subunits
GCF_001690535.1_00198	Gilliamella apicola	GCA_914768055_00181	Snodgrassella sp._R53528	93.768	type I restriction-modification system endonuclease
GCF_001690535.1_00581	Gilliamella apicola	GCF_002777345.1_02050	Snodgrassella sp._Snod2-1-5	95.607	Function unknown
GCF_001690535.1_00793	Gilliamella apicola	GCF_000695565.1_01489	Snodgrassella sp._wkB12	92.198	Function unknown
GCF_001690895.1_00952	Gilliamella apicola	GCF_002777745.1_00940	Snodgrassella sp._WF3-3	95.175	Transcriptional regulator
GCF_002142265.1_02036	Gilliamella apicola	GCF_013346865.1_01319	Snodgrassella alvi_ESL0323	89.158	Function unknown
GCF_002142265.1_02505	Gilliamella apicola	GCF_013346865.1_01296	Snodgrassella alvi_ESL0323	99.749	Function unknown
GCF_009795865.1_00918	Gilliamella sp.	GCA_914768035_00848	Snodgrassella sp._R54678	98.63	Function unknown
GCF_016101635.1_00535	Gilliamella sp.	GCF_013346865.1_01342	Snodgrassella alvi_ESL0323	89.362	Function unknown
GCF_900094935.1_02221	Gilliamella intestini	GCA_914768055_00124	Snodgrassella sp._R53528	98.233	Function unknown

GCF_900103085.1_00496	Gilliamella mensalis	GCA_914768025_01715	Snodgrassella sp._LMG_30236	88.752	2,5-didehydrogluconate reductase DkgB
