# Supplementary material for: Phylogenomic Analyses of Snodgrassella Isolates from Honeybees and Bumblebees Reveal Taxonomic and Functional Diversity
Source: mSystems. 2022 May 23;7(3):e01500-21. doi: 10.1128/msystems.01500-21 (PMC9239279; doi:10.1128/msystems.01500-21)
Supplement: TABLE S1 [file msystems.01500-21-s0008.pdf]

Genomes	Species	Completeness	Contamination	Length	#Contigs	N50	FTP links
GCF_000428785.1	Conchiformibius kuhniae	99.89	0.34	2118625	50	108101	ftp://ftp.ncbi.nlm.nih.gov/genomes/all/GCF/000/428/785/GCF_000428785.1_ASM42878v1
GCF_000745895.1	Stenoxybacter acetivorans	100.00	0.00	2609584	143	33302	ftp://ftp.ncbi.nlm.nih.gov/genomes/all/GCF/000/745/895/GCF_000745895.1_ASM74589v1
GCF_001027865.1	Neisseria arctica	100.00	1.77	2397977	275	112539	ftp://ftp.ncbi.nlm.nih.gov/genomes/all/GCF/001/027/865/GCF_001027865.1_ASM102786v1
GCF_001457815.1	Vitreoscilla massiliensis	99.57	0.67	3716774	10	651995	ftp://ftp.ncbi.nlm.nih.gov/genomes/all/GCF/001/457/815/GCF_001457815.1_Vitreoscilla_massiliensis
GCF_001590725.1	Populibacter corticis	98.72	0.00	2372914	131	95649	ftp://ftp.ncbi.nlm.nih.gov/genomes/all/GCF/001/590/725/GCF_001590725.1_ASM159072v1
GCF_001592185.1	Bergeriella denitrificans	99.46	0.00	2323905	393	9447	ftp://ftp.ncbi.nlm.nih.gov/genomes/all/GCF/001/592/185/GCF_001592185.1_ASM159218v1
GCF_001812885.1	Neisseria sp.	99.70	0.00	2302204	55	366058	ftp://ftp.ncbi.nlm.nih.gov/genomes/all/GCF/001/812/885/GCF_001812885.1_ASM181288v1
GCF_002073715.2	Neisseria mucosa	99.70	0.34	2783943	1	2783943	ftp://ftp.ncbi.nlm.nih.gov/genomes/all/GCF/002/073/715/GCF_002073715.2_ASM207371v2
GCF_002216145.1	Neisseria chenwenguii	99.40	0.79	2496444	1	2496444	ftp://ftp.ncbi.nlm.nih.gov/genomes/all/GCF/002/216/145/GCF_002216145.1_ASM221614v1
GCF_002237445.1	Neisseria sp.	99.20	0.00	2371912	1	2371912	ftp://ftp.ncbi.nlm.nih.gov/genomes/all/GCF/002/237/445/GCF_002237445.1_ASM223744v1
GCF_002951835.1	Simonsiella muelleri	97.69	0.53	2469862	1	2469862	ftp://ftp.ncbi.nlm.nih.gov/genomes/all/GCF/002/951/835/GCF_002951835.1_ASM295183v1
GCF_002951955.2	Vitreoscilla sp.	99.57	0.00	2612671	1	2612671	ftp://ftp.ncbi.nlm.nih.gov/genomes/all/GCF/002/951/955/GCF_002951955.2_ASM295195v2
GCF_003013245.1	Neisseria iguanae	98.12	0.49	2610244	200	22754	ftp://ftp.ncbi.nlm.nih.gov/genomes/all/GCF/003/013/245/GCF_003013245.1_ASM301324v1
GCF_003351545.1	Neisseria elongata	99.90	0.23	2397276	1	2397276	ftp://ftp.ncbi.nlm.nih.gov/genomes/all/GCF/003/351/545/GCF_003351545.1_ASM335154v1
GCF_003351665.1	Neisseria subflava	99.94	0.23	2321871	1	2321871	ftp://ftp.ncbi.nlm.nih.gov/genomes/all/GCF/003/351/665/GCF_003351665.1_ASM335166v1
GCF_003351685.1	Neisseria elongata	99.89	0.00	2534634	1	2534634	ftp://ftp.ncbi.nlm.nih.gov/genomes/all/GCF/003/351/685/GCF_003351685.1_ASM335168v1
GCF_003990355.1	Eikenella corrodens	96.73	0.23	2458478	1	2458478	ftp://ftp.ncbi.nlm.nih.gov/genomes/all/GCF/003/990/355/GCF_003990355.1_ASM399035v1
GCF_008033135.1	Chitinolyticbacter meiyuanensis	99.57	0.00	4325960	1	4325960	ftp://ftp.ncbi.nlm.nih.gov/genomes/all/GCF/008/033/135/GCF_008033135.1_ASM803313v1
GCF_008806995.1	Neisseria animalis	100.00	0.53	2236930	1	2236930	ftp://ftp.ncbi.nlm.nih.gov/genomes/all/GCF/008/806/995/GCF_008806995.1_ASM880699v1
GCF_008807015.1	Neisseria zalophi	100.00	0.00	2423693	1	2423693	ftp://ftp.ncbi.nlm.nih.gov/genomes/all/GCF/008/807/015/GCF_008807015.1_ASM880701v1
GCF_009671065.1	Neisseria brasiliensis	99.25	0.45	2617510	1	2617510	ftp://ftp.ncbi.nlm.nih.gov/genomes/all/GCF/009/671/065/GCF_009671065.1_ASM967106v1
GCF_014054725.1	Conchiformibius steedae	99.55	0.23	2205122	3	2099460	ftp://ftp.ncbi.nlm.nih.gov/genomes/all/GCF/014/054/725/GCF_014054725.1_ASM1405472v1
GCF_014054885.1	Neisseria wadsworthii	100.00	0.00	2501534	1	2501534	ftp://ftp.ncbi.nlm.nih.gov/genomes/all/GCF/014/054/885/GCF_014054885.1_ASM1405488v1

GCF_014054965.1	Neisseria shayeganii	99.81	0.32	2419744	1	2419744	ftp://ftp.ncbi.nlm.nih.gov/genomes/all/GCF/014/054/965/GCF_014054965.1_ASM1405496v1
GCF_014054985.1	Kingella oralis	97.92	0.23	2420532	2	2404499	ftp://ftp.ncbi.nlm.nih.gov/genomes/all/GCF/014/054/985/GCF_014054985.1_ASM1405498v1
GCF_014055005.1	Neisseria dentiae	99.97	0.00	2755930	1	2755930	ftp://ftp.ncbi.nlm.nih.gov/genomes/all/GCF/014/055/005/GCF_014055005.1_ASM1405500v1
GCF_016028715.1	Neisseria cinerea	98.12	0.00	1832901	1	1832901	ftp://ftp.ncbi.nlm.nih.gov/genomes/all/GCF/016/028/715/GCF_016028715.1_ASM1602871v1
GCF_016127355.1	Kingella denitrificans	98.37	0.00	2220058	1	2220058	ftp://ftp.ncbi.nlm.nih.gov/genomes/all/GCF/016/127/355/GCF_016127355.1_ASM1612735v1
GCF_900086555.1	Neisseria weaveri	100.00	0.43	2188497	1	2188497	ftp://ftp.ncbi.nlm.nih.gov/genomes/all/GCF/900/086/555/GCF_900086555.1_34903_D01
GCF_900187305.1	Neisseria zoodegmatis	100.00	0.00	2552522	1	2552522	ftp://ftp.ncbi.nlm.nih.gov/genomes/all/GCF/900/187/305/GCF_900187305.1_51470_F02
GCF_900451365.1	Kingella denitrificans	99.50	0.68	2243142	2	2235023	ftp://ftp.ncbi.nlm.nih.gov/genomes/all/GCF/900/451/365/GCF_900451365.1_50569_C01
GCF_900475315.1	Neisseria cinerea	98.06	0.00	1832904	1	1832904	ftp://ftp.ncbi.nlm.nih.gov/genomes/all/GCF/900/475/315/GCF_900475315.1_43721_G02
GCF_900475905.1	Kingella kingae	98.87	0.23	2006553	1	2006553	ftp://ftp.ncbi.nlm.nih.gov/genomes/all/GCF/900/475/905/GCF_900475905.1_49595_E01
GCF_900637855.1	Neisseria animaloris	100.00	0.00	2338721	1	2338721	ftp://ftp.ncbi.nlm.nih.gov/genomes/all/GCF/900/637/855/GCF_900637855.1_52683_F03
GCF_900638345.1	Neisseria animaloris	100.00	0.00	2283939	1	2283939	ftp://ftp.ncbi.nlm.nih.gov/genomes/all/GCF/900/638/345/GCF_900638345.1_57428_C01
