# Supplementary material for: Phylogenomic Analyses of Snodgrassella Isolates from Honeybees and Bumblebees Reveal Taxonomic and Functional Diversity
Source: mSystems. 2022 May 23;7(3):e01500-21. doi: 10.1128/msystems.01500-21 (PMC9239279; doi:10.1128/msystems.01500-21)
Supplement: TABLE S2 [file msystems.01500-21-s0009.pdf]

Genome	Group	Species	Completeness	Contamination	Ftp links
GCF_001418685.1	Apibacter	Apibacter mensalis	100.00	0.00	ftp://ftp.ncbi.nlm.nih.gov/genomes/all/GCF/001/418/685/GCF_001418685.1_ASM141868v1
GCF_002964915.1	Apibacter	Apibacter sp.	100.00	0.00	ftp://ftp.ncbi.nlm.nih.gov/genomes/all/GCF/002/964/915/GCF_002964915.1_ASM296491v1
GCF_002964965.1	Apibacter	Apibacter adventoris	100.00	0.00	ftp://ftp.ncbi.nlm.nih.gov/genomes/all/GCF/002/964/965/GCF_002964965.1_ASM296496v1
GCF_002964975.1	Apibacter	Apibacter adventoris	100.00	1.08	ftp://ftp.ncbi.nlm.nih.gov/genomes/all/GCF/002/964/975/GCF_002964975.1_ASM296497v1
GCF_004014855.1	Apibacter	Apibacter raozihei	100.00	0.00	ftp://ftp.ncbi.nlm.nih.gov/genomes/all/GCF/004/014/855/GCF_004014855.1_ASM401485v1
GCF_004023655.1	Apibacter	Apibacter sp.	100.00	0.00	ftp://ftp.ncbi.nlm.nih.gov/genomes/all/GCF/004/023/655/GCF_004023655.1_ASM402365v1
GCF_007845585.1	Apibacter	Apibacter muscae	100.00	0.00	ftp://ftp.ncbi.nlm.nih.gov/genomes/all/GCF/007/845/585/GCF_007845585.1_ASM784558v1
GCF_007845595.1	Apibacter	Apibacter muscae	100.00	0.00	ftp://ftp.ncbi.nlm.nih.gov/genomes/all/GCF/007/845/595/GCF_007845595.1_ASM784559v1
GCF_007845635.1	Apibacter	Apibacter muscae	100.00	0.00	ftp://ftp.ncbi.nlm.nih.gov/genomes/all/GCF/007/845/635/GCF_007845635.1_ASM784563v1
GCF_009827705.1	Apibacter	Apibacter sp.	100.00	0.00	ftp://ftp.ncbi.nlm.nih.gov/genomes/all/GCF/009/827/705/GCF_009827705.1_ASM982770v1
GCF_009827735.1	Apibacter	Apibacter sp.	100.00	0.00	ftp://ftp.ncbi.nlm.nih.gov/genomes/all/GCF/009/827/735/GCF_009827735.1_ASM982773v1
GCF_009827745.1	Apibacter	Apibacter sp.	100.00	0.00	ftp://ftp.ncbi.nlm.nih.gov/genomes/all/GCF/009/827/745/GCF_009827745.1_ASM982774v1
GCF_009827755.1	Apibacter	Apibacter sp.	100.00	0.00	ftp://ftp.ncbi.nlm.nih.gov/genomes/all/GCF/009/827/755/GCF_009827755.1_ASM982775v1
GCF_009827795.1	Apibacter	Apibacter sp.	100.00	0.00	ftp://ftp.ncbi.nlm.nih.gov/genomes/all/GCF/009/827/795/GCF_009827795.1_ASM982779v1
GCF_009827805.1	Apibacter	Apibacter sp.	100.00	0.00	ftp://ftp.ncbi.nlm.nih.gov/genomes/all/GCF/009/827/805/GCF_009827805.1_ASM982780v1
GCF_009827835.1	Apibacter	Apibacter sp.	100.00	0.00	ftp://ftp.ncbi.nlm.nih.gov/genomes/all/GCF/009/827/835/GCF_009827835.1_ASM982783v1
GCF_009827845.1	Apibacter	Apibacter sp.	100.00	0.00	ftp://ftp.ncbi.nlm.nih.gov/genomes/all/GCF/009/827/845/GCF_009827845.1_ASM982784v1
GCF_009827855.1	Apibacter	Apibacter sp.	100.00	0.00	ftp://ftp.ncbi.nlm.nih.gov/genomes/all/GCF/009/827/855/GCF_009827855.1_ASM982785v1
GCF_009827885.1	Apibacter	Apibacter sp.	100.00	0.00	ftp://ftp.ncbi.nlm.nih.gov/genomes/all/GCF/009/827/885/GCF_009827885.1_ASM982788v1
GCF_009828155.1	Apibacter	Apibacter sp.	100.00	0.00	ftp://ftp.ncbi.nlm.nih.gov/genomes/all/GCF/009/828/155/GCF_009828155.1_ASM982815v1
GCF_009828165.1	Apibacter	Apibacter sp.	100.00	0.00	ftp://ftp.ncbi.nlm.nih.gov/genomes/all/GCF/009/828/165/GCF_009828165.1_ASM982816v1
GCF_011082485.1	Apibacter	Apibacter sp.	100.00	0.00	ftp://ftp.ncbi.nlm.nih.gov/genomes/all/GCF/011/082/485/GCF_011082485.1_ASM1108248v1
GCF_011082725.1	Apibacter	Apibacter sp.	100.00	0.00	ftp://ftp.ncbi.nlm.nih.gov/genomes/all/GCF/011/082/725/GCF_011082725.1_ASM1108272v1
GCF_900018365.1	Apibacter	Apibacter mensalis	100.00	0.00	ftp://ftp.ncbi.nlm.nih.gov/genomes/all/GCF/900/018/365/GCF_900018365.1_IMG-taxon_2615840617_annotated_assembly
GCF_000022725.1	Bartonella	Bartonella grahamii	99.89	0.44	ftp://ftp.ncbi.nlm.nih.gov/genomes/all/GCF/000/022/725/GCF_000022725.1_ASM2272v1
GCF_000046685.1	Bartonella	Bartonella quintana	99.18	0.00	ftp://ftp.ncbi.nlm.nih.gov/genomes/all/GCF/000/046/685/GCF_000046685.1_ASM4668v1
GCF_000046705.1	Bartonella	Bartonella henselae	100.00	0.00	ftp://ftp.ncbi.nlm.nih.gov/genomes/all/GCF/000/046/705/GCF_000046705.1_ASM4670v1
GCF_000196435.1	Bartonella	Bartonella tribocorum	100.00	1.06	ftp://ftp.ncbi.nlm.nih.gov/genomes/all/GCF/000/196/435/GCF_000196435.1_ASM19643v1
GCF_000253015.1	Bartonella	Bartonella clarridgeiae	100.00	0.42	ftp://ftp.ncbi.nlm.nih.gov/genomes/all/GCF/000/253/015/GCF_000253015.1_ASM25301v1
GCF_000273375.1	Bartonella	Bartonella birtlesii	99.45	0.11	ftp://ftp.ncbi.nlm.nih.gov/genomes/all/GCF/000/273/375/GCF_000273375.1_ASM27337v1
GCF_000278095.1	Bartonella	Bartonella birtlesii	99.89	0.11	ftp://ftp.ncbi.nlm.nih.gov/genomes/all/GCF/000/278/095/GCF_000278095.1_Bart_birt_LL-WM9_V1
GCF_000278115.1	Bartonella	Bartonella sp.	99.34	0.00	ftp://ftp.ncbi.nlm.nih.gov/genomes/all/GCF/000/278/115/GCF_000278115.1_Bart_sp_DB5-6_V1
GCF_000278135.1	Bartonella	Bartonella washoeensis	100.00	0.00	ftp://ftp.ncbi.nlm.nih.gov/genomes/all/GCF/000/278/135/GCF_000278135.1_Bart_wash_Sb944nv_V1
GCF_000278155.1	Bartonella	Bartonella doshiae	100.00	0.00	ftp://ftp.ncbi.nlm.nih.gov/genomes/all/GCF/000/278/155/GCF_000278155.1_Bart_dosh_NCTC_12862_R18_V1
GCF_000278175.1	Bartonella	Bartonella elizabethae	99.78	0.00	ftp://ftp.ncbi.nlm.nih.gov/genomes/all/GCF/000/278/175/GCF_000278175.1_Bart_eliz_Re6043vi_V1
GCF_000278195.1	Bartonella	Bartonella washoeensis	100.00	0.00	ftp://ftp.ncbi.nlm.nih.gov/genomes/all/GCF/000/278/195/GCF_000278195.1_Bart_wash_085-0475_V1

GCF_000 278215.1	Bartonella	Bartonella rattimassilien sis	99.81	0.46	ftp://ftp.ncbi.nlm.nih.gov/genomes/all/GCF/000/278/215/GCF_000278 215.1_Bart_ratt_15908_V1
GCF_000 278235.1	Bartonella	Bartonella vinsonii	99.78	0.00	ftp://ftp.ncbi.nlm.nih.gov/genomes/all/GCF/000/278/235/GCF_000278 235.1_Bart_vins_OK-94-513_V1
GCF_000 278255.1	Bartonella	Bartonella melophagi	100.00	0.00	ftp://ftp.ncbi.nlm.nih.gov/genomes/all/GCF/000/278/255/GCF_000278 255.1_Bart_melo_K-2C_V1
GCF_000 278275.1	Bartonella	Bartonella tamiae	100.00	0.00	ftp://ftp.ncbi.nlm.nih.gov/genomes/all/GCF/000/278/275/GCF_000278 275.1_Bart_tami_Th239_V1
GCF_000 278295.1	Bartonella	Bartonella taylorii	99.56	0.00	ftp://ftp.ncbi.nlm.nih.gov/genomes/all/GCF/000/278/295/GCF_000278 295.1_Bart_tayl_8TBB_V1
GCF_000 278315.1	Bartonella	Bartonella elizabethae	99.29	0.00	ftp://ftp.ncbi.nlm.nih.gov/genomes/all/GCF/000/278/315/GCF_000278 315.1_Bart_eliz_F9251_V1
GCF_000 278335.1	Bartonella	Bartonella vinsonii	99.78	0.00	ftp://ftp.ncbi.nlm.nih.gov/genomes/all/GCF/000/278/335/GCF_000278 335.1_Bart_vins_Pm136co_V1
GCF_000 279995.1	Bartonella	Bartonella tamiae	99.56	0.00	ftp://ftp.ncbi.nlm.nih.gov/genomes/all/GCF/000/279/995/GCF_000279 995.1_Bart_tami_Th307_V1
GCF_000 280015.1	Bartonella	Bartonella alsatica	99.12	0.00	ftp://ftp.ncbi.nlm.nih.gov/genomes/all/GCF/000/280/015/GCF_000280 015.1_Bart_alsa_IBS_382_V1
GCF_000 294715.1	Bartonella	Bartonella quintana	98.69	0.00	ftp://ftp.ncbi.nlm.nih.gov/genomes/all/GCF/000/294/715/GCF_000294 715.1_ASM29471v1
GCF_000 312525.1	Bartonella	Bartonella florencae	99.79	0.20	ftp://ftp.ncbi.nlm.nih.gov/genomes/all/GCF/000/312/525/GCF_000312 525.1_ASM31252v1
GCF_000 312545.1	Bartonella	Bartonella senegalensis	98.88	0.61	ftp://ftp.ncbi.nlm.nih.gov/genomes/all/GCF/000/312/545/GCF_000312 545.1_ASM31254v1
GCF_000 312565.1	Bartonella	Bartonella rattaustralian i	98.97	0.16	ftp://ftp.ncbi.nlm.nih.gov/genomes/all/GCF/000/312/565/GCF_000312 565.1_ASM31256v2
GCF_000 312585.1	Bartonella	Bartonella queenslande nsis	94.25	0.90	ftp://ftp.ncbi.nlm.nih.gov/genomes/all/GCF/000/312/585/GCF_000312 585.1_ASM31258v1
GCF_000 312605.1	Bartonella	Bartonella rattimassilien sis	99.79	0.31	ftp://ftp.ncbi.nlm.nih.gov/genomes/all/GCF/000/312/605/GCF_000312 605.1_ASM31260v2
GCF_000 341355.1	Bartonella	Bartonella australis	98.46	0.14	ftp://ftp.ncbi.nlm.nih.gov/genomes/all/GCF/000/341/355/GCF_000341 355.1_ASM34135v1
GCF_000 341385.1	Bartonella	Bartonella vinsonii	100.00	0.00	ftp://ftp.ncbi.nlm.nih.gov/genomes/all/GCF/000/341/385/GCF_000341 385.1_ASM34138v1
GCF_000 384965.1	Bartonella	Bartonella bovis	99.38	0.41	ftp://ftp.ncbi.nlm.nih.gov/genomes/all/GCF/000/384/965/GCF_000384 965.1_BBbMcIrM01
GCF_000 385395.1	Bartonella	Bartonella bovis	99.38	0.00	ftp://ftp.ncbi.nlm.nih.gov/genomes/all/GCF/000/385/395/GCF_000385 395.1_m02McIrM01
GCF_000 385415.1	Bartonella	Bartonella vinsonii	99.96	0.09	ftp://ftp.ncbi.nlm.nih.gov/genomes/all/GCF/000/385/415/GCF_000385 415.1_BVtwMcIrM01
GCF_000 385435.1	Bartonella	Bartonella schoenbuche nsis	99.38	0.00	ftp://ftp.ncbi.nlm.nih.gov/genomes/all/GCF/000/385/435/GCF_000385 435.1_m07aNrPeIrM01
GCF_000 400145.1	Bartonella	Bartonella birtlesii	98.15	0.26	ftp://ftp.ncbi.nlm.nih.gov/genomes/all/GCF/000/400/145/GCF_000400 145.1_ASM40014v1
GCF_000 400165.1	Bartonella	Bartonella birtlesii	98.08	0.11	ftp://ftp.ncbi.nlm.nih.gov/genomes/all/GCF/000/400/165/GCF_000400 165.1_ASM40016v1
GCF_000 400185.1	Bartonella	Bartonella birtlesii	99.64	0.11	ftp://ftp.ncbi.nlm.nih.gov/genomes/all/GCF/000/400/185/GCF_000400 185.1_ASM40018v1
GCF_000 516675.1	Bartonella	Bartonella henselae	99.59	0.00	ftp://ftp.ncbi.nlm.nih.gov/genomes/all/GCF/000/516/675/GCF_000516 675.1_Bart_hens_JK_51_V1
GCF_000 516695.1	Bartonella	Bartonella henselae	99.59	0.20	ftp://ftp.ncbi.nlm.nih.gov/genomes/all/GCF/000/516/695/GCF_000516 695.1_Bart_hens_JK_50_V1
GCF_000 516715.1	Bartonella	Bartonella henselae	100.00	0.00	ftp://ftp.ncbi.nlm.nih.gov/genomes/all/GCF/000/516/715/GCF_000516 715.1_Bart_hens_JK_42_V1
GCF_000 516735.1	Bartonella	Bartonella henselae	99.59	0.00	ftp://ftp.ncbi.nlm.nih.gov/genomes/all/GCF/000/516/735/GCF_000516 735.1_Bart_hens_JK_41_V1
GCF_000 516755.1	Bartonella	Bartonella quintana	98.77	0.41	ftp://ftp.ncbi.nlm.nih.gov/genomes/all/GCF/000/516/755/GCF_000516 755.1_Bart_quin_BQ2-D70_V1
GCF_000 516775.1	Bartonella	Bartonella quintana	98.98	0.00	ftp://ftp.ncbi.nlm.nih.gov/genomes/all/GCF/000/516/775/GCF_000516 775.1_Bart_quin_JK_73rel_V1
GCF_000 516795.1	Bartonella	Bartonella quintana	98.98	0.00	ftp://ftp.ncbi.nlm.nih.gov/genomes/all/GCF/000/516/795/GCF_000516 795.1_Bart_quin_JK_73_V1
GCF_000 516815.1	Bartonella	Bartonella quintana	98.77	0.00	ftp://ftp.ncbi.nlm.nih.gov/genomes/all/GCF/000/516/815/GCF_000516 815.1_Bart_quin_JK_12_V1
GCF_000 516835.1	Bartonella	Bartonella quintana	98.98	0.00	ftp://ftp.ncbi.nlm.nih.gov/genomes/all/GCF/000/516/835/GCF_000516 835.1_Bart_quin_JK_7_V1
GCF_000 518085.1	Bartonella	Bartonella grahamii	99.89	0.00	ftp://ftp.ncbi.nlm.nih.gov/genomes/all/GCF/000/518/085/GCF_000518 085.1_ASM51808v1

GCF_000518105.1	Bartonella	Bartonella vinsonii	99.96	0.09	ftp://ftp.ncbi.nlm.nih.gov/genomes/all/GCF/000/518/105/GCF_000518105.1_ASM51810v1
GCF_000518165.1	Bartonella	Bartonella elizabethae	100.00	0.00	ftp://ftp.ncbi.nlm.nih.gov/genomes/all/GCF/000/518/165/GCF_000518165.1_ASM51816v1
GCF_000518185.1	Bartonella	Bartonella clarridgeiae	100.00	0.00	ftp://ftp.ncbi.nlm.nih.gov/genomes/all/GCF/000/518/185/GCF_000518185.1_ASM51818v1
GCF_000526895.1	Bartonella	Bartonella doshiae	100.00	0.00	ftp://ftp.ncbi.nlm.nih.gov/genomes/all/GCF/000/526/895/GCF_000526895.1_ASM52689v1
GCF_000607205.1	Bartonella	Bartonella bacilliformis	96.67	0.00	ftp://ftp.ncbi.nlm.nih.gov/genomes/all/GCF/000/607/205/GCF_000607205.1_Bart_baci_San_Pedro600-02_V1
GCF_000607225.1	Bartonella	Bartonella bacilliformis	97.18	0.21	ftp://ftp.ncbi.nlm.nih.gov/genomes/all/GCF/000/607/225/GCF_000607225.1_Bart_baci_Heidi_Mejia_V1
GCF_000607245.1	Bartonella	Bartonella bacilliformis	97.19	0.00	ftp://ftp.ncbi.nlm.nih.gov/genomes/all/GCF/000/607/245/GCF_000607245.1_Bart_baci_Peru-18_V1
GCF_000612965.1	Bartonella	Bartonella henselae	100.00	0.00	ftp://ftp.ncbi.nlm.nih.gov/genomes/all/GCF/000/612/965/GCF_000612965.1_PRJEB4971_assembly_1
GCF_000689355.1	Bartonella	Bartonella tribocorum	100.00	1.06	ftp://ftp.ncbi.nlm.nih.gov/genomes/all/GCF/000/689/355/GCF_000689355.1_PRJEB5999_assembly_1
GCF_000706625.1	Bartonella	Bartonella koehlerae	99.59	0.00	ftp://ftp.ncbi.nlm.nih.gov/genomes/all/GCF/000/706/625/GCF_000706625.1_Bart_koeh_C29_V1
GCF_000706645.1	Bartonella	Bartonella rochalimae	98.85	0.00	ftp://ftp.ncbi.nlm.nih.gov/genomes/all/GCF/000/706/645/GCF_000706645.1_Bart_roch_BMGH_V1
GCF_000708485.1	Bartonella	Bartonella henselae	99.18	0.00	ftp://ftp.ncbi.nlm.nih.gov/genomes/all/GCF/000/708/485/GCF_000708485.1_Bart_hens_Zeus_V1
GCF_000708545.1	Bartonella	Bartonella henselae	99.18	0.00	ftp://ftp.ncbi.nlm.nih.gov/genomes/all/GCF/000/708/545/GCF_000708545.1_Bart_hens_JK_53_V1
GCF_000708575.1	Bartonella	Bartonella quintana	99.18	0.00	ftp://ftp.ncbi.nlm.nih.gov/genomes/all/GCF/000/708/575/GCF_000708575.1_Bart_quin_JK_19_V1
GCF_000708815.1	Bartonella	Bartonella quintana	98.77	0.00	ftp://ftp.ncbi.nlm.nih.gov/genomes/all/GCF/000/708/815/GCF_000708815.1_Bart_quin_JK_31_V1
GCF_000708865.1	Bartonella	Bartonella quintana	98.57	0.41	ftp://ftp.ncbi.nlm.nih.gov/genomes/all/GCF/000/708/865/GCF_000708865.1_Bart_quin_JK_63_V1
GCF_000708905.1	Bartonella	Bartonella quintana	98.77	0.14	ftp://ftp.ncbi.nlm.nih.gov/genomes/all/GCF/000/708/905/GCF_000708905.1_Bart_quin_JK_56_V1
GCF_000708945.1	Bartonella	Bartonella quintana	99.18	0.00	ftp://ftp.ncbi.nlm.nih.gov/genomes/all/GCF/000/708/945/GCF_000708945.1_Bart_quin_JK_68_V1
GCF_000708965.1	Bartonella	Bartonella quintana	98.77	0.00	ftp://ftp.ncbi.nlm.nih.gov/genomes/all/GCF/000/708/965/GCF_000708965.1_Bart_quin_JK_67_V1
GCF_000708985.1	Bartonella	Bartonella quintana	98.77	0.00	ftp://ftp.ncbi.nlm.nih.gov/genomes/all/GCF/000/708/985/GCF_000708985.1_Bart_quin_JK39_V1
GCF_000709735.1	Bartonella	Bartonella bacilliformis	96.64	1.37	ftp://ftp.ncbi.nlm.nih.gov/genomes/all/GCF/000/709/735/GCF_000709735.1_Bart_baci_Hosp800-02_V1
GCF_000709755.1	Bartonella	Bartonella bacilliformis	97.19	0.00	ftp://ftp.ncbi.nlm.nih.gov/genomes/all/GCF/000/709/755/GCF_000709755.1_Bart_baci_CUSCO5_V1
GCF_000709775.1	Bartonella	Bartonella bacilliformis	96.67	0.00	ftp://ftp.ncbi.nlm.nih.gov/genomes/all/GCF/000/709/775/GCF_000709775.1_Bart_baci_Cond044_V1
GCF_000709795.1	Bartonella	Bartonella bacilliformis	97.56	0.41	ftp://ftp.ncbi.nlm.nih.gov/genomes/all/GCF/000/709/795/GCF_000709795.1_Bart_baci_Ver097_V1
GCF_000709815.1	Bartonella	Bartonella bacilliformis	97.09	0.00	ftp://ftp.ncbi.nlm.nih.gov/genomes/all/GCF/000/709/815/GCF_000709815.1_Bart_baci_Peru38_V1
GCF_000709835.1	Bartonella	Bartonella bacilliformis	97.05	0.21	ftp://ftp.ncbi.nlm.nih.gov/genomes/all/GCF/000/709/835/GCF_000709835.1_Bart_baci_VAB9028_V1
GCF_000709855.1	Bartonella	Bartonella bacilliformis	97.09	0.00	ftp://ftp.ncbi.nlm.nih.gov/genomes/all/GCF/000/709/855/GCF_000709855.1_Bart_baci_Ver075_V1
GCF_000709875.1	Bartonella	Bartonella bacilliformis	97.18	0.21	ftp://ftp.ncbi.nlm.nih.gov/genomes/all/GCF/000/709/875/GCF_000709875.1_Bart_baci_CAR600-02_V1
GCF_001281405.1	Bartonella	Bartonella ancashensis	97.12	0.08	ftp://ftp.ncbi.nlm.nih.gov/genomes/all/GCF/001/281/405/GCF_001281405.1_ASM128140v1
GCF_001291465.1	Bartonella	Bartonella henselae	100.00	0.00	ftp://ftp.ncbi.nlm.nih.gov/genomes/all/GCF/001/291/465/GCF_001291465.1_Bartonella_henselae_MVT02
GCF_001525625.2	Bartonella	Bartonella henselae	100.00	0.82	ftp://ftp.ncbi.nlm.nih.gov/genomes/all/GCF/001/525/625/GCF_001525625.2_ASM152562v2
GCF_001624625.1	Bartonella	Bartonella bacilliformis	97.09	0.21	ftp://ftp.ncbi.nlm.nih.gov/genomes/all/GCF/001/624/625/GCF_001624625.1_ASM162462v1
GCF_001625425.1	Bartonella	Bartonella bacilliformis	97.09	0.00	ftp://ftp.ncbi.nlm.nih.gov/genomes/all/GCF/001/625/425/GCF_001625425.1_ASM162542v1
GCF_001932075.1	Bartonella	Bartonella henselae	100.00	0.00	ftp://ftp.ncbi.nlm.nih.gov/genomes/all/GCF/001/932/075/GCF_001932075.1_ASM193207v1
GCF_001932085.1	Bartonella	Bartonella henselae	100.00	0.41	ftp://ftp.ncbi.nlm.nih.gov/genomes/all/GCF/001/932/085/GCF_001932085.1_ASM193208v1
GCF_001932095.1	Bartonella	Bartonella henselae	100.00	0.41	ftp://ftp.ncbi.nlm.nih.gov/genomes/all/GCF/001/932/095/GCF_001932095.1_ASM193209v1
GCF_001932135.1	Bartonella	Bartonella henselae	100.00	0.00	ftp://ftp.ncbi.nlm.nih.gov/genomes/all/GCF/001/932/135/GCF_001932135.1_ASM193213v1

GCF_001932145.1	Bartonella	Bartonella henselae	100.00	0.00	ftp://ftp.ncbi.nlm.nih.gov/genomes/all/GCF/001/932/145/GCF_001932145.1_ASM193214v1
GCF_001932165.1	Bartonella	Bartonella henselae	100.00	0.14	ftp://ftp.ncbi.nlm.nih.gov/genomes/all/GCF/001/932/165/GCF_001932165.1_ASM193216v1
GCF_001932175.1	Bartonella	Bartonella henselae	100.00	0.00	ftp://ftp.ncbi.nlm.nih.gov/genomes/all/GCF/001/932/175/GCF_001932175.1_ASM193217v1
GCF_001932215.1	Bartonella	Bartonella henselae	100.00	0.00	ftp://ftp.ncbi.nlm.nih.gov/genomes/all/GCF/001/932/215/GCF_001932215.1_ASM193221v1
GCF_001932225.1	Bartonella	Bartonella henselae	99.59	0.00	ftp://ftp.ncbi.nlm.nih.gov/genomes/all/GCF/001/932/225/GCF_001932225.1_ASM193222v1
GCF_001932235.1	Bartonella	Bartonella henselae	100.00	0.00	ftp://ftp.ncbi.nlm.nih.gov/genomes/all/GCF/001/932/235/GCF_001932235.1_ASM193223v1
GCF_001932245.1	Bartonella	Bartonella henselae	100.00	0.00	ftp://ftp.ncbi.nlm.nih.gov/genomes/all/GCF/001/932/245/GCF_001932245.1_ASM193224v1
GCF_001932295.1	Bartonella	Bartonella henselae	100.00	0.00	ftp://ftp.ncbi.nlm.nih.gov/genomes/all/GCF/001/932/295/GCF_001932295.1_ASM193229v1
GCF_001952045.1	Bartonella	Bartonella apis	100.00	0.00	ftp://ftp.ncbi.nlm.nih.gov/genomes/all/GCF/001/952/045/GCF_001952045.1_ASM195204v1
GCF_001952065.1	Bartonella	Bartonella apis	100.00	0.00	ftp://ftp.ncbi.nlm.nih.gov/genomes/all/GCF/001/952/065/GCF_001952065.1_ASM195206v1
GCF_001952075.1	Bartonella	Bartonella apis	100.00	0.00	ftp://ftp.ncbi.nlm.nih.gov/genomes/all/GCF/001/952/075/GCF_001952075.1_ASM195207v1
GCF_002007485.1	Bartonella	Bartonella apis	100.00	0.00	ftp://ftp.ncbi.nlm.nih.gov/genomes/all/GCF/002/007/485/GCF_002007485.1_ASM200748v1
GCF_002007505.1	Bartonella	Bartonella apis	100.00	0.00	ftp://ftp.ncbi.nlm.nih.gov/genomes/all/GCF/002/007/505/GCF_002007505.1_ASM200750v1
GCF_002007565.1	Bartonella	Bartonella apis	100.00	0.00	ftp://ftp.ncbi.nlm.nih.gov/genomes/all/GCF/002/007/565/GCF_002007565.1_ASM200756v1
GCF_002022415.1	Bartonella	Bartonella sp.	99.38	2.47	ftp://ftp.ncbi.nlm.nih.gov/genomes/all/GCF/002/022/415/GCF_002022415.1_ASM202241v1
GCF_002022445.1	Bartonella	Bartonella sp.	97.36	1.25	ftp://ftp.ncbi.nlm.nih.gov/genomes/all/GCF/002/022/445/GCF_002022445.1_ASM202244v1
GCF_002022465.1	Bartonella	Bartonella taylorii	100.00	0.00	ftp://ftp.ncbi.nlm.nih.gov/genomes/all/GCF/002/022/465/GCF_002022465.1_ASM202246v1
GCF_002022485.1	Bartonella	Bartonella sp.	98.43	0.00	ftp://ftp.ncbi.nlm.nih.gov/genomes/all/GCF/002/022/485/GCF_002022485.1_ASM202248v1
GCF_002022505.1	Bartonella	Bartonella sp.	98.77	0.41	ftp://ftp.ncbi.nlm.nih.gov/genomes/all/GCF/002/022/505/GCF_002022505.1_ASM202250v1
GCF_002022545.1	Bartonella	Bartonella sp.	98.57	0.83	ftp://ftp.ncbi.nlm.nih.gov/genomes/all/GCF/002/022/545/GCF_002022545.1_ASM202254v1
GCF_002022565.1	Bartonella	Bartonella sp.	98.78	0.42	ftp://ftp.ncbi.nlm.nih.gov/genomes/all/GCF/002/022/565/GCF_002022565.1_ASM202256v1
GCF_002022585.1	Bartonella	Bartonella sp.	98.36	0.83	ftp://ftp.ncbi.nlm.nih.gov/genomes/all/GCF/002/022/585/GCF_002022585.1_ASM202258v1
GCF_002022605.1	Bartonella	Bartonella sp.	97.98	0.00	ftp://ftp.ncbi.nlm.nih.gov/genomes/all/GCF/002/022/605/GCF_002022605.1_ASM202260v1
GCF_002022625.1	Bartonella	Bartonella sp.	98.99	1.25	ftp://ftp.ncbi.nlm.nih.gov/genomes/all/GCF/002/022/625/GCF_002022625.1_ASM202262v1
GCF_002022645.1	Bartonella	Bartonella sp.	97.53	0.83	ftp://ftp.ncbi.nlm.nih.gov/genomes/all/GCF/002/022/645/GCF_002022645.1_ASM202264v1
GCF_002022665.1	Bartonella	Bartonella sp.	99.06	0.00	ftp://ftp.ncbi.nlm.nih.gov/genomes/all/GCF/002/022/665/GCF_002022665.1_ASM202266v1
GCF_002022685.1	Bartonella	Bartonella schoenbuchensis	97.68	0.41	ftp://ftp.ncbi.nlm.nih.gov/genomes/all/GCF/002/022/685/GCF_002022685.1_ASM202268v1
GCF_002022705.1	Bartonella	Bartonella elizabethae	100.00	0.00	ftp://ftp.ncbi.nlm.nih.gov/genomes/all/GCF/002/022/705/GCF_002022705.1_ASM202270v1
GCF_002735245.1	Bartonella	Bartonella henselae	100.00	0.00	ftp://ftp.ncbi.nlm.nih.gov/genomes/all/GCF/002/735/245/GCF_002735245.1_ASM273524v1
GCF_002777915.1	Bartonella	Bartonella tribocorum	99.91	0.53	ftp://ftp.ncbi.nlm.nih.gov/genomes/all/GCF/002/777/915/GCF_002777915.1_ASM277791v1
GCF_002778015.1	Bartonella	Bartonella tribocorum	99.91	0.31	ftp://ftp.ncbi.nlm.nih.gov/genomes/all/GCF/002/778/015/GCF_002778015.1_ASM277801v1
GCF_002810325.1	Bartonella	Bartonella sp.	98.99	0.00	ftp://ftp.ncbi.nlm.nih.gov/genomes/all/GCF/002/810/325/GCF_002810325.1_ASM281032v1
GCF_002891345.1	Bartonella	Bartonella bovis	99.38	0.41	ftp://ftp.ncbi.nlm.nih.gov/genomes/all/GCF/002/891/345/GCF_002891345.1_ASM289134v1
GCF_003606325.2	Bartonella	Bartonella kosoyi	100.00	0.44	ftp://ftp.ncbi.nlm.nih.gov/genomes/all/GCF/003/606/325/GCF_003606325.2_ASM360632v3
GCF_003606345.3	Bartonella	Bartonella krasnovii	99.93	0.18	ftp://ftp.ncbi.nlm.nih.gov/genomes/all/GCF/003/606/345/GCF_003606345.3_ASM360634v3
GCF_009498695.1	Bartonella	Bartonella bacilliformis	97.09	0.00	ftp://ftp.ncbi.nlm.nih.gov/genomes/all/GCF/009/498/695/GCF_009498695.1_ASM949869v1

GCF_009936175.1	Bartonella	Bartonella quintana	99.10	0.00	ftp://ftp.ncbi.nlm.nih.gov/genomes/all/GCF/009/936/175/GCF_009936175.1_ASM993617v1
GCF_013340225.1	Bartonella	Bartonella henselae	100.00	0.00	ftp://ftp.ncbi.nlm.nih.gov/genomes/all/GCF/013/340/225/GCF_013340225.1_ASM1334022v1
GCF_013340245.1	Bartonella	Bartonella henselae	100.00	0.00	ftp://ftp.ncbi.nlm.nih.gov/genomes/all/GCF/013/340/245/GCF_013340245.1_ASM1334024v1
GCF_013388295.1	Bartonella	Bartonella alsatica	99.80	0.44	ftp://ftp.ncbi.nlm.nih.gov/genomes/all/GCF/013/388/295/GCF_013388295.1_ASM1338829v1
GCF_014117425.1	Bartonella	Bartonella sp.	99.57	0.00	ftp://ftp.ncbi.nlm.nih.gov/genomes/all/GCF/014/117/425/GCF_014117425.1_ASM1411742v1
GCF_014138465.1	Bartonella	Bartonella chomelii	99.59	0.00	ftp://ftp.ncbi.nlm.nih.gov/genomes/all/GCF/014/138/465/GCF_014138465.1_ASM1413846v1
GCF_014197255.1	Bartonella	Bartonella fuyuanensis	98.38	0.22	ftp://ftp.ncbi.nlm.nih.gov/genomes/all/GCF/014/197/255/GCF_014197255.1_ASM1419725v1
GCF_014201435.1	Bartonella	Bartonella doshiae	100.00	0.00	ftp://ftp.ncbi.nlm.nih.gov/genomes/all/GCF/014/201/435/GCF_014201435.1_ASM1420143v1
GCF_014203215.1	Bartonella	Bartonella callosciuri	99.47	0.88	ftp://ftp.ncbi.nlm.nih.gov/genomes/all/GCF/014/203/215/GCF_014203215.1_ASM1420321v1
GCF_016100335.1	Bartonella	Bartonella sp.	100.00	0.00	ftp://ftp.ncbi.nlm.nih.gov/genomes/all/GCF/016/100/335/GCF_016100335.1_ASM1610033v1
GCF_016100345.1	Bartonella	Bartonella sp.	100.00	0.00	ftp://ftp.ncbi.nlm.nih.gov/genomes/all/GCF/016/100/345/GCF_016100345.1_ASM1610034v1
GCF_016100385.1	Bartonella	Bartonella sp.	100.00	0.00	ftp://ftp.ncbi.nlm.nih.gov/genomes/all/GCF/016/100/385/GCF_016100385.1_ASM1610038v1
GCF_016100395.1	Bartonella	Bartonella sp.	100.00	0.00	ftp://ftp.ncbi.nlm.nih.gov/genomes/all/GCF/016/100/395/GCF_016100395.1_ASM1610039v1
GCF_016100425.1	Bartonella	Bartonella sp.	100.00	0.00	ftp://ftp.ncbi.nlm.nih.gov/genomes/all/GCF/016/100/425/GCF_016100425.1_ASM1610042v1
GCF_016100455.1	Bartonella	Bartonella sp.	100.00	0.00	ftp://ftp.ncbi.nlm.nih.gov/genomes/all/GCF/016/100/455/GCF_016100455.1_ASM1610045v1
GCF_016100835.1	Bartonella	Bartonella sp.	100.00	0.00	ftp://ftp.ncbi.nlm.nih.gov/genomes/all/GCF/016/100/835/GCF_016100835.1_ASM1610083v1
GCF_016102265.1	Bartonella	Bartonella sp.	100.00	0.00	ftp://ftp.ncbi.nlm.nih.gov/genomes/all/GCF/016/102/265/GCF_016102265.1_ASM1610226v1
GCF_016102285.1	Bartonella	Bartonella sp.	100.00	0.00	ftp://ftp.ncbi.nlm.nih.gov/genomes/all/GCF/016/102/285/GCF_016102285.1_ASM1610228v1
GCF_016102305.1	Bartonella	Bartonella sp.	100.00	0.00	ftp://ftp.ncbi.nlm.nih.gov/genomes/all/GCF/016/102/305/GCF_016102305.1_ASM1610230v1
GCF_016102325.1	Bartonella	Bartonella sp.	100.00	0.00	ftp://ftp.ncbi.nlm.nih.gov/genomes/all/GCF/016/102/325/GCF_016102325.1_ASM1610232v1
GCF_016102335.1	Bartonella	Bartonella sp.	98.70	0.22	ftp://ftp.ncbi.nlm.nih.gov/genomes/all/GCF/016/102/335/GCF_016102335.1_ASM1610233v1
GCF_016102355.1	Bartonella	Bartonella sp.	100.00	0.00	ftp://ftp.ncbi.nlm.nih.gov/genomes/all/GCF/016/102/355/GCF_016102355.1_ASM1610235v1
GCF_016102375.1	Bartonella	Bartonella sp.	100.00	0.00	ftp://ftp.ncbi.nlm.nih.gov/genomes/all/GCF/016/102/375/GCF_016102375.1_ASM1610237v1
GCF_016102405.1	Bartonella	Bartonella sp.	100.00	0.00	ftp://ftp.ncbi.nlm.nih.gov/genomes/all/GCF/016/102/405/GCF_016102405.1_ASM1610240v1
GCF_016102425.1	Bartonella	Bartonella sp.	100.00	0.00	ftp://ftp.ncbi.nlm.nih.gov/genomes/all/GCF/016/102/425/GCF_016102425.1_ASM1610242v1
GCF_016102435.1	Bartonella	Bartonella sp.	100.00	0.00	ftp://ftp.ncbi.nlm.nih.gov/genomes/all/GCF/016/102/435/GCF_016102435.1_ASM1610243v1
GCF_016102465.1	Bartonella	Bartonella sp.	100.00	0.00	ftp://ftp.ncbi.nlm.nih.gov/genomes/all/GCF/016/102/465/GCF_016102465.1_ASM1610246v1
GCF_016102485.1	Bartonella	Bartonella sp.	100.00	0.00	ftp://ftp.ncbi.nlm.nih.gov/genomes/all/GCF/016/102/485/GCF_016102485.1_ASM1610248v1
GCF_016102505.1	Bartonella	Bartonella sp.	100.00	0.00	ftp://ftp.ncbi.nlm.nih.gov/genomes/all/GCF/016/102/505/GCF_016102505.1_ASM1610250v1
GCF_016102525.1	Bartonella	Bartonella sp.	100.00	0.00	ftp://ftp.ncbi.nlm.nih.gov/genomes/all/GCF/016/102/525/GCF_016102525.1_ASM1610252v1
GCF_018155095.1	Bartonella	Bartonella queenslandensis	100.00	0.63	ftp://ftp.ncbi.nlm.nih.gov/genomes/all/GCF/018/155/095/GCF_018155095.1_ASM1815509v1
GCF_900185775.1	Bartonella	Bartonella mastomydis	99.89	0.09	ftp://ftp.ncbi.nlm.nih.gov/genomes/all/GCF/900/185/775/GCF_900185775.1_PRJEB21083
GCF_900445535.1	Bartonella	Bartonella doshiae	99.59	1.24	ftp://ftp.ncbi.nlm.nih.gov/genomes/all/GCF/900/445/535/GCF_900445535.1_57948_H01
GCF_900445635.1	Bartonella	Bartonella grahamii	99.89	0.44	ftp://ftp.ncbi.nlm.nih.gov/genomes/all/GCF/900/445/635/GCF_900445635.1_51184_C02
GCF_900445685.1	Bartonella	Bartonella washoeensis	82.00	2.28	ftp://ftp.ncbi.nlm.nih.gov/genomes/all/GCF/900/445/685/GCF_900445685.1_59178_H02
GCF_900475345.1	Bartonella	Bartonella quintana	99.18	0.00	ftp://ftp.ncbi.nlm.nih.gov/genomes/all/GCF/900/475/345/GCF_900475345.1_42731_H01

GCF_900 638615.1	Bartonella	Bartonella elizabethae	100.00	0.00	ftp://ftp.ncbi.nlm.nih.gov/genomes/all/GCF/900/638/615/GCF_900638 615.1_58174_F01
GCF_900 638635.1	Bartonella	Bartonella vinsonii	100.00	1.22	ftp://ftp.ncbi.nlm.nih.gov/genomes/all/GCF/900/638/635/GCF_900638 635.1_58174_G01
GCF_902 150025.1	Bartonella	Bartonella massiliensis	99.93	0.36	ftp://ftp.ncbi.nlm.nih.gov/genomes/all/GCF/902/150/025/GCF_902150 025.1_PRJEB33160
GCF_902 652675.1	Bartonella	Bartonella sp.	99.56	0.20	ftp://ftp.ncbi.nlm.nih.gov/genomes/all/GCF/902/652/675/GCF_902652 675.1_B191
GCF_902 728055.1	Bartonella	Bartonella doshiae	98.50	0.41	ftp://ftp.ncbi.nlm.nih.gov/genomes/all/GCF/902/728/055/GCF_902728 055.1_Bartonella_doshiae_BM1374167
GCF_902 728065.1	Bartonella	Bartonella doshiae	98.50	0.54	ftp://ftp.ncbi.nlm.nih.gov/genomes/all/GCF/902/728/065/GCF_902728 065.1_Bartonella_doshiae_SRS24
GCF_902 728075.1	Bartonella	Bartonella doshiae	98.50	0.41	ftp://ftp.ncbi.nlm.nih.gov/genomes/all/GCF/902/728/075/GCF_902728 075.1_Bartonella_doshiae_A14JPB
GCF_902 728085.1	Bartonella	Bartonella henselae	100.00	0.00	ftp://ftp.ncbi.nlm.nih.gov/genomes/all/GCF/902/728/085/GCF_902728 085.1_Bartonella_henselae_BM1374165
GCF_902 728115.1	Bartonella	Bartonella doshiae	100.00	0.41	ftp://ftp.ncbi.nlm.nih.gov/genomes/all/GCF/902/728/115/GCF_902728 115.1_Bartonella_doshiae_CCUG50770
GCF_902 728125.1	Bartonella	Bartonella alsatica	99.71	4.27	ftp://ftp.ncbi.nlm.nih.gov/genomes/all/GCF/902/728/125/GCF_902728 125.1_Bartonella_alsatica_IBS382T_CIP105477
GCF_902 728135.1	Bartonella	Bartonella henselae	100.00	0.31	ftp://ftp.ncbi.nlm.nih.gov/genomes/all/GCF/902/728/135/GCF_902728 135.1_Bartonella_henselae_BM1374163
GCF_902 728145.1	Bartonella	Bartonella henselae	100.00	0.00	ftp://ftp.ncbi.nlm.nih.gov/genomes/all/GCF/902/728/145/GCF_902728 145.1_Bartonella_henselae_BM1374164
GCF_902 810535.1	Bartonella	Bartonella taylorii	100.00	0.44	ftp://ftp.ncbi.nlm.nih.gov/genomes/all/GCF/902/810/535/GCF_902810 535.1_Bartonella_taylorii_SRS19
GCF_902 810545.1	Bartonella	Bartonella schoenbuche nsis	100.00	0.72	ftp://ftp.ncbi.nlm.nih.gov/genomes/all/GCF/902/810/545/GCF_902810 545.1_Bartonella_schoenbuchensis_CCUG50783
GCF_902 810555.1	Bartonella	Bartonella quintana	99.18	0.00	ftp://ftp.ncbi.nlm.nih.gov/genomes/all/GCF/902/810/555/GCF_902810 555.1_Bartonella_quintana_CCUG45777
GCF_902 810575.1	Bartonella	Bartonella taylorii	100.00	1.50	ftp://ftp.ncbi.nlm.nih.gov/genomes/all/GCF/902/810/575/GCF_902810 575.1_Bartonella_taylorii_A12JPB
GCF_902 810585.1	Bartonella	Bartonella taylorii	99.56	2.86	ftp://ftp.ncbi.nlm.nih.gov/genomes/all/GCF/902/810/585/GCF_902810 585.1_Bartonella_taylorii_SRS10
GCF_902 810605.1	Bartonella	Bartonella tribocorum	99.91	1.40	ftp://ftp.ncbi.nlm.nih.gov/genomes/all/GCF/902/810/605/GCF_902810 605.1_Bartonella_tribocorum_L103
GCF_902 810615.1	Bartonella	Bartonella tribocorum	100.00	1.07	ftp://ftp.ncbi.nlm.nih.gov/genomes/all/GCF/902/810/615/GCF_902810 615.1_Bartonella_tribocorum_BM1374166
GCF_902 810625.1	Bartonella	Bartonella tribocorum	99.68	0.88	ftp://ftp.ncbi.nlm.nih.gov/genomes/all/GCF/902/810/625/GCF_902810 625.1_Bartonella_tribocorum_C635
GCF_902 825115.1	Bartonella	Bartonella vinsonii	99.78	0.00	ftp://ftp.ncbi.nlm.nih.gov/genomes/all/GCF/902/825/115/GCF_902825 115.1_Bartonella_vinsonii_subsp_arupensis_ATCC_700727
GCF_902 825125.1	Bartonella	Bartonella koehlerae	99.59	0.00	ftp://ftp.ncbi.nlm.nih.gov/genomes/all/GCF/902/825/125/GCF_902825 125.1_Bartonella_koehlerae_CCUG_50773
GCF_902 825135.1	Bartonella	Bartonella elizabethae	99.91	0.00	ftp://ftp.ncbi.nlm.nih.gov/genomes/all/GCF/902/825/135/GCF_902825 135.1_Bartonella_elizabethae_ATCC_49927
GCF_902 825145.1	Bartonella	Bartonella phoceensis	99.82	0.25	ftp://ftp.ncbi.nlm.nih.gov/genomes/all/GCF/902/825/145/GCF_902825 145.1_Bartonella_phoceencis_CIP_107707
GCF_902 825155.1	Bartonella	Bartonella taylorii	100.00	3.73	ftp://ftp.ncbi.nlm.nih.gov/genomes/all/GCF/902/825/155/GCF_902825 155.1_Bartonella_taylorii_SRS29
GCF_902 825235.1	Bartonella	Bartonella vinsonii	99.98	1.68	ftp://ftp.ncbi.nlm.nih.gov/genomes/all/GCF/902/825/235/GCF_902825 235.1_Bartonella_vinsonii_subsp_vinsonii_CIP_103738
GCF_903 679515.1	Bartonella	Bartonella gabonensis	99.93	0.31	ftp://ftp.ncbi.nlm.nih.gov/genomes/all/GCF/903/679/515/GCF_903679 515.1_669
GCF_003 688305.1	Bififobact er	Bifidobacteri um sp.	97.64	1.33	ftp://ftp.ncbi.nlm.nih.gov/genomes/all/GCF/003/688/305/GCF_003688 305.1_ASM368830v1
GCF_000 967185.1	Bififobact er	Bifidobacteri um asteroides	97.03	0.87	ftp://ftp.ncbi.nlm.nih.gov/genomes/all/GCF/000/967/185/GCF_000967 185.1_ASM96718v1
GCF_003 688325.1	Bififobact er	Bifidobacteri um sp.	97.12	2.12	ftp://ftp.ncbi.nlm.nih.gov/genomes/all/GCF/003/688/325/GCF_003688 325.1_ASM368832v1
GCF_000 706765.1	Bififobact er	Bifidobacteri um indicum	96.31	0.61	ftp://ftp.ncbi.nlm.nih.gov/genomes/all/GCF/000/706/765/GCF_000706 765.1_ASM70676v1
GCF_000 771325.1	Bififobact er	Bifidobacteri um indicum	96.31	0.76	ftp://ftp.ncbi.nlm.nih.gov/genomes/all/GCF/000/771/325/GCF_000771 325.1_DSM-20214
GCF_003 202665.1	Bififobact er	Bifidobacteri um indicum	96.31	0.76	ftp://ftp.ncbi.nlm.nih.gov/genomes/all/GCF/003/202/665/GCF_003202 665.1_ASM320266v1
GCF_002 003665.1	Bombella	Bombella intestini	99.55	0.00	ftp://ftp.ncbi.nlm.nih.gov/genomes/all/GCF/002/003/665/GCF_002003 665.1_ASM200366v1
GCF_009 362775.1	Bombella	Bombella apis	99.80	0.00	ftp://ftp.ncbi.nlm.nih.gov/genomes/all/GCF/009/362/775/GCF_009362 775.1_ASM936277v1
GCF_009 725755.1	Bombella	Bombella sp.	99.30	0.25	ftp://ftp.ncbi.nlm.nih.gov/genomes/all/GCF/009/725/755/GCF_009725 755.1_ASM972575v1

GCF_009725845.1	Bombella	Bombella sp.	99.30	0.25	ftp://ftp.ncbi.nlm.nih.gov/genomes/all/GCF/009/725/845/GCF_009725845.1_ASM972584v1
GCF_009906835.1	Bombella	Bombella sp.	96.77	3.23	ftp://ftp.ncbi.nlm.nih.gov/genomes/all/GCF/009/906/835/GCF_009906835.1_ASM990683v1
GCF_014048465.1	Bombella	Bombella mellum	99.30	0.00	ftp://ftp.ncbi.nlm.nih.gov/genomes/all/GCF/014/048/465/GCF_014048465.1_ASM1404846v1
GCF_014048475.1	Bombella	Bombella favorum	99.80	0.00	ftp://ftp.ncbi.nlm.nih.gov/genomes/all/GCF/014/048/475/GCF_014048475.1_ASM1404847v1
GCF_014878255.1	Bombella	Bombella apis	99.70	0.00	ftp://ftp.ncbi.nlm.nih.gov/genomes/all/GCF/014/878/255/GCF_014878255.1_ASM1487825v1
GCF_018221685.1	Bombella	Bombella apis	99.70	0.00	ftp://ftp.ncbi.nlm.nih.gov/genomes/all/GCF/018/221/685/GCF_018221685.1_ASM1822168v1
GCF_000723565.1	Bombella	Lactobacillus kullabergensis	99.80	0.00	ftp://ftp.ncbi.nlm.nih.gov/genomes/all/GCF/000/723/565/GCF_000723565.1_SACS
GCF_002079945.1	Bombella	Lactobacillus sp.	99.80	0.00	ftp://ftp.ncbi.nlm.nih.gov/genomes/all/GCF/002/079/945/GCF_002079945.1_ASM207994v1
GCF_002592045.1	Bombella	Parasaccharibacter apium	98.81	0.25	ftp://ftp.ncbi.nlm.nih.gov/genomes/all/GCF/002/592/045/GCF_002592045.1_ASM259204v1
GCF_002917945.1	Bombella	Parasaccharibacter apium	99.80	0.00	ftp://ftp.ncbi.nlm.nih.gov/genomes/all/GCF/002/917/945/GCF_002917945.1_ASM291794v1
GCF_002917985.1	Bombella	Parasaccharibacter apium	99.80	0.25	ftp://ftp.ncbi.nlm.nih.gov/genomes/all/GCF/002/917/985/GCF_002917985.1_ASM291798v1
GCF_002917995.1	Bombella	Parasaccharibacter apium	99.80	0.00	ftp://ftp.ncbi.nlm.nih.gov/genomes/all/GCF/002/917/995/GCF_002917995.1_ASM291799v1
GCF_000231445.1	Commensalibacter	Commensalibacter intestini	100.00	0.50	ftp://ftp.ncbi.nlm.nih.gov/genomes/all/GCF/000/231/445/GCF_000231445.1_ASM23144v1
GCF_000527695.1	Commensalibacter	Commensalibacter sp.	99.50	0.75	ftp://ftp.ncbi.nlm.nih.gov/genomes/all/GCF/000/527/695/GCF_000527695.1_ASM52769v1
GCF_002153535.1	Commensalibacter	Commensalibacter intestini	99.50	0.50	ftp://ftp.ncbi.nlm.nih.gov/genomes/all/GCF/002/153/535/GCF_002153535.1_ASM215353v1
GCF_003202795.1	Commensalibacter	Commensalibacter sp.	99.00	0.50	ftp://ftp.ncbi.nlm.nih.gov/genomes/all/GCF/003/202/795/GCF_003202795.1_ASM320279v1
GCF_003691365.1	Commensalibacter	Commensalibacter sp.	99.00	0.75	ftp://ftp.ncbi.nlm.nih.gov/genomes/all/GCF/003/691/365/GCF_003691365.1_ASM369136v1
GCF_009725705.1	Commensalibacter	Commensalibacter sp.	99.00	0.50	ftp://ftp.ncbi.nlm.nih.gov/genomes/all/GCF/009/725/705/GCF_009725705.1_ASM972570v1
GCF_009725725.1	Commensalibacter	Commensalibacter sp.	99.00	0.75	ftp://ftp.ncbi.nlm.nih.gov/genomes/all/GCF/009/725/725/GCF_009725725.1_ASM972572v1
GCF_009725775.1	Commensalibacter	Commensalibacter sp.	99.00	0.50	ftp://ftp.ncbi.nlm.nih.gov/genomes/all/GCF/009/725/775/GCF_009725775.1_ASM972577v1
GCF_009725825.1	Commensalibacter	Commensalibacter sp.	99.00	0.50	ftp://ftp.ncbi.nlm.nih.gov/genomes/all/GCF/009/725/825/GCF_009725825.1_ASM972582v1
GCF_009725835.1	Commensalibacter	Commensalibacter sp.	99.50	0.75	ftp://ftp.ncbi.nlm.nih.gov/genomes/all/GCF/009/725/835/GCF_009725835.1_ASM972583v1
GCF_009725885.1	Commensalibacter	Commensalibacter sp.	99.00	0.50	ftp://ftp.ncbi.nlm.nih.gov/genomes/all/GCF/009/725/885/GCF_009725885.1_ASM972588v1
GCF_009734185.1	Commensalibacter	Commensalibacter sp.	99.00	0.50	ftp://ftp.ncbi.nlm.nih.gov/genomes/all/GCF/009/734/185/GCF_009734185.1_ASM973418v1
GCF_016100325.1	Commensalibacter	Commensalibacter sp.	99.00	0.50	ftp://ftp.ncbi.nlm.nih.gov/genomes/all/GCF/016/100/325/GCF_016100325.1_ASM1610032v1
GCF_016101315.1	Commensalibacter	Commensalibacter sp.	99.00	0.50	ftp://ftp.ncbi.nlm.nih.gov/genomes/all/GCF/016/101/315/GCF_016101315.1_ASM1610131v1
GCF_016101335.1	Commensalibacter	Commensalibacter sp.	99.00	0.50	ftp://ftp.ncbi.nlm.nih.gov/genomes/all/GCF/016/101/335/GCF_016101335.1_ASM1610133v1
GCF_016101365.1	Commensalibacter	Commensalibacter sp.	99.00	0.50	ftp://ftp.ncbi.nlm.nih.gov/genomes/all/GCF/016/101/365/GCF_016101365.1_ASM1610136v1
GCF_016101405.1	Commensalibacter	Commensalibacter sp.	99.00	0.50	ftp://ftp.ncbi.nlm.nih.gov/genomes/all/GCF/016/101/405/GCF_016101405.1_ASM1610140v1
GCF_016101485.1	Commensalibacter	Commensalibacter sp.	99.00	0.50	ftp://ftp.ncbi.nlm.nih.gov/genomes/all/GCF/016/101/485/GCF_016101485.1_ASM1610148v1
GCF_016101515.1	Commensalibacter	Commensalibacter sp.	99.00	0.50	ftp://ftp.ncbi.nlm.nih.gov/genomes/all/GCF/016/101/515/GCF_016101515.1_ASM1610151v1
GCF_016101565.1	Commensalibacter	Commensalibacter sp.	99.00	0.50	ftp://ftp.ncbi.nlm.nih.gov/genomes/all/GCF/016/101/565/GCF_016101565.1_ASM1610156v1
GCF_016101815.1	Commensalibacter	Commensalibacter sp.	99.00	0.50	ftp://ftp.ncbi.nlm.nih.gov/genomes/all/GCF/016/101/815/GCF_016101815.1_ASM1610181v1
GCF_016101865.1	Commensalibacter	Commensalibacter sp.	99.00	0.50	ftp://ftp.ncbi.nlm.nih.gov/genomes/all/GCF/016/101/865/GCF_016101865.1_ASM1610186v1
GCF_016101885.1	Commensalibacter	Commensalibacter sp.	99.00	0.50	ftp://ftp.ncbi.nlm.nih.gov/genomes/all/GCF/016/101/885/GCF_016101885.1_ASM1610188v1

GCF_016101905.1	Commensalibacter	Commensalibacter sp.	99.00	0.75	ftp://ftp.ncbi.nlm.nih.gov/genomes/all/GCF/016/101/905/GCF_016101905.1_ASM1610190v1
GCF_016101925.1	Commensalibacter	Commensalibacter sp.	99.00	0.75	ftp://ftp.ncbi.nlm.nih.gov/genomes/all/GCF/016/101/925/GCF_016101925.1_ASM1610192v1
GCF_016101935.1	Commensalibacter	Commensalibacter sp.	99.00	0.75	ftp://ftp.ncbi.nlm.nih.gov/genomes/all/GCF/016/101/935/GCF_016101935.1_ASM1610193v1
GCF_016101965.1	Commensalibacter	Commensalibacter sp.	99.00	0.75	ftp://ftp.ncbi.nlm.nih.gov/genomes/all/GCF/016/101/965/GCF_016101965.1_ASM1610196v1
GCF_016101975.1	Commensalibacter	Commensalibacter sp.	99.00	0.75	ftp://ftp.ncbi.nlm.nih.gov/genomes/all/GCF/016/101/975/GCF_016101975.1_ASM1610197v1
GCF_016102145.1	Commensalibacter	Commensalibacter sp.	99.00	0.50	ftp://ftp.ncbi.nlm.nih.gov/genomes/all/GCF/016/102/145/GCF_016102145.1_ASM1610214v1
GCF_000807275.1	Frischella	Frischella perrara	98.31	0.56	ftp://ftp.ncbi.nlm.nih.gov/genomes/all/GCF/000/807/275/GCF_000807275.1_ASM80727v1
GCF_003182045.1	Frischella	Frischella perrara	97.74	0.56	ftp://ftp.ncbi.nlm.nih.gov/genomes/all/GCF/003/182/045/GCF_003182045.1_ASM318204v1
GCF_003202705.1	Frischella	Frischella perrara	98.31	0.85	ftp://ftp.ncbi.nlm.nih.gov/genomes/all/GCF/003/202/705/GCF_003202705.1_ASM320270v1
GCF_014489845.1	Frischella	Frischella japonica	98.31	0.00	ftp://ftp.ncbi.nlm.nih.gov/genomes/all/GCF/014/489/845/GCF_014489845.1_ASM1448984v1
GCF_000599985.1	Gilliamella	Gilliamella apicola	98.87	0.56	ftp://ftp.ncbi.nlm.nih.gov/genomes/all/GCF/000/599/985/GCF_000599985.1_ASM59998v1
GCF_000695585.1	Gilliamella	Gilliamella apicola	98.87	0.00	ftp://ftp.ncbi.nlm.nih.gov/genomes/all/GCF/000/695/585/GCF_000695585.1_GilApiwkB30v1.0
GCF_000733115.1	Gilliamella	Gilliamella apicola	97.74	0.00	ftp://ftp.ncbi.nlm.nih.gov/genomes/all/GCF/000/733/115/GCF_000733115.1_GilApiwkB11v1.0
GCF_001690175.1	Gilliamella	Gilliamella apis	98.87	0.56	ftp://ftp.ncbi.nlm.nih.gov/genomes/all/GCF/001/690/175/GCF_001690175.1_ASM169017v1
GCF_001690185.1	Gilliamella	Gilliamella apicola	98.87	0.00	ftp://ftp.ncbi.nlm.nih.gov/genomes/all/GCF/001/690/185/GCF_001690185.1_ASM169018v1
GCF_001690195.1	Gilliamella	Gilliamella apicola	98.31	0.00	ftp://ftp.ncbi.nlm.nih.gov/genomes/all/GCF/001/690/195/GCF_001690195.1_ASM169019v1
GCF_001690235.1	Gilliamella	Gilliamella apicola	98.87	0.32	ftp://ftp.ncbi.nlm.nih.gov/genomes/all/GCF/001/690/235/GCF_001690235.1_ASM169023v1
GCF_001690255.1	Gilliamella	Gilliamella apicola	98.31	0.00	ftp://ftp.ncbi.nlm.nih.gov/genomes/all/GCF/001/690/255/GCF_001690255.1_ASM169025v1
GCF_001690265.1	Gilliamella	Gilliamella apicola	98.31	0.00	ftp://ftp.ncbi.nlm.nih.gov/genomes/all/GCF/001/690/265/GCF_001690265.1_ASM169026v1
GCF_001690275.1	Gilliamella	Gilliamella apicola	98.31	0.00	ftp://ftp.ncbi.nlm.nih.gov/genomes/all/GCF/001/690/275/GCF_001690275.1_ASM169027v1
GCF_001690335.1	Gilliamella	Gilliamella apicola	98.87	0.00	ftp://ftp.ncbi.nlm.nih.gov/genomes/all/GCF/001/690/335/GCF_001690335.1_ASM169033v1
GCF_001690345.1	Gilliamella	Gilliamella apicola	98.87	0.00	ftp://ftp.ncbi.nlm.nih.gov/genomes/all/GCF/001/690/345/GCF_001690345.1_ASM169034v1
GCF_001690355.1	Gilliamella	Gilliamella apicola	97.74	0.00	ftp://ftp.ncbi.nlm.nih.gov/genomes/all/GCF/001/690/355/GCF_001690355.1_ASM169035v1
GCF_001690385.1	Gilliamella	Gilliamella apicola	97.74	0.00	ftp://ftp.ncbi.nlm.nih.gov/genomes/all/GCF/001/690/385/GCF_001690385.1_ASM169038v1
GCF_001690415.1	Gilliamella	Gilliamella apicola	98.87	0.28	ftp://ftp.ncbi.nlm.nih.gov/genomes/all/GCF/001/690/415/GCF_001690415.1_ASM169041v1
GCF_001690435.1	Gilliamella	Gilliamella apicola	98.31	0.00	ftp://ftp.ncbi.nlm.nih.gov/genomes/all/GCF/001/690/435/GCF_001690435.1_ASM169043v1
GCF_001690445.1	Gilliamella	Gilliamella apicola	98.31	0.19	ftp://ftp.ncbi.nlm.nih.gov/genomes/all/GCF/001/690/445/GCF_001690445.1_ASM169044v1
GCF_001690495.1	Gilliamella	Gilliamella apicola	98.87	0.00	ftp://ftp.ncbi.nlm.nih.gov/genomes/all/GCF/001/690/495/GCF_001690495.1_ASM169049v1
GCF_001690515.1	Gilliamella	Gilliamella apicola	98.31	0.00	ftp://ftp.ncbi.nlm.nih.gov/genomes/all/GCF/001/690/515/GCF_001690515.1_ASM169051v1
GCF_001690525.1	Gilliamella	Gilliamella apicola	96.47	0.85	ftp://ftp.ncbi.nlm.nih.gov/genomes/all/GCF/001/690/525/GCF_001690525.1_ASM169052v1
GCF_001690535.1	Gilliamella	Gilliamella apicola	98.87	0.56	ftp://ftp.ncbi.nlm.nih.gov/genomes/all/GCF/001/690/535/GCF_001690535.1_ASM169053v1
GCF_001690585.1	Gilliamella	Gilliamella apicola	97.74	0.00	ftp://ftp.ncbi.nlm.nih.gov/genomes/all/GCF/001/690/585/GCF_001690585.1_ASM169058v1
GCF_001690595.1	Gilliamella	Gilliamella apicola	98.87	0.00	ftp://ftp.ncbi.nlm.nih.gov/genomes/all/GCF/001/690/595/GCF_001690595.1_ASM169059v1
GCF_001690605.1	Gilliamella	Gilliamella apicola	98.31	0.38	ftp://ftp.ncbi.nlm.nih.gov/genomes/all/GCF/001/690/605/GCF_001690605.1_ASM169060v1
GCF_001690655.1	Gilliamella	Gilliamella apicola	98.87	0.00	ftp://ftp.ncbi.nlm.nih.gov/genomes/all/GCF/001/690/655/GCF_001690655.1_ASM169065v1
GCF_001690675.1	Gilliamella	Gilliamella apicola	98.87	0.00	ftp://ftp.ncbi.nlm.nih.gov/genomes/all/GCF/001/690/675/GCF_001690675.1_ASM169067v1
GCF_001690685.1	Gilliamella	Gilliamella apicola	98.87	0.19	ftp://ftp.ncbi.nlm.nih.gov/genomes/all/GCF/001/690/685/GCF_001690685.1_ASM169068v1

GCF_001 690705.1	Gilliamell a	Gilliamella apicola	98.87	0.00	ftp://ftp.ncbi.nlm.nih.gov/genomes/all/GCF/001/690/705/GCF_001690 705.1_ASM169070v1
GCF_001 690735.1	Gilliamell a	Gilliamella apicola	98.87	0.56	ftp://ftp.ncbi.nlm.nih.gov/genomes/all/GCF/001/690/735/GCF_001690 735.1_ASM169073v1
GCF_001 690755.1	Gilliamell a	Gilliamella apis	98.87	0.56	ftp://ftp.ncbi.nlm.nih.gov/genomes/all/GCF/001/690/755/GCF_001690 755.1_ASM169075v1
GCF_001 690775.1	Gilliamell a	Gilliamella apis	98.87	0.56	ftp://ftp.ncbi.nlm.nih.gov/genomes/all/GCF/001/690/775/GCF_001690 775.1_ASM169077v1
GCF_001 690795.1	Gilliamell a	Gilliamella apicola	98.31	0.00	ftp://ftp.ncbi.nlm.nih.gov/genomes/all/GCF/001/690/795/GCF_001690 795.1_ASM169079v1
GCF_001 690815.1	Gilliamell a	Gilliamella apicola	98.87	0.00	ftp://ftp.ncbi.nlm.nih.gov/genomes/all/GCF/001/690/815/GCF_001690 815.1_ASM169081v1
GCF_001 690825.1	Gilliamell a	Gilliamella apicola	98.31	0.00	ftp://ftp.ncbi.nlm.nih.gov/genomes/all/GCF/001/690/825/GCF_001690 825.1_ASM169082v1
GCF_001 690835.1	Gilliamell a	Gilliamella apicola	98.87	0.00	ftp://ftp.ncbi.nlm.nih.gov/genomes/all/GCF/001/690/835/GCF_001690 835.1_ASM169083v1
GCF_001 690875.1	Gilliamell a	Gilliamella apicola	97.74	0.00	ftp://ftp.ncbi.nlm.nih.gov/genomes/all/GCF/001/690/875/GCF_001690 875.1_ASM169087v1
GCF_001 690895.1	Gilliamell a	Gilliamella apicola	98.31	0.00	ftp://ftp.ncbi.nlm.nih.gov/genomes/all/GCF/001/690/895/GCF_001690 895.1_ASM169089v1
GCF_001 693435.1	Gilliamell a	Gilliamella apicola	98.87	0.56	ftp://ftp.ncbi.nlm.nih.gov/genomes/all/GCF/001/693/435/GCF_001693 435.1_ASM169343v1
GCF_001 693745.1	Gilliamell a	Gilliamella apicola	98.31	0.19	ftp://ftp.ncbi.nlm.nih.gov/genomes/all/GCF/001/693/745/GCF_001693 745.1_ASM169374v1
GCF_001 693755.1	Gilliamell a	Gilliamella apicola	98.87	0.00	ftp://ftp.ncbi.nlm.nih.gov/genomes/all/GCF/001/693/755/GCF_001693 755.1_ASM169375v1
GCF_002 088795.1	Gilliamell a	Gilliamella apicola	98.87	0.56	ftp://ftp.ncbi.nlm.nih.gov/genomes/all/GCF/002/088/795/GCF_002088 795.1_ASM208879v1
GCF_002 088815.1	Gilliamell a	Gilliamella apicola	98.87	0.56	ftp://ftp.ncbi.nlm.nih.gov/genomes/all/GCF/002/088/815/GCF_002088 815.1_ASM208881v1
GCF_002 088825.1	Gilliamell a	Gilliamella apicola	98.87	0.56	ftp://ftp.ncbi.nlm.nih.gov/genomes/all/GCF/002/088/825/GCF_002088 825.1_ASM208882v1
GCF_002 088845.1	Gilliamell a	Gilliamella apicola	98.87	1.13	ftp://ftp.ncbi.nlm.nih.gov/genomes/all/GCF/002/088/845/GCF_002088 845.1_ASM208884v1
GCF_002 088855.1	Gilliamell a	Gilliamella apicola	98.87	1.13	ftp://ftp.ncbi.nlm.nih.gov/genomes/all/GCF/002/088/855/GCF_002088 855.1_ASM208885v1
GCF_002 088895.1	Gilliamell a	Gilliamella apicola	98.87	0.56	ftp://ftp.ncbi.nlm.nih.gov/genomes/all/GCF/002/088/895/GCF_002088 895.1_ASM208889v1
GCF_002 088905.1	Gilliamell a	Gilliamella apicola	98.87	0.56	ftp://ftp.ncbi.nlm.nih.gov/genomes/all/GCF/002/088/905/GCF_002088 905.1_ASM208890v1
GCF_002 141515.1	Gilliamell a	Gilliamella apicola	98.87	0.56	ftp://ftp.ncbi.nlm.nih.gov/genomes/all/GCF/002/141/515/GCF_002141 515.1_ASM214151v1
GCF_002 141555.1	Gilliamell a	Gilliamella apicola	98.87	0.56	ftp://ftp.ncbi.nlm.nih.gov/genomes/all/GCF/002/141/555/GCF_002141 555.1_ASM214155v1
GCF_002 141565.1	Gilliamell a	Gilliamella apicola	98.87	0.56	ftp://ftp.ncbi.nlm.nih.gov/genomes/all/GCF/002/141/565/GCF_002141 565.1_ASM214156v1
GCF_002 141575.1	Gilliamell a	Gilliamella apicola	98.31	1.13	ftp://ftp.ncbi.nlm.nih.gov/genomes/all/GCF/002/141/575/GCF_002141 575.1_ASM214157v1
GCF_002 141595.1	Gilliamell a	Gilliamella apicola	98.87	0.56	ftp://ftp.ncbi.nlm.nih.gov/genomes/all/GCF/002/141/595/GCF_002141 595.1_ASM214159v1
GCF_002 141635.1	Gilliamell a	Gilliamella apicola	98.87	1.13	ftp://ftp.ncbi.nlm.nih.gov/genomes/all/GCF/002/141/635/GCF_002141 635.1_ASM214163v1
GCF_002 141655.1	Gilliamell a	Gilliamella apicola	98.87	0.56	ftp://ftp.ncbi.nlm.nih.gov/genomes/all/GCF/002/141/655/GCF_002141 655.1_ASM214165v1
GCF_002 141665.1	Gilliamell a	Gilliamella apicola	98.87	0.56	ftp://ftp.ncbi.nlm.nih.gov/genomes/all/GCF/002/141/665/GCF_002141 665.1_ASM214166v1
GCF_002 141675.1	Gilliamell a	Gilliamella apicola	98.87	0.56	ftp://ftp.ncbi.nlm.nih.gov/genomes/all/GCF/002/141/675/GCF_002141 675.1_ASM214167v1
GCF_002 141715.1	Gilliamell a	Gilliamella apicola	98.87	0.56	ftp://ftp.ncbi.nlm.nih.gov/genomes/all/GCF/002/141/715/GCF_002141 715.1_ASM214171v1
GCF_002 141735.1	Gilliamell a	Gilliamella apicola	98.87	0.56	ftp://ftp.ncbi.nlm.nih.gov/genomes/all/GCF/002/141/735/GCF_002141 735.1_ASM214173v1
GCF_002 141745.1	Gilliamell a	Gilliamella apicola	98.87	0.56	ftp://ftp.ncbi.nlm.nih.gov/genomes/all/GCF/002/141/745/GCF_002141 745.1_ASM214174v1
GCF_002 141755.1	Gilliamell a	Gilliamella apicola	98.87	0.56	ftp://ftp.ncbi.nlm.nih.gov/genomes/all/GCF/002/141/755/GCF_002141 755.1_ASM214175v1
GCF_002 141785.1	Gilliamell a	Gilliamella apicola	98.87	1.13	ftp://ftp.ncbi.nlm.nih.gov/genomes/all/GCF/002/141/785/GCF_002141 785.1_ASM214178v1
GCF_002 141815.1	Gilliamell a	Gilliamella apicola	98.31	0.56	ftp://ftp.ncbi.nlm.nih.gov/genomes/all/GCF/002/141/815/GCF_002141 815.1_ASM214181v1
GCF_002 141825.1	Gilliamell a	Gilliamella apis	98.87	0.56	ftp://ftp.ncbi.nlm.nih.gov/genomes/all/GCF/002/141/825/GCF_002141 825.1_ASM214182v1
GCF_002 141855.1	Gilliamell a	Gilliamella apicola	98.87	1.13	ftp://ftp.ncbi.nlm.nih.gov/genomes/all/GCF/002/141/855/GCF_002141 855.1_ASM214185v1

GCF_002141865.1	Gilliamella	Gilliamella apis	98.87	0.56	ftp://ftp.ncbi.nlm.nih.gov/genomes/all/GCF/002/141/865/GCF_002141865.1_ASM214186v1
GCF_002141885.1	Gilliamella	Gilliamella apicola	98.87	1.13	ftp://ftp.ncbi.nlm.nih.gov/genomes/all/GCF/002/141/885/GCF_002141885.1_ASM214188v1
GCF_002141905.1	Gilliamella	Gilliamella apicola	98.87	0.56	ftp://ftp.ncbi.nlm.nih.gov/genomes/all/GCF/002/141/905/GCF_002141905.1_ASM214190v1
GCF_002141935.1	Gilliamella	Gilliamella apis	98.87	0.65	ftp://ftp.ncbi.nlm.nih.gov/genomes/all/GCF/002/141/935/GCF_002141935.1_ASM214193v1
GCF_002141945.1	Gilliamella	Gilliamella apis	98.87	0.65	ftp://ftp.ncbi.nlm.nih.gov/genomes/all/GCF/002/141/945/GCF_002141945.1_ASM214194v1
GCF_002141975.1	Gilliamella	Gilliamella apis	98.87	1.21	ftp://ftp.ncbi.nlm.nih.gov/genomes/all/GCF/002/141/975/GCF_002141975.1_ASM214197v1
GCF_002141985.1	Gilliamella	Gilliamella apis	98.87	0.65	ftp://ftp.ncbi.nlm.nih.gov/genomes/all/GCF/002/141/985/GCF_002141985.1_ASM214198v1
GCF_002142015.1	Gilliamella	Gilliamella sp.	98.87	1.13	ftp://ftp.ncbi.nlm.nih.gov/genomes/all/GCF/002/142/015/GCF_002142015.1_ASM214201v1
GCF_002142035.1	Gilliamella	Gilliamella apis	98.87	0.56	ftp://ftp.ncbi.nlm.nih.gov/genomes/all/GCF/002/142/035/GCF_002142035.1_ASM214203v1
GCF_002142055.1	Gilliamella	Gilliamella apis	98.87	0.56	ftp://ftp.ncbi.nlm.nih.gov/genomes/all/GCF/002/142/055/GCF_002142055.1_ASM214205v1
GCF_002142065.1	Gilliamella	Gilliamella apis	98.87	0.56	ftp://ftp.ncbi.nlm.nih.gov/genomes/all/GCF/002/142/065/GCF_002142065.1_ASM214206v1
GCF_002142085.1	Gilliamella	Gilliamella apis	98.87	0.65	ftp://ftp.ncbi.nlm.nih.gov/genomes/all/GCF/002/142/085/GCF_002142085.1_ASM214208v1
GCF_002142115.1	Gilliamella	Gilliamella apis	98.87	0.65	ftp://ftp.ncbi.nlm.nih.gov/genomes/all/GCF/002/142/115/GCF_002142115.1_ASM214211v1
GCF_002142135.1	Gilliamella	Gilliamella apis	98.87	0.56	ftp://ftp.ncbi.nlm.nih.gov/genomes/all/GCF/002/142/135/GCF_002142135.1_ASM214213v1
GCF_002142155.1	Gilliamella	Gilliamella apis	98.87	0.65	ftp://ftp.ncbi.nlm.nih.gov/genomes/all/GCF/002/142/155/GCF_002142155.1_ASM214215v1
GCF_002142165.1	Gilliamella	Gilliamella apis	98.87	0.65	ftp://ftp.ncbi.nlm.nih.gov/genomes/all/GCF/002/142/165/GCF_002142165.1_ASM214216v1
GCF_002142185.1	Gilliamella	Gilliamella apis	98.87	0.61	ftp://ftp.ncbi.nlm.nih.gov/genomes/all/GCF/002/142/185/GCF_002142185.1_ASM214218v1
GCF_002142215.1	Gilliamella	Gilliamella sp.	98.87	0.56	ftp://ftp.ncbi.nlm.nih.gov/genomes/all/GCF/002/142/215/GCF_002142215.1_ASM214221v1
GCF_002142225.1	Gilliamella	Gilliamella apis	98.87	0.61	ftp://ftp.ncbi.nlm.nih.gov/genomes/all/GCF/002/142/225/GCF_002142225.1_ASM214222v1
GCF_002142255.1	Gilliamella	Gilliamella apis	98.87	0.61	ftp://ftp.ncbi.nlm.nih.gov/genomes/all/GCF/002/142/255/GCF_002142255.1_ASM214225v1
GCF_002142265.1	Gilliamella	Gilliamella apicola	98.87	0.56	ftp://ftp.ncbi.nlm.nih.gov/genomes/all/GCF/002/142/265/GCF_002142265.1_ASM214226v1
GCF_002142275.1	Gilliamella	Gilliamella sp.	98.87	0.56	ftp://ftp.ncbi.nlm.nih.gov/genomes/all/GCF/002/142/275/GCF_002142275.1_ASM214227v1
GCF_002142285.1	Gilliamella	Gilliamella apicola	98.87	0.61	ftp://ftp.ncbi.nlm.nih.gov/genomes/all/GCF/002/142/285/GCF_002142285.1_ASM214228v1
GCF_002142345.1	Gilliamella	Gilliamella apicola	98.87	0.61	ftp://ftp.ncbi.nlm.nih.gov/genomes/all/GCF/002/142/345/GCF_002142345.1_ASM214234v1
GCF_003201455.1	Gilliamella	Gilliamella apicola	98.87	0.56	ftp://ftp.ncbi.nlm.nih.gov/genomes/all/GCF/003/201/455/GCF_003201455.1_ASM320145v1
GCF_003202655.1	Gilliamella	Gilliamella apicola	98.31	0.75	ftp://ftp.ncbi.nlm.nih.gov/genomes/all/GCF/003/202/655/GCF_003202655.1_ASM320265v1
GCF_003202775.1	Gilliamella	Gilliamella apis	98.87	0.56	ftp://ftp.ncbi.nlm.nih.gov/genomes/all/GCF/003/202/775/GCF_003202775.1_ASM320277v1
GCF_003202815.1	Gilliamella	Gilliamella apicola	98.87	0.56	ftp://ftp.ncbi.nlm.nih.gov/genomes/all/GCF/003/202/815/GCF_003202815.1_ASM320281v1
GCF_003202875.1	Gilliamella	Gilliamella apis	98.31	0.56	ftp://ftp.ncbi.nlm.nih.gov/genomes/all/GCF/003/202/875/GCF_003202875.1_ASM320287v1
GCF_003202915.1	Gilliamella	Gilliamella apicola	98.31	0.75	ftp://ftp.ncbi.nlm.nih.gov/genomes/all/GCF/003/202/915/GCF_003202915.1_ASM320291v1
GCF_007559145.1	Gilliamella	Gilliamella apicola	98.87	0.56	ftp://ftp.ncbi.nlm.nih.gov/genomes/all/GCF/007/559/145/GCF_007559145.1_ASM755914v1
GCF_007559165.1	Gilliamella	Gilliamella apicola	98.87	0.56	ftp://ftp.ncbi.nlm.nih.gov/genomes/all/GCF/007/559/165/GCF_007559165.1_ASM755916v1
GCF_009795745.1	Gilliamella	Gilliamella sp.	98.87	0.00	ftp://ftp.ncbi.nlm.nih.gov/genomes/all/GCF/009/795/745/GCF_009795745.1_ASM979574v1
GCF_009795755.1	Gilliamella	Gilliamella sp.	98.87	0.00	ftp://ftp.ncbi.nlm.nih.gov/genomes/all/GCF/009/795/755/GCF_009795755.1_ASM979575v1
GCF_009795785.1	Gilliamella	Gilliamella sp.	98.87	0.00	ftp://ftp.ncbi.nlm.nih.gov/genomes/all/GCF/009/795/785/GCF_009795785.1_ASM979578v1
GCF_009795795.1	Gilliamella	Gilliamella sp.	98.31	0.00	ftp://ftp.ncbi.nlm.nih.gov/genomes/all/GCF/009/795/795/GCF_009795795.1_ASM979579v1
GCF_009795805.1	Gilliamella	Gilliamella sp.	98.87	0.00	ftp://ftp.ncbi.nlm.nih.gov/genomes/all/GCF/009/795/805/GCF_009795805.1_ASM979580v1

GCF_009795845.1	Gilliamella	Gilliamella sp.	98.87	0.00	ftp://ftp.ncbi.nlm.nih.gov/genomes/all/GCF/009/795/845/GCF_009795845.1_ASM979584v1
GCF_009795865.1	Gilliamella	Gilliamella sp.	98.87	0.00	ftp://ftp.ncbi.nlm.nih.gov/genomes/all/GCF/009/795/865/GCF_009795865.1_ASM979586v1
GCF_009795885.1	Gilliamella	Gilliamella sp.	98.87	0.00	ftp://ftp.ncbi.nlm.nih.gov/genomes/all/GCF/009/795/885/GCF_009795885.1_ASM979588v1
GCF_009795895.1	Gilliamella	Gilliamella sp.	98.87	0.00	ftp://ftp.ncbi.nlm.nih.gov/genomes/all/GCF/009/795/895/GCF_009795895.1_ASM979589v1
GCF_009795905.1	Gilliamella	Gilliamella sp.	98.87	0.00	ftp://ftp.ncbi.nlm.nih.gov/genomes/all/GCF/009/795/905/GCF_009795905.1_ASM979590v1
GCF_013344985.1	Gilliamella	Gilliamella sp.	98.31	0.00	ftp://ftp.ncbi.nlm.nih.gov/genomes/all/GCF/013/344/985/GCF_013344985.1_ASM1334498v1
GCF_013345045.1	Gilliamella	Gilliamella sp.	98.87	0.00	ftp://ftp.ncbi.nlm.nih.gov/genomes/all/GCF/013/345/045/GCF_013345045.1_ASM1334504v1
GCF_013346885.1	Gilliamella	Gilliamella sp.	98.87	0.00	ftp://ftp.ncbi.nlm.nih.gov/genomes/all/GCF/013/346/885/GCF_013346885.1_ASM1334688v1
GCF_016100545.1	Gilliamella	Gilliamella sp.	98.87	0.56	ftp://ftp.ncbi.nlm.nih.gov/genomes/all/GCF/016/100/545/GCF_016100545.1_ASM1610054v1
GCF_016100565.1	Gilliamella	Gilliamella sp.	98.87	0.56	ftp://ftp.ncbi.nlm.nih.gov/genomes/all/GCF/016/100/565/GCF_016100565.1_ASM1610056v1
GCF_016101085.1	Gilliamella	Gilliamella sp.	98.87	0.56	ftp://ftp.ncbi.nlm.nih.gov/genomes/all/GCF/016/101/085/GCF_016101085.1_ASM1610108v1
GCF_016101125.1	Gilliamella	Gilliamella sp.	98.87	0.56	ftp://ftp.ncbi.nlm.nih.gov/genomes/all/GCF/016/101/125/GCF_016101125.1_ASM1610112v1
GCF_016101145.1	Gilliamella	Gilliamella sp.	98.87	0.56	ftp://ftp.ncbi.nlm.nih.gov/genomes/all/GCF/016/101/145/GCF_016101145.1_ASM1610114v1
GCF_016101285.1	Gilliamella	Gilliamella sp.	98.87	0.56	ftp://ftp.ncbi.nlm.nih.gov/genomes/all/GCF/016/101/285/GCF_016101285.1_ASM1610128v1
GCF_016101635.1	Gilliamella	Gilliamella sp.	98.87	0.56	ftp://ftp.ncbi.nlm.nih.gov/genomes/all/GCF/016/101/635/GCF_016101635.1_ASM1610163v1
GCF_016101655.1	Gilliamella	Gilliamella sp.	98.87	0.56	ftp://ftp.ncbi.nlm.nih.gov/genomes/all/GCF/016/101/655/GCF_016101655.1_ASM1610165v1
GCF_016101685.1	Gilliamella	Gilliamella sp.	98.87	0.56	ftp://ftp.ncbi.nlm.nih.gov/genomes/all/GCF/016/101/685/GCF_016101685.1_ASM1610168v1
GCF_016101705.1	Gilliamella	Gilliamella sp.	98.87	0.56	ftp://ftp.ncbi.nlm.nih.gov/genomes/all/GCF/016/101/705/GCF_016101705.1_ASM1610170v1
GCF_016101715.1	Gilliamella	Gilliamella sp.	98.87	0.56	ftp://ftp.ncbi.nlm.nih.gov/genomes/all/GCF/016/101/715/GCF_016101715.1_ASM1610171v1
GCF_016101735.1	Gilliamella	Gilliamella sp.	98.87	0.56	ftp://ftp.ncbi.nlm.nih.gov/genomes/all/GCF/016/101/735/GCF_016101735.1_ASM1610173v1
GCF_016101765.1	Gilliamella	Gilliamella sp.	98.87	0.56	ftp://ftp.ncbi.nlm.nih.gov/genomes/all/GCF/016/101/765/GCF_016101765.1_ASM1610176v1
GCF_016101785.1	Gilliamella	Gilliamella sp.	98.87	0.56	ftp://ftp.ncbi.nlm.nih.gov/genomes/all/GCF/016/101/785/GCF_016101785.1_ASM1610178v1
GCF_016101805.1	Gilliamella	Gilliamella sp.	98.87	0.56	ftp://ftp.ncbi.nlm.nih.gov/genomes/all/GCF/016/101/805/GCF_016101805.1_ASM1610180v1
GCF_016101825.1	Gilliamella	Gilliamella sp.	98.87	0.56	ftp://ftp.ncbi.nlm.nih.gov/genomes/all/GCF/016/101/825/GCF_016101825.1_ASM1610182v1
GCF_016102185.1	Gilliamella	Gilliamella sp.	98.87	0.56	ftp://ftp.ncbi.nlm.nih.gov/genomes/all/GCF/016/102/185/GCF_016102185.1_ASM1610218v1
GCF_900094935.1	Gilliamella	Gilliamella intestini	98.87	0.00	ftp://ftp.ncbi.nlm.nih.gov/genomes/all/GCF/900/094/935/GCF_900094935.1_IMG-taxon_2617270738_annotated_assembly
GCF_900094945.1	Gilliamella	Gilliamella bombicola	98.31	0.00	ftp://ftp.ncbi.nlm.nih.gov/genomes/all/GCF/900/094/945/GCF_900094945.1_IMG-taxon_2617270739_annotated_assembly
GCF_900103085.1	Gilliamella	Gilliamella mensalis	97.74	0.00	ftp://ftp.ncbi.nlm.nih.gov/genomes/all/GCF/900/103/085/GCF_900103085.1_LMG29880
GCF_900103255.1	Gilliamella	Gilliamella bombi	98.87	0.00	ftp://ftp.ncbi.nlm.nih.gov/genomes/all/GCF/900/103/255/GCF_900103255.1_LMG29879
GCF_000967245.1	Bombilactobacillus	Bombilactobacillus mellis	98.78	0.26	ftp://ftp.ncbi.nlm.nih.gov/genomes/all/GCF/000/967/245/GCF_000967245.1_ASM96724v1
GCF_013345055.1	Bombilactobacillus	Bombilactobacillus mellis	98.78	0.00	ftp://ftp.ncbi.nlm.nih.gov/genomes/all/GCF/013/345/055/GCF_013345055.1_ASM1334505v1
GCF_013346905.1	Bombilactobacillus	Bombilactobacillus mellis	98.25	0.52	ftp://ftp.ncbi.nlm.nih.gov/genomes/all/GCF/013/346/905/GCF_013346905.1_ASM1334690v1
GCF_013346925.1	Bombilactobacillus	Bombilactobacillus mellis	98.78	0.00	ftp://ftp.ncbi.nlm.nih.gov/genomes/all/GCF/013/346/925/GCF_013346925.1_ASM1334692v1
GCF_013347085.1	Bombilactobacillus	Bombilactobacillus mellis	98.78	0.79	ftp://ftp.ncbi.nlm.nih.gov/genomes/all/GCF/013/347/085/GCF_013347085.1_ASM1334708v1
GCF_000970795.1	Bombilactobacillus	Bombilactobacillus mellifer	98.95	0.00	ftp://ftp.ncbi.nlm.nih.gov/genomes/all/GCF/000/970/795/GCF_000970795.1_ASM97079v1
GCF_003692865.1	Lactobacillus-FIRM5	Lactobacillus sp.	96.59	0.00	ftp://ftp.ncbi.nlm.nih.gov/genomes/all/GCF/003/692/865/GCF_003692865.1_ASM369286v1

GCF_003 692755.1	Lactobaci llus-FIRM5	Lactobacillus sp.	96.59	0.00	ftp://ftp.ncbi.nlm.nih.gov/genomes/all/GCF/003/692/755/GCF_003692 755.1_ASM369275v1
GCF_003 692965.1	Lactobaci llus-FIRM5	Lactobacillus sp.	96.59	0.00	ftp://ftp.ncbi.nlm.nih.gov/genomes/all/GCF/003/692/965/GCF_003692 965.1_ASM369296v1
GCF_003 692825.1	Lactobaci llus-FIRM5	Lactobacillus sp.	96.59	0.09	ftp://ftp.ncbi.nlm.nih.gov/genomes/all/GCF/003/692/825/GCF_003692 825.1_ASM369282v1
GCF_003 692905.1	Lactobaci llus-FIRM5	Lactobacillus sp.	97.00	0.00	ftp://ftp.ncbi.nlm.nih.gov/genomes/all/GCF/003/692/905/GCF_003692 905.1_ASM369290v1
GCF_003 692805.1	Lactobaci llus-FIRM5	Lactobacillus sp.	97.00	0.65	ftp://ftp.ncbi.nlm.nih.gov/genomes/all/GCF/003/692/805/GCF_003692 805.1_ASM369280v1
GCF_003 692985.1	Lactobaci llus-FIRM5	Lactobacillus sp.	97.00	0.19	ftp://ftp.ncbi.nlm.nih.gov/genomes/all/GCF/003/692/985/GCF_003692 985.1_ASM369298v1
GCF_003 692775.1	Lactobaci llus-FIRM5	Lactobacillus sp.	97.00	0.19	ftp://ftp.ncbi.nlm.nih.gov/genomes/all/GCF/003/692/775/GCF_003692 775.1_ASM369277v1
GCF_003 692985.1	Lactobaci llus-FIRM5	Lactobacillus sp.	97.00	0.19	ftp://ftp.ncbi.nlm.nih.gov/genomes/all/GCF/003/692/985/GCF_003692 985.1_ASM369298v1
GCF_003 692885.1	Lactobaci llus-FIRM5	Lactobacillus sp.	97.00	0.19	ftp://ftp.ncbi.nlm.nih.gov/genomes/all/GCF/003/692/885/GCF_003692 885.1_ASM369288v1
GCF_003 692995.1	Lactobaci llus-FIRM5	Lactobacillus sp.	97.08	0.00	ftp://ftp.ncbi.nlm.nih.gov/genomes/all/GCF/003/692/995/GCF_003692 995.1_ASM369299v1
GCF_003 692845.1	Lactobaci llus-FIRM5	Lactobacillus sp.	97.40	0.76	ftp://ftp.ncbi.nlm.nih.gov/genomes/all/GCF/003/692/845/GCF_003692 845.1_ASM369284v1
GCF_003 150935.1	Lactobaci llus-FIRM5	Lactobacillus sp.	97.32	0.76	ftp://ftp.ncbi.nlm.nih.gov/genomes/all/GCF/003/150/935/GCF_003150 935.1_ASM315093v1
GCF_000 970735.1	Lactobaci llus-FIRM5	Lactobacillus sp.	97.40	0.76	ftp://ftp.ncbi.nlm.nih.gov/genomes/all/GCF/000/970/735/GCF_000970 735.1_ASM97073v1
GCF_000 761135.1	Lactobaci llus-FIRM5	Lactobacillus apis	97.73	1.14	ftp://ftp.ncbi.nlm.nih.gov/genomes/all/GCF/000/761/135/GCF_000761 135.1_ASM76113v1
GCF_003 692925.1	Lactobaci llus-FIRM5	Lactobacillus apis	97.73	1.14	ftp://ftp.ncbi.nlm.nih.gov/genomes/all/GCF/003/692/925/GCF_003692 925.1_ASM369292v1
GCF_003 173695.1	Lactobaci llus-FIRM5	Lactobacillus sp.	97.73	1.14	ftp://ftp.ncbi.nlm.nih.gov/genomes/all/GCF/003/173/695/GCF_003173 695.1_ASM317369v1
GCF_000 970855.1	Lactobaci llus-FIRM5	Lactobacillus sp.	97.65	1.14	ftp://ftp.ncbi.nlm.nih.gov/genomes/all/GCF/000/970/855/GCF_000970 855.1_ASM97085v1
GCF_003 692935.1	Lactobaci llus-FIRM5	Lactobacillus helsingborge nsis	97.40	1.08	ftp://ftp.ncbi.nlm.nih.gov/genomes/all/GCF/003/692/935/GCF_003692 935.1_ASM369293v1
GCF_003 693045.1	Lactobaci llus-FIRM5	Lactobacillus helsingborge nsis	97.40	1.70	ftp://ftp.ncbi.nlm.nih.gov/genomes/all/GCF/003/693/045/GCF_003693 045.1_ASM369304v1
GCF_003 202825.1	Lactobaci llus-FIRM5	Lactobacillus sp.	97.40	1.62	ftp://ftp.ncbi.nlm.nih.gov/genomes/all/GCF/003/202/825/GCF_003202 825.1_ASM320282v1
GCF_000 970775.1	Lactobaci llus-FIRM5	Lactobacillus sp.	97.40	1.95	ftp://ftp.ncbi.nlm.nih.gov/genomes/all/GCF/000/970/775/GCF_000970 775.1_ASM97077v1
GCF_000 970755.1	Lactobaci llus-FIRM5	Lactobacillus melliventris	97.40	1.27	ftp://ftp.ncbi.nlm.nih.gov/genomes/all/GCF/000/970/755/GCF_000970 755.1_ASM97075v1
GCF_000 967195.1	Lactobaci llus-FIRM5	Lactobacillus melliventris	96.72	1.70	ftp://ftp.ncbi.nlm.nih.gov/genomes/all/GCF/000/967/195/GCF_000967 195.1_ASM96719v1
GCF_000 760615.1	Lactobaci llus-FIRM5	Lactobacillus kimbladii	97.40	0.65	ftp://ftp.ncbi.nlm.nih.gov/genomes/all/GCF/000/760/615/GCF_000760 615.1_ASM76061v1

GCF_003151025.1	Lactobacillus-FIRM5	Lactobacillus kullabergensis	97.40	0.97	ftp://ftp.ncbi.nlm.nih.gov/genomes/all/GCF/003/151/025/GCF_003151025.1_ASM315102v1
GCF_003693025.1	Lactobacillus-FIRM5	Lactobacillus sp.	97.40	0.87	ftp://ftp.ncbi.nlm.nih.gov/genomes/all/GCF/003/693/025/GCF_003693025.1_ASM369302v1
GCF_002259585.1	Bombiscardovia	Bombiscardovia coagulans	92.43	1.16	ftp://ftp.ncbi.nlm.nih.gov/genomes/all/GCF/002/259/585/GCF_002259585.1_ASM225958v1
GCF_000741865.1	Apilactobacillus	Apilactobacillus kunkeei	98.07	1.25	ftp://ftp.ncbi.nlm.nih.gov/genomes/all/GCF/000/741/865/GCF_000741865.1_LkMP2v1.0
GCF_000830375.1	Apilactobacillus	Apilactobacillus kunkeei	97.76	2.08	ftp://ftp.ncbi.nlm.nih.gov/genomes/all/GCF/000/830/375/GCF_000830375.1_ASM83037v1
GCF_001281165.1	Apilactobacillus	Apilactobacillus kunkeei	97.76	0.94	ftp://ftp.ncbi.nlm.nih.gov/genomes/all/GCF/001/281/165/GCF_001281165.1_ASM128116v1
GCF_001281175.1	Apilactobacillus	Apilactobacillus apinorum	97.39	0.94	ftp://ftp.ncbi.nlm.nih.gov/genomes/all/GCF/001/281/175/GCF_001281175.1_ASM128117v1
GCF_001281205.1	Apilactobacillus	Apilactobacillus kunkeei	98.07	1.25	ftp://ftp.ncbi.nlm.nih.gov/genomes/all/GCF/001/281/205/GCF_001281205.1_ASM128120v1
GCF_001281215.1	Apilactobacillus	Apilactobacillus kunkeei	98.07	1.25	ftp://ftp.ncbi.nlm.nih.gov/genomes/all/GCF/001/281/215/GCF_001281215.1_ASM128121v1
GCF_001281225.1	Apilactobacillus	Apilactobacillus kunkeei	97.76	1.41	ftp://ftp.ncbi.nlm.nih.gov/genomes/all/GCF/001/281/225/GCF_001281225.1_ASM128122v1
GCF_001281265.1	Apilactobacillus	Apilactobacillus kunkeei	98.07	1.25	ftp://ftp.ncbi.nlm.nih.gov/genomes/all/GCF/001/281/265/GCF_001281265.1_ASM128126v1
GCF_001281285.1	Apilactobacillus	Apilactobacillus kunkeei	97.76	0.94	ftp://ftp.ncbi.nlm.nih.gov/genomes/all/GCF/001/281/285/GCF_001281285.1_ASM128128v1
GCF_001308185.1	Apilactobacillus	Apilactobacillus kunkeei	98.07	1.25	ftp://ftp.ncbi.nlm.nih.gov/genomes/all/GCF/001/308/185/GCF_001308185.1_ASM130818v1
GCF_001308195.1	Apilactobacillus	Apilactobacillus kunkeei	98.07	1.25	ftp://ftp.ncbi.nlm.nih.gov/genomes/all/GCF/001/308/195/GCF_001308195.1_ASM130819v1
GCF_001308205.1	Apilactobacillus	Apilactobacillus kunkeei	98.07	1.09	ftp://ftp.ncbi.nlm.nih.gov/genomes/all/GCF/001/308/205/GCF_001308205.1_ASM130820v1
GCF_001308215.1	Apilactobacillus	Apilactobacillus kunkeei	98.07	1.25	ftp://ftp.ncbi.nlm.nih.gov/genomes/all/GCF/001/308/215/GCF_001308215.1_ASM130821v1
GCF_001314945.1	Apilactobacillus	Apilactobacillus kunkeei	98.07	2.29	ftp://ftp.ncbi.nlm.nih.gov/genomes/all/GCF/001/314/945/GCF_001314945.1_ASM131494v1
GCF_001421115.1	Apilactobacillus	Apilactobacillus kunkeei	97.76	0.94	ftp://ftp.ncbi.nlm.nih.gov/genomes/all/GCF/001/421/115/GCF_001421115.1_ASM142111v1
GCF_001421135.1	Apilactobacillus	Apilactobacillus kunkeei	97.76	0.94	ftp://ftp.ncbi.nlm.nih.gov/genomes/all/GCF/001/421/135/GCF_001421135.1_ASM142113v1
GCF_001433825.1	Apilactobacillus	Apilactobacillus kunkeei	98.07	1.25	ftp://ftp.ncbi.nlm.nih.gov/genomes/all/GCF/001/433/825/GCF_001433825.1_ASM143382v1
GCF_001435995.1	Apilactobacillus	Apilactobacillus ozensis	97.45	1.25	ftp://ftp.ncbi.nlm.nih.gov/genomes/all/GCF/001/435/995/GCF_001435995.1_ASM143599v1
GCF_001949975.2	Apilactobacillus	Apilactobacillus kunkeei	98.07	0.62	ftp://ftp.ncbi.nlm.nih.gov/genomes/all/GCF/001/949/975/GCF_001949975.2_ASM194997v2
GCF_002993965.1	Apilactobacillus	Apilactobacillus timberlakei	98.65	0.00	ftp://ftp.ncbi.nlm.nih.gov/genomes/all/GCF/002/993/965/GCF_002993965.1_ASM299396v1
GCF_002993975.1	Apilactobacillus	Apilactobacillus micheneri	98.02	0.00	ftp://ftp.ncbi.nlm.nih.gov/genomes/all/GCF/002/993/975/GCF_002993975.1_ASM299397v1
GCF_002994005.1	Apilactobacillus	Apilactobacillus quenuiae	98.65	0.62	ftp://ftp.ncbi.nlm.nih.gov/genomes/all/GCF/002/994/005/GCF_002994005.1_ASM299400v1
GCF_003112615.1	Apilactobacillus	Apilactobacillus micheneri	98.65	0.00	ftp://ftp.ncbi.nlm.nih.gov/genomes/all/GCF/003/112/615/GCF_003112615.1_ASM311261v1
GCF_003627035.1	Apilactobacillus	Apilactobacillus bombintestini	97.45	0.62	ftp://ftp.ncbi.nlm.nih.gov/genomes/all/GCF/003/627/035/GCF_003627035.1_ASM362703v1
GCF_005930915.1	Apilactobacillus	Apilactobacillus kunkeei	98.07	1.25	ftp://ftp.ncbi.nlm.nih.gov/genomes/all/GCF/005/930/915/GCF_005930915.1_ASM593091v1
GCF_005930925.1	Apilactobacillus	Apilactobacillus kunkeei	98.07	1.25	ftp://ftp.ncbi.nlm.nih.gov/genomes/all/GCF/005/930/925/GCF_005930925.1_ASM593092v1
GCF_005930935.1	Apilactobacillus	Apilactobacillus kunkeei	98.07	1.09	ftp://ftp.ncbi.nlm.nih.gov/genomes/all/GCF/005/930/935/GCF_005930935.1_ASM593093v1
GCF_005930975.1	Apilactobacillus	Apilactobacillus kunkeei	98.07	1.25	ftp://ftp.ncbi.nlm.nih.gov/genomes/all/GCF/005/930/975/GCF_005930975.1_ASM593097v1
GCF_006493055.1	Apilactobacillus	Apilactobacillus timberlakei	98.02	0.00	ftp://ftp.ncbi.nlm.nih.gov/genomes/all/GCF/006/493/055/GCF_006493055.1_ASM649305v1

GCF_006 493095.1	Apilactob acillus	Apilactobacil lus timberlakei	98.65	0.54	ftp://ftp.ncbi.nlm.nih.gov/genomes/all/GCF/006/493/095/GCF_006493 095.1_ASM649309v1
GCF_006 493105.1	Apilactob acillus	Apilactobacil lus timberlakei	98.65	0.00	ftp://ftp.ncbi.nlm.nih.gov/genomes/all/GCF/006/493/105/GCF_006493 105.1_ASM649310v1
GCF_006 493125.1	Apilactob acillus	Apilactobacil lus timberlakei	98.65	0.00	ftp://ftp.ncbi.nlm.nih.gov/genomes/all/GCF/006/493/125/GCF_006493 125.1_ASM649312v1
GCF_006 493155.1	Apilactob acillus	Apilactobacil lus timberlakei	98.63	0.00	ftp://ftp.ncbi.nlm.nih.gov/genomes/all/GCF/006/493/155/GCF_006493 155.1_ASM649315v1
GCF_006 493175.1	Apilactob acillus	Apilactobacil lus timberlakei	98.65	0.00	ftp://ftp.ncbi.nlm.nih.gov/genomes/all/GCF/006/493/175/GCF_006493 175.1_ASM649317v1
GCF_006 493185.1	Apilactob acillus	Apilactobacil lus micheneri	98.65	0.00	ftp://ftp.ncbi.nlm.nih.gov/genomes/all/GCF/006/493/185/GCF_006493 185.1_ASM649318v1
GCF_006 493195.1	Apilactob acillus	Apilactobacil lus micheneri	98.65	0.00	ftp://ftp.ncbi.nlm.nih.gov/genomes/all/GCF/006/493/195/GCF_006493 195.1_ASM649319v1
GCF_006 493225.1	Apilactob acillus	Apilactobacil lus micheneri	98.65	0.00	ftp://ftp.ncbi.nlm.nih.gov/genomes/all/GCF/006/493/225/GCF_006493 225.1_ASM649322v1
GCF_006 493235.1	Apilactob acillus	Apilactobacil lus micheneri	98.65	0.00	ftp://ftp.ncbi.nlm.nih.gov/genomes/all/GCF/006/493/235/GCF_006493 235.1_ASM649323v1
GCF_006 493275.1	Apilactob acillus	Apilactobacil lus micheneri	98.65	0.00	ftp://ftp.ncbi.nlm.nih.gov/genomes/all/GCF/006/493/275/GCF_006493 275.1_ASM649327v1
GCF_006 493295.1	Apilactob acillus	Apilactobacil lus micheneri	98.02	0.00	ftp://ftp.ncbi.nlm.nih.gov/genomes/all/GCF/006/493/295/GCF_006493 295.1_ASM649329v1
GCF_006 493305.1	Apilactob acillus	Apilactobacil lus micheneri	98.65	0.00	ftp://ftp.ncbi.nlm.nih.gov/genomes/all/GCF/006/493/305/GCF_006493 305.1_ASM649330v1
GCF_006 493315.1	Apilactob acillus	Apilactobacil lus micheneri	98.65	0.00	ftp://ftp.ncbi.nlm.nih.gov/genomes/all/GCF/006/493/315/GCF_006493 315.1_ASM649331v1
GCF_006 493325.1	Apilactob acillus	Apilactobacil lus micheneri	98.65	0.00	ftp://ftp.ncbi.nlm.nih.gov/genomes/all/GCF/006/493/325/GCF_006493 325.1_ASM649332v1
GCF_006 493375.1	Apilactob acillus	Apilactobacil lus micheneri	98.65	0.00	ftp://ftp.ncbi.nlm.nih.gov/genomes/all/GCF/006/493/375/GCF_006493 375.1_ASM649337v1
GCF_006 493385.1	Apilactob acillus	Apilactobacil lus micheneri	98.65	0.00	ftp://ftp.ncbi.nlm.nih.gov/genomes/all/GCF/006/493/385/GCF_006493 385.1_ASM649338v1
GCF_006 493415.1	Apilactob acillus	Apilactobacil lus kunkeei	97.76	0.94	ftp://ftp.ncbi.nlm.nih.gov/genomes/all/GCF/006/493/415/GCF_006493 415.1_ASM649341v1
GCF_006 493425.1	Apilactob acillus	Apilactobacil lus timberlakei	98.65	0.00	ftp://ftp.ncbi.nlm.nih.gov/genomes/all/GCF/006/493/425/GCF_006493 425.1_ASM649342v1
GCF_006 493435.1	Apilactob acillus	Apilactobacil lus timberlakei	98.65	0.00	ftp://ftp.ncbi.nlm.nih.gov/genomes/all/GCF/006/493/435/GCF_006493 435.1_ASM649343v1
GCF_006 493445.1	Apilactob acillus	Apilactobacil lus micheneri	98.65	0.00	ftp://ftp.ncbi.nlm.nih.gov/genomes/all/GCF/006/493/445/GCF_006493 445.1_ASM649344v1
GCF_006 493495.1	Apilactob acillus	Apilactobacil lus micheneri	98.65	0.00	ftp://ftp.ncbi.nlm.nih.gov/genomes/all/GCF/006/493/495/GCF_006493 495.1_ASM649349v1
GCF_006 493515.1	Apilactob acillus	Apilactobacil lus micheneri	98.65	0.00	ftp://ftp.ncbi.nlm.nih.gov/genomes/all/GCF/006/493/515/GCF_006493 515.1_ASM649351v1
GCF_006 493525.1	Apilactob acillus	Apilactobacil lus micheneri	97.97	0.00	ftp://ftp.ncbi.nlm.nih.gov/genomes/all/GCF/006/493/525/GCF_006493 525.1_ASM649352v1
GCF_006 493545.1	Apilactob acillus	Apilactobacil lus micheneri	98.65	0.00	ftp://ftp.ncbi.nlm.nih.gov/genomes/all/GCF/006/493/545/GCF_006493 545.1_ASM649354v1
GCF_006 493565.1	Apilactob acillus	Apilactobacil lus micheneri	98.65	0.00	ftp://ftp.ncbi.nlm.nih.gov/genomes/all/GCF/006/493/565/GCF_006493 565.1_ASM649356v1

GCF_006 493595.1	Apilactob acillus	Apilactobacil lus micheneri	98.65	0.00	ftp://ftp.ncbi.nlm.nih.gov/genomes/all/GCF/006/493/595/GCF_006493 595.1_ASM649359v1
GCF_006 493615.1	Apilactob acillus	Apilactobacil lus kunkeei	98.07	1.25	ftp://ftp.ncbi.nlm.nih.gov/genomes/all/GCF/006/493/615/GCF_006493 615.1_ASM649361v1
GCF_006 493625.1	Apilactob acillus	Apilactobacil lus micheneri	98.65	1.57	ftp://ftp.ncbi.nlm.nih.gov/genomes/all/GCF/006/493/625/GCF_006493 625.1_ASM649362v1
GCF_009 910775.1	Apilactob acillus	Apilactobacil lus kunkeei	97.76	0.94	ftp://ftp.ncbi.nlm.nih.gov/genomes/all/GCF/009/910/775/GCF_009910 775.1_ASM991077v1
GCF_014 323645.1	Apilactob acillus	Apilactobacil lus kunkeei	98.07	1.41	ftp://ftp.ncbi.nlm.nih.gov/genomes/all/GCF/014/323/645/GCF_014323 645.1_ASM1432364v1
GCF_016 861895.1	Apilactob acillus	Apilactobacil lus sp.	98.07	1.25	ftp://ftp.ncbi.nlm.nih.gov/genomes/all/GCF/016/861/895/GCF_016861 895.1_ASM1686189v1
GCF_016 861915.1	Apilactob acillus	Apilactobacil lus sp.	98.07	1.25	ftp://ftp.ncbi.nlm.nih.gov/genomes/all/GCF/016/861/915/GCF_016861 915.1_ASM1686191v1
GCF_019 061205.1	Apilactob acillus	Apilactobacil lus sp.	98.07	0.94	ftp://ftp.ncbi.nlm.nih.gov/genomes/all/GCF/019/061/205/GCF_019061 205.1_ASM1906120v1
GCF_016 757715.1	TQMD	Listeria monocytoge nes	99.45	0.00	ftp://ftp.ncbi.nlm.nih.gov/genomes/all/GCF/016/757/715/GCF_016757 715.1_ASM1675771v1
GCF_006 740305.1	TQMD	Lactobacillus delbrueckii	99.03	0.00	ftp://ftp.ncbi.nlm.nih.gov/genomes/all/GCF/006/740/305/GCF_006740 305.1_ASM674030v1
GCF_000 023845.1	TQMD	Cryptobacter ium curtum	100.00	0.00	ftp://ftp.ncbi.nlm.nih.gov/genomes/all/GCF/000/023/845/GCF_000023 845.1_ASM2384v1
GCF_001 742205.1	TQMD	Limosilactob acillus fermentum	99.18	0.55	ftp://ftp.ncbi.nlm.nih.gov/genomes/all/GCF/001/742/205/GCF_001742 205.1_ASM174220v1
GCF_001 936335.1	TQMD	Amylolactob acillus amylophilus	98.95	5.76	ftp://ftp.ncbi.nlm.nih.gov/genomes/all/GCF/001/936/335/GCF_001936 335.1_ASM193633v1
GCF_000 732905.1	TQMD	Weissella ceti	98.04	0.00	ftp://ftp.ncbi.nlm.nih.gov/genomes/all/GCF/000/732/905/GCF_000732 905.1_ASM73290v1
GCF_001 543145.1	TQMD	Aerococcus sanguinicola	98.72	1.65	ftp://ftp.ncbi.nlm.nih.gov/genomes/all/GCF/001/543/145/GCF_001543 145.1_ASM154314v1
GCF_000 024225.1	TQMD	Atopobium parvulum	100.00	0.00	ftp://ftp.ncbi.nlm.nih.gov/genomes/all/GCF/000/024/225/GCF_000024 225.1_ASM2422v1
GCF_900 638585.1	TQMD	Peptoniphilu s ivorii	98.95	0.00	ftp://ftp.ncbi.nlm.nih.gov/genomes/all/GCF/900/638/585/GCF_900638 585.1_57845_G01
GCF_013 267415.1	TQMD	Abiotrophia defectiva	98.46	0.00	ftp://ftp.ncbi.nlm.nih.gov/genomes/all/GCF/013/267/415/GCF_013267 415.1_ASM1326741v1
GCF_000 009025.1	TQMD	Dehalococco ides mccartyi	99.01	0.00	ftp://ftp.ncbi.nlm.nih.gov/genomes/all/GCF/000/009/025/GCF_000009 025.1_ASM902v1
GCF_003 990665.1	TQMD	Thermosyne chococcus vulcanus	99.76	0.12	ftp://ftp.ncbi.nlm.nih.gov/genomes/all/GCF/003/990/665/GCF_003990 665.1_ASM399066v2
GCF_900 169485.1	TQMD	Phoenicibact er congonensis	97.38	0.00	ftp://ftp.ncbi.nlm.nih.gov/genomes/all/GCF/900/169/485/GCF_900169 485.1_PRJEB19959
GCF_900 119705.1	TQMD	Murdochiella vaginalis	97.26	0.00	ftp://ftp.ncbi.nlm.nih.gov/genomes/all/GCF/900/119/705/GCF_900119 705.1_PRJEB14245
GCF_013 393745.1	TQMD	Mycoplasma synoviae	100.00	0.94	ftp://ftp.ncbi.nlm.nih.gov/genomes/all/GCF/013/393/745/GCF_013393 745.1_ASM1339374v1
GCF_900 105895.1	TQMD	Atopobium minutum	100.00	0.00	ftp://ftp.ncbi.nlm.nih.gov/genomes/all/GCF/900/105/895/GCF_900105 895.1_IMG-taxon_2634166360_annotated_assembly
GCF_002 804205.1	TQMD	Entomoplas ma freundtii	99.24	3.03	ftp://ftp.ncbi.nlm.nih.gov/genomes/all/GCF/002/804/205/GCF_002804 205.1_ASM280420v1
GCF_007 713705.1	TQMD	Salinicoccus cyprini	99.43	0.96	ftp://ftp.ncbi.nlm.nih.gov/genomes/all/GCF/007/713/705/GCF_007713 705.1_ASM771370v1
GCF_001 558175.1	TQMD	Mycoplasma pneumoniae	99.25	0.00	ftp://ftp.ncbi.nlm.nih.gov/genomes/all/GCF/001/558/175/GCF_001558 175.1_ASM155817v1
GCF_900 240275.1	TQMD	Lactobacillus timonensis	100.00	0.54	ftp://ftp.ncbi.nlm.nih.gov/genomes/all/GCF/900/240/275/GCF_900240 275.1_PRJEB20106
GCF_000 245795.1	TQMD	Facklamia languida	98.06	0.56	ftp://ftp.ncbi.nlm.nih.gov/genomes/all/GCF/000/245/795/GCF_000245 795.1_Fack_lang_CCUG_37842_V1
GCF_013 415205.1	TQMD	Streptococcu s danieliae	94.07	0.00	ftp://ftp.ncbi.nlm.nih.gov/genomes/all/GCF/013/415/205/GCF_013415 205.1_ASM1341520v1
GCF_000 319365.1	TQMD	Mycoplasma haemominut um	66.90	0.00	ftp://ftp.ncbi.nlm.nih.gov/genomes/all/GCF/000/319/365/GCF_000319 365.1_ASM31936v1
GCF_900 660465.1	TQMD	Mycoplasma pneumoniae	99.25	1.50	ftp://ftp.ncbi.nlm.nih.gov/genomes/all/GCF/900/660/465/GCF_900660 465.1_50648_A01-3
GCF_000 277795.1	TQMD	Mycoplasma wenyonii	66.57	2.41	ftp://ftp.ncbi.nlm.nih.gov/genomes/all/GCF/000/277/795/GCF_000277 795.1_ASM27779v1

GCF_000019625.1	TQMD	Thermotoga sp.	100.00	1.79	ftp://ftp.ncbi.nlm.nih.gov/genomes/all/GCF/000/019/625/GCF_000019625.1_ASM1962v1
GCF_000304715.1	TQMD	Lactobacillus florum	97.50	0.00	ftp://ftp.ncbi.nlm.nih.gov/genomes/all/GCF/000/304/715/GCF_000304715.1_2F_v3c
GCF_900217925.1	TQMD	Limosilactobacillus mucosae	99.18	0.54	ftp://ftp.ncbi.nlm.nih.gov/genomes/all/GCF/900/217/925/GCF_900217925.1_IMG-taxon_2582581252_annotated_assembly
GCF_000281235.1	TQMD	Mycoplasma haemolamae	66.39	0.60	ftp://ftp.ncbi.nlm.nih.gov/genomes/all/GCF/000/281/235/GCF_000281235.1_ASM28123v1
GCF_000174015.1	TQMD	Atopobium rimae	100.00	0.00	ftp://ftp.ncbi.nlm.nih.gov/genomes/all/GCF/000/174/015/GCF_000174015.1_ASM17401v1
GCF_000238995.1	TQMD	Mycoplasma haemocanis	75.16	0.00	ftp://ftp.ncbi.nlm.nih.gov/genomes/all/GCF/000/238/995/GCF_000238995.1_ASM23899v1
GCF_900112405.1	TQMD	Fructobacillus durionis	99.04	1.05	ftp://ftp.ncbi.nlm.nih.gov/genomes/all/GCF/900/112/405/GCF_900112405.1_IMG-taxon_2603880187_annotated_assembly
GCF_003116835.1	TQMD	Lactococcus termiticola	98.87	0.85	ftp://ftp.ncbi.nlm.nih.gov/genomes/all/GCF/003/116/835/GCF_003116835.1_ASM311683v1
GCF_902376015.1	TQMD	Olegusella massiliensis	100.00	0.00	ftp://ftp.ncbi.nlm.nih.gov/genomes/all/GCF/902/376/015/GCF_902376015.1_MGYG-HGUT-01545
GCF_904390885.1	TQMD	Limosilactobacillus oris	98.34	0.00	ftp://ftp.ncbi.nlm.nih.gov/genomes/all/GCF/904/390/885/GCF_904390885.1_Chicken_5_mag_67
GCF_014489725.1	TQMD	Fructobacillus fructosus	98.52	0.00	ftp://ftp.ncbi.nlm.nih.gov/genomes/all/GCF/014/489/725/GCF_014489725.1_ASM1448972v1
GCF_900216215.1	TQMD	Weissella viridescens	69.33	0.00	ftp://ftp.ncbi.nlm.nih.gov/genomes/all/GCF/900/216/215/GCF_900216215.1_MFPC16A2805a-v1
GCF_001436135.1	TQMD	Lactobacillus thailandensis	98.95	1.05	ftp://ftp.ncbi.nlm.nih.gov/genomes/all/GCF/001/436/135/GCF_001436135.1_ASM143613v1
GCF_904394975.1	TQMD	Metaruminococcus gallistercoris	95.97	0.00	ftp://ftp.ncbi.nlm.nih.gov/genomes/all/GCF/904/394/975/GCF_904394975.1_Coassembly_mag_135
GCF_000420365.1	TQMD	Weissella halotolerans	99.39	0.00	ftp://ftp.ncbi.nlm.nih.gov/genomes/all/GCF/000/420/365/GCF_000420365.1_ASM42036v1
GCF_001437055.1	TQMD	Limosilactobacillus secaliphilus	98.91	0.27	ftp://ftp.ncbi.nlm.nih.gov/genomes/all/GCF/001/437/055/GCF_001437055.1_ASM143705v1
GCF_004011945.1	TQMD	Mycoplasma sp.	87.58	0.00	ftp://ftp.ncbi.nlm.nih.gov/genomes/all/GCF/004/011/945/GCF_004011945.1_ASM401194v1
GCF_011039075.1	TQMD	Adlercreutzia sp.	99.19	0.00	ftp://ftp.ncbi.nlm.nih.gov/genomes/all/GCF/011/039/075/GCF_011039075.1_ASM1103907v1
GCF_904420055.1	TQMD	Alloscillospira gallinarum	91.61	2.01	ftp://ftp.ncbi.nlm.nih.gov/genomes/all/GCF/904/420/055/GCF_904420055.1_Coassembly_mag_141
GCF_900343135.1	TQMD	Atopobium massiliense	100.00	0.00	ftp://ftp.ncbi.nlm.nih.gov/genomes/all/GCF/900/343/135/GCF_900343135.1_PRJEB24599
GCF_904398645.1	TQMD	Allobutyricicoccus pentlandensis	80.12	2.35	ftp://ftp.ncbi.nlm.nih.gov/genomes/all/GCF/904/398/645/GCF_904398645.1_Coassembly_mag_96
GCF_001856085.1	TQMD	Planococcus salinarum	33.37	0.00	ftp://ftp.ncbi.nlm.nih.gov/genomes/all/GCF/001/856/085/GCF_001856085.1_ASM185608v1
GCF_902375925.1	TQMD	Murdochiella massiliensis	97.26	0.00	ftp://ftp.ncbi.nlm.nih.gov/genomes/all/GCF/902/375/925/GCF_902375925.1_MGYG-HGUT-01541
GCF_013394805.1	TQMD	Mogibacterium timidum	98.58	0.71	ftp://ftp.ncbi.nlm.nih.gov/genomes/all/GCF/013/394/805/GCF_013394805.1_ASM1339480v1
GCF_904378995.1	TQMD	Ruminococcus sp.	92.62	0.27	ftp://ftp.ncbi.nlm.nih.gov/genomes/all/GCF/904/378/995/GCF_904378995.1_Chicken_16_mag_123
GCF_904419725.1	TQMD	Heteroclostridium caecigallinarum	94.76	0.81	ftp://ftp.ncbi.nlm.nih.gov/genomes/all/GCF/904/419/725/GCF_904419725.1_Chicken_24_mag_188
GCF_001719065.1	TQMD	Fervidobacterium thailandense	100.00	0.53	ftp://ftp.ncbi.nlm.nih.gov/genomes/all/GCF/001/719/065/GCF_001719065.1_ASM171906v1
GCF_002026175.1	TQMD	Prochlorococcus sp.	25.86	0.00	ftp://ftp.ncbi.nlm.nih.gov/genomes/all/GCF/002/026/175/GCF_002026175.1_ASM202617v1
GCF_903819165.1	TQMD	Peptoniphilus sp.	98.95	0.00	ftp://ftp.ncbi.nlm.nih.gov/genomes/all/GCF/903/819/165/GCF_903819165.1_1804121828
GCF_902506685.1	TQMD	Negativicoccus succinicivorans	97.22	0.63	ftp://ftp.ncbi.nlm.nih.gov/genomes/all/GCF/902/506/685/GCF_902506685.1_Q0888
GCF_000340905.1	TQMD	Kinetoplastibacterium galatii	83.97	0.00	ftp://ftp.ncbi.nlm.nih.gov/genomes/all/GCF/000/340/905/GCF_000340905.1_ASM34090v1

GCF_004792615.1	TQMD	Pasteurella multocida	99.55	0.00	ftp://ftp.ncbi.nlm.nih.gov/genomes/all/GCF/004/792/615/GCF_004792615.1_ASM479261v1
GCF_000319575.2	TQMD	Spiribacter salinus	96.31	0.00	ftp://ftp.ncbi.nlm.nih.gov/genomes/all/GCF/000/319/575/GCF_000319575.2_ASM31957v2
GCF_002288125.1	TQMD	Taylorella equigenitalis	100.00	0.00	ftp://ftp.ncbi.nlm.nih.gov/genomes/all/GCF/002/288/125/GCF_002288125.1_ASM228812v1
GCF_002968395.1	TQMD	Idiomarina sp.	99.66	0.17	ftp://ftp.ncbi.nlm.nih.gov/genomes/all/GCF/002/968/395/GCF_002968395.1_ASM296839v1
GCF_004124255.1	TQMD	Pseudomonas adelgestsugas	35.83	0.32	ftp://ftp.ncbi.nlm.nih.gov/genomes/all/GCF/004/124/255/GCF_004124255.1_ASM412425v1
GCF_000219175.1	TQMD	Moranella endobia	88.75	0.00	ftp://ftp.ncbi.nlm.nih.gov/genomes/all/GCF/000/219/175/GCF_000219175.1_ASM21917v1
GCF_900048045.1	TQMD	Mikella endobia	82.50	0.00	ftp://ftp.ncbi.nlm.nih.gov/genomes/all/GCF/900/048/045/GCF_900048045.1_MEPMAR
GCF_000287335.1	TQMD	secondary endosymbiont	100.00	0.00	ftp://ftp.ncbi.nlm.nih.gov/genomes/all/GCF/000/287/335/GCF_000287335.1_ASM28733v1
GCF_003261295.1	TQMD	Polynucleobacter paneuropaeus	93.48	0.20	ftp://ftp.ncbi.nlm.nih.gov/genomes/all/GCF/003/261/295/GCF_003261295.1_ASM326129v1
GCF_000266925.1	TQMD	Acetomicrobium mobile	100.00	0.00	ftp://ftp.ncbi.nlm.nih.gov/genomes/all/GCF/000/266/925/GCF_000266925.1_ASM26692v1
GCF_003194085.1	TQMD	Aurantimicrobium sp.	97.47	0.00	ftp://ftp.ncbi.nlm.nih.gov/genomes/all/GCF/003/194/085/GCF_003194085.1_ASM319408v1
GCF_003351865.1	TQMD	Methanofervidicoccus sp.	99.52	0.00	ftp://ftp.ncbi.nlm.nih.gov/genomes/all/GCF/003/351/865/GCF_003351865.1_ASM335186v1
GCF_009827135.1	TQMD	Blochmannia endosymbiont	100.00	0.00	ftp://ftp.ncbi.nlm.nih.gov/genomes/all/GCF/009/827/135/GCF_009827135.1_ASM982713v1
GCF_016907845.1	TQMD	Arcanobacterium pluranimalium	100.00	0.86	ftp://ftp.ncbi.nlm.nih.gov/genomes/all/GCF/016/907/845/GCF_016907845.1_ASM1690784v1
GCF_005845345.1	TQMD	Rhodoluna limnophila	99.42	0.00	ftp://ftp.ncbi.nlm.nih.gov/genomes/all/GCF/005/845/345/GCF_005845345.1_ASM584534v1
GCF_000212375.1	TQMD	Porphyromonas asaccharolytica	99.92	0.00	ftp://ftp.ncbi.nlm.nih.gov/genomes/all/GCF/000/212/375/GCF_000212375.1_ASM21237v1
GCF_013283855.1	TQMD	Aquiluna borgnonia	98.10	0.00	ftp://ftp.ncbi.nlm.nih.gov/genomes/all/GCF/013/283/855/GCF_013283855.1_ASM1328385v1
GCF_001854225.1	TQMD	Rhodoluna planktonica	99.85	0.00	ftp://ftp.ncbi.nlm.nih.gov/genomes/all/GCF/001/854/225/GCF_001854225.1_ASM185422v1
GCF_000815025.1	TQMD	Coxiella endosymbiont	80.14	0.05	ftp://ftp.ncbi.nlm.nih.gov/genomes/all/GCF/000/815/025/GCF_000815025.1_ASM81502v1
GCF_000025885.1	TQMD	Aminobacterium colombiense	100.00	0.00	ftp://ftp.ncbi.nlm.nih.gov/genomes/all/GCF/000/025/885/GCF_000025885.1_ASM2588v1
GCF_000699505.1	TQMD	Rhodoluna lacicola	99.42	0.00	ftp://ftp.ncbi.nlm.nih.gov/genomes/all/GCF/000/699/505/GCF_000699505.1_ASM69950v1
GCF_009883795.1	TQMD	Coxiella endosymbiont	74.52	0.00	ftp://ftp.ncbi.nlm.nih.gov/genomes/all/GCF/009/883/795/GCF_009883795.1_ASM988379v1
GCF_002288305.1	TQMD	Nanopelagicus abundans	81.50	0.53	ftp://ftp.ncbi.nlm.nih.gov/genomes/all/GCF/002/288/305/GCF_002288305.1_ASM228830v1
GCF_000263735.1	TQMD	Pyrococcus sp.	100.00	0.00	ftp://ftp.ncbi.nlm.nih.gov/genomes/all/GCF/000/263/735/GCF_000263735.1_ASM26373v1
GCF_900475915.1	TQMD	Arcanobacterium haemolyticum	97.99	0.00	ftp://ftp.ncbi.nlm.nih.gov/genomes/all/GCF/900/475/915/GCF_900475915.1_48128_H02
GCF_002288365.1	TQMD	Planktophila limnetica	88.70	0.53	ftp://ftp.ncbi.nlm.nih.gov/genomes/all/GCF/002/288/365/GCF_002288365.1_ASM228836v1
GCF_002287885.2	TQMD	Nanopelagicus limnes	80.01	0.00	ftp://ftp.ncbi.nlm.nih.gov/genomes/all/GCF/002/287/885/GCF_002287885.2_ASM228788v2
GCF_016904675.1	TQMD	Arcanobacterium phocisimile	98.85	0.86	ftp://ftp.ncbi.nlm.nih.gov/genomes/all/GCF/016/904/675/GCF_016904675.1_ASM1690467v1
GCF_900698955.1	TQMD	Erwinia haradaeae	41.15	0.00	ftp://ftp.ncbi.nlm.nih.gov/genomes/all/GCF/900/698/955/GCF_900698955.1_ErCilaricifoliae_v1.0

GCF_000265545.3	TQMD	Corynebacterium pseudotuberculosis	99.56	0.00	ftp://ftp.ncbi.nlm.nih.gov/genomes/all/GCF/000/265/545/GCF_000265545.3_ASM26554v3
GCF_002288065.1	TQMD	Planktophila sulfonica	90.54	0.70	ftp://ftp.ncbi.nlm.nih.gov/genomes/all/GCF/002/288/065/GCF_002288065.1_ASM228806v1
GCF_002288185.1	TQMD	Planktophila vernalis	87.82	0.26	ftp://ftp.ncbi.nlm.nih.gov/genomes/all/GCF/002/288/185/GCF_002288185.1_ASM228818v1
GCF_900039485.1	TQMD	Doolittlea endobia	97.53	0.00	ftp://ftp.ncbi.nlm.nih.gov/genomes/all/GCF/900/039/485/GCF_900039485.1_DEMHIR
GCF_002849975.1	TQMD	Portiera aleyrodidarum	19.32	0.00	ftp://ftp.ncbi.nlm.nih.gov/genomes/all/GCF/002/849/975/GCF_002849975.1_ASM284997v1
GCF_014879315.1	TQMD	Riesia pediculicola	93.33	1.25	ftp://ftp.ncbi.nlm.nih.gov/genomes/all/GCF/014/879/315/GCF_014879315.1_ASM1487931v1
GCF_000981505.1	TQMD	Methylopumilus planktonicus	98.27	0.00	ftp://ftp.ncbi.nlm.nih.gov/genomes/all/GCF/000/981/505/GCF_000981505.1_Candidatus_Methylopumilus_planktonicus_MMS-2-53
GCF_000208385.1	TQMD	Sphaerochaeta coccoides	100.00	2.30	ftp://ftp.ncbi.nlm.nih.gov/genomes/all/GCF/000/208/385/GCF_000208385.1_ASM20838v1
GCF_000018365.1	TQMD	Thermococcus onnurineus	97.52	0.00	ftp://ftp.ncbi.nlm.nih.gov/genomes/all/GCF/000/018/365/GCF_000018365.1_ASM1836v1
GCF_002285875.1	TQMD	Portiera aleyrodidarum	18.28	0.00	ftp://ftp.ncbi.nlm.nih.gov/genomes/all/GCF/002/285/875/GCF_002285875.1_ASM228587v1
GCF_000233775.1	TQMD	Thermovirga lienii	100.00	0.00	ftp://ftp.ncbi.nlm.nih.gov/genomes/all/GCF/000/233/775/GCF_000233775.1_ASM23377v1
GCF_007904105.1	TQMD	Corynebacterium sp.	98.31	0.22	ftp://ftp.ncbi.nlm.nih.gov/genomes/all/GCF/007/904/105/GCF_007904105.1_ASM790410v1
GCF_001042695.1	TQMD	Scardovia inopinata	99.30	0.47	ftp://ftp.ncbi.nlm.nih.gov/genomes/all/GCF/001/042/695/GCF_001042695.1_ASM104269v1
GCF_016026575.1	TQMD	Fannyhessea vaginae	100.00	0.00	ftp://ftp.ncbi.nlm.nih.gov/genomes/all/GCF/016/026/575/GCF_016026575.1_ASM1602657v1
GCF_000591035.1	TQMD	Aeropyrum camini	97.15	0.63	ftp://ftp.ncbi.nlm.nih.gov/genomes/all/GCF/000/591/035/GCF_000591035.1_ASM59103v1
GCF_000186365.1	TQMD	Desulfurococcus mucosus	100.00	0.63	ftp://ftp.ncbi.nlm.nih.gov/genomes/all/GCF/000/186/365/GCF_000186365.1_ASM18636v1
GCF_000264495.1	TQMD	Thermogladius calderae	100.00	0.00	ftp://ftp.ncbi.nlm.nih.gov/genomes/all/GCF/000/264/495/GCF_000264495.1_ASM26449v1
GCF_000709555.1	TQMD	Walczuchella monophlebidarum	67.38	0.00	ftp://ftp.ncbi.nlm.nih.gov/genomes/all/GCF/000/709/555/GCF_000709555.1_ASM70955v1
GCF_000144915.1	TQMD	Acidilobus saccharovorans	99.37	0.00	ftp://ftp.ncbi.nlm.nih.gov/genomes/all/GCF/000/144/915/GCF_000144915.1_ASM14491v1
GCF_000019745.1	TQMD	Polynucleobacter necessarius	92.78	0.21	ftp://ftp.ncbi.nlm.nih.gov/genomes/all/GCF/000/019/745/GCF_000019745.1_ASM1974v1
GCF_000231015.2	TQMD	Desulfurococcus amylolyticus	100.00	0.00	ftp://ftp.ncbi.nlm.nih.gov/genomes/all/GCF/000/231/015/GCF_000231015.2_ASM23101v3
GCF_000269605.1	TQMD	Scardovia wiggsiae	95.99	0.15	ftp://ftp.ncbi.nlm.nih.gov/genomes/all/GCF/000/269/605/GCF_000269605.1_PB_Scar_wigg_F0424_V1
GCF_000196135.1	TQMD	Wolinella succinogenes	99.41	0.42	ftp://ftp.ncbi.nlm.nih.gov/genomes/all/GCF/000/196/135/GCF_000196135.1_ASM19613v1
GCF_014962245.1	TQMD	Thermosphaera aggregans	99.37	0.00	ftp://ftp.ncbi.nlm.nih.gov/genomes/all/GCF/014/962/245/GCF_014962245.1_ASM1496224v1
GCF_000217655.1	TQMD	Treponema paraluiscuniculi	99.19	0.00	ftp://ftp.ncbi.nlm.nih.gov/genomes/all/GCF/000/217/655/GCF_000217655.1_ASM21765v1
GCF_003397585.1	TQMD	Gardnerella piotii	100.00	0.00	ftp://ftp.ncbi.nlm.nih.gov/genomes/all/GCF/003/397/585/GCF_003397585.1_ASM339758v1
GCF_000015145.1	TQMD	Hyperthermus butylicus	98.73	1.42	ftp://ftp.ncbi.nlm.nih.gov/genomes/all/GCF/000/015/145/GCF_000015145.1_ASM1514v1
GCF_000446015.1	TQMD	Thermofilum adornatum	99.26	0.74	ftp://ftp.ncbi.nlm.nih.gov/genomes/all/GCF/000/446/015/GCF_000446015.1_ASM44601v1
GCF_000020945.1	TQMD	Coprothermobacter proteolyticus	100.00	0.00	ftp://ftp.ncbi.nlm.nih.gov/genomes/all/GCF/000/020/945/GCF_000020945.1_ASM2094v1
GCF_016888765.1	TQMD	Sulcia muelleri	43.32	0.00	ftp://ftp.ncbi.nlm.nih.gov/genomes/all/GCF/016/888/765/GCF_016888765.1_ASM1688876v1

GCF_000092185.1	TQMD	Thermosphaera aggregans	98.73	0.00	ftp://ftp.ncbi.nlm.nih.gov/genomes/all/GCF/000/092/185/GCF_000092185.1_ASM9218v1
GCF_000008645.1	TQMD	Methanothermobacter thermautotrophicus	100.00	0.31	ftp://ftp.ncbi.nlm.nih.gov/genomes/all/GCF/000/008/645/GCF_000008645.1_ASM864v1
GCF_001856685.1	TQMD	Boudabousia tangfeifanii	98.46	1.66	ftp://ftp.ncbi.nlm.nih.gov/genomes/all/GCF/001/856/685/GCF_001856685.1_ASM185668v1
GCF_000993805.1	TQMD	Thermofilum uzonense	98.53	0.74	ftp://ftp.ncbi.nlm.nih.gov/genomes/all/GCF/000/993/805/GCF_000993805.1_ASM99380v1
GCF_000196075.1	TQMD	Tropheryma whipplei	76.27	0.00	ftp://ftp.ncbi.nlm.nih.gov/genomes/all/GCF/000/196/075/GCF_000196075.1_ASM19607v1
GCF_000223395.1	TQMD	Pyrolobus fumarii	99.05	0.79	ftp://ftp.ncbi.nlm.nih.gov/genomes/all/GCF/000/223/395/GCF_000223395.1_ASM22339v1
GCF_000017945.1	TQMD	Ignicoccus hospitalis	99.37	0.84	ftp://ftp.ncbi.nlm.nih.gov/genomes/all/GCF/000/017/945/GCF_000017945.1_ASM1794v1
GCF_000015805.1	TQMD	Pyrobaculum calidifontis	100.00	0.00	ftp://ftp.ncbi.nlm.nih.gov/genomes/all/GCF/000/015/805/GCF_000015805.1_ASM1580v1
GCF_000014945.1	TQMD	Methanothrix thermoacetophila	100.00	0.00	ftp://ftp.ncbi.nlm.nih.gov/genomes/all/GCF/000/014/945/GCF_000014945.1_ASM1494v1
GCF_000068525.2	TQMD	Chlamydia trachomatis	98.92	0.00	ftp://ftp.ncbi.nlm.nih.gov/genomes/all/GCF/000/068/525/GCF_000068525.2_ASM6852v2
GCF_013408435.1	TQMD	Aeriscardovia aeriphila	94.58	0.00	ftp://ftp.ncbi.nlm.nih.gov/genomes/all/GCF/013/408/435/GCF_013408435.1_ASM1340843v1
GCF_000019605.1	TQMD	Korarchaeum cryptofilum	93.39	2.80	ftp://ftp.ncbi.nlm.nih.gov/genomes/all/GCF/000/019/605/GCF_000019605.1_ASM1960v1
GCF_000190315.1	TQMD	Vulcanisaeta moutnovskia	100.00	0.74	ftp://ftp.ncbi.nlm.nih.gov/genomes/all/GCF/000/190/315/GCF_000190315.1_ASM19031v1
GCF_014876775.1	TQMD	Infirmifilum lucidum	98.53	0.74	ftp://ftp.ncbi.nlm.nih.gov/genomes/all/GCF/014/876/775/GCF_014876775.1_ASM1487677v1
GCF_003351905.1	TQMD	Cardinium endosymbiont	73.11	0.55	ftp://ftp.ncbi.nlm.nih.gov/genomes/all/GCF/003/351/905/GCF_003351905.1_ASM335190v1
GCF_000013165.1	TQMD	Neorickettsia sennetsu	99.28	0.00	ftp://ftp.ncbi.nlm.nih.gov/genomes/all/GCF/000/013/165/GCF_000013165.1_ASM1316v1
GCF_009861485.1	TQMD	Massilia sp.	11.20	0.00	ftp://ftp.ncbi.nlm.nih.gov/genomes/all/GCF/009/861/485/GCF_009861485.1_ASM986148v1
GCF_002214625.1	TQMD	Anaplasma ovis	99.36	1.21	ftp://ftp.ncbi.nlm.nih.gov/genomes/all/GCF/002/214/625/GCF_002214625.1_ASM221462v2
GCF_001006045.1	TQMD	Geoglobus ahangari	100.00	0.00	ftp://ftp.ncbi.nlm.nih.gov/genomes/all/GCF/001/006/045/GCF_001006045.1_ASM100604v1
GCF_900002505.1	TQMD	Chlamydia abortus	98.98	0.00	ftp://ftp.ncbi.nlm.nih.gov/genomes/all/GCF/900/002/505/GCF_900002505.1_CA1H
GCF_000024505.1	TQMD	Anaplasma centrale	100.00	0.64	ftp://ftp.ncbi.nlm.nih.gov/genomes/all/GCF/000/024/505/GCF_000024505.1_ASM2450v1
GCF_003004665.1	TQMD	Pandoraea novymonadis	76.52	0.06	ftp://ftp.ncbi.nlm.nih.gov/genomes/all/GCF/003/004/665/GCF_003004665.1_ASM300466v1
GCF_000194625.1	TQMD	Archaeoglobus veneficus	99.35	0.00	ftp://ftp.ncbi.nlm.nih.gov/genomes/all/GCF/000/194/625/GCF_000194625.1_ASM19462v1
GCF_900187875.1	TQMD	Polynucleobacter victoriensis	95.75	0.00	ftp://ftp.ncbi.nlm.nih.gov/genomes/all/GCF/900/187/875/GCF_900187875.1_IMG-taxon_2710264786_annotated_assembly
GCF_000204135.1	TQMD	Chlamydia pecorum	98.98	0.00	ftp://ftp.ncbi.nlm.nih.gov/genomes/all/GCF/000/204/135/GCF_000204135.1_ASM20413v1
GCF_000015765.1	TQMD	Methanocorpusculum labreanum	99.54	0.00	ftp://ftp.ncbi.nlm.nih.gov/genomes/all/GCF/000/015/765/GCF_000015765.1_ASM1576v1
GCF_000008745.1	TQMD	Chlamydia pneumoniae	99.49	0.00	ftp://ftp.ncbi.nlm.nih.gov/genomes/all/GCF/000/008/745/GCF_000008745.1_ASM874v1
GCF_000632985.1	TQMD	Neorickettsia helminthoeca	97.47	0.47	ftp://ftp.ncbi.nlm.nih.gov/genomes/all/GCF/000/632/985/GCF_000632985.1_ASM63298v1
GCF_003176915.1	TQMD	Cardinium hertigii	70.11	1.09	ftp://ftp.ncbi.nlm.nih.gov/genomes/all/GCF/003/176/915/GCF_003176915.1_ASM317691v1
GCF_000829315.1	TQMD	Wolbachia endosymbiont	100.00	0.43	ftp://ftp.ncbi.nlm.nih.gov/genomes/all/GCF/000/829/315/GCF_000829315.1_ASM82931v1
GCF_900044015.1	TQMD	Hoaglandella endobia	97.53	0.00	ftp://ftp.ncbi.nlm.nih.gov/genomes/all/GCF/900/044/015/GCF_900044015.1_HETPER1

GCF_000013125.1	TQMD	Anaplasma phagocytophilum	99.57	3.50	ftp://ftp.ncbi.nlm.nih.gov/genomes/all/GCF/000/013/125/GCF_000013125.1_ASM1312v1
GCF_003967175.1	TQMD	Sulfodiicoccus acidiphilus	97.62	0.60	ftp://ftp.ncbi.nlm.nih.gov/genomes/all/GCF/003/967/175/GCF_003967175.1_Sacidi_1.0
GCF_900105865.1	TQMD	Arcanobacterium phocae	98.85	3.02	ftp://ftp.ncbi.nlm.nih.gov/genomes/all/GCF/900/105/865/GCF_900105865.1_IMG-taxon_2634166335_annotated_assembly
GCF_900100865.1	TQMD	Aquiluna sp.	98.10	0.00	ftp://ftp.ncbi.nlm.nih.gov/genomes/all/GCF/900/100/865/GCF_900100865.1_IMG-taxon_2675903689_annotated_assembly
GCF_900416725.1	TQMD	Chlamydia abortus	98.98	0.00	ftp://ftp.ncbi.nlm.nih.gov/genomes/all/GCF/900/416/725/GCF_900416725.1_668
GCF_000024145.1	TQMD	Chlamydia pneumoniae	99.49	0.00	ftp://ftp.ncbi.nlm.nih.gov/genomes/all/GCF/000/024/145/GCF_000024145.1_ASM2414v1
GCF_001398155.1	TQMD	Chlamydia trachomatis	98.92	0.00	ftp://ftp.ncbi.nlm.nih.gov/genomes/all/GCF/001/398/155/GCF_001398155.1_7501_6_49
GCF_900155645.1	TQMD	Propionimicrobium sp.	98.68	0.66	ftp://ftp.ncbi.nlm.nih.gov/genomes/all/GCF/900/155/645/GCF_900155645.1_Propionibacterium_massiliense
GCF_000441575.1	TQMD	Carsonella ruddii	20.32	0.00	ftp://ftp.ncbi.nlm.nih.gov/genomes/all/GCF/000/441/575/GCF_000441575.1_ASM44157v1
GCF_011752015.1	TQMD	Canibacter sp.	95.13	0.00	ftp://ftp.ncbi.nlm.nih.gov/genomes/all/GCF/011/752/015/GCF_011752015.1_ASM1175201v1
GCF_000025285.1	TQMD	Archaeoglobus profundus	99.84	0.00	ftp://ftp.ncbi.nlm.nih.gov/genomes/all/GCF/000/025/285/GCF_000025285.1_ASM2528v1
GCF_000955905.1	TQMD	Nitrosotenuis cloacae	100.00	1.94	ftp://ftp.ncbi.nlm.nih.gov/genomes/all/GCF/000/955/905/GCF_000955905.1_ASM95590v3
GCF_011752005.1	TQMD	Canibacter sp.	95.13	0.00	ftp://ftp.ncbi.nlm.nih.gov/genomes/all/GCF/011/752/005/GCF_011752005.1_ASM1175200v1
GCF_000146025.2	TQMD	Endomicrobium trichonymphae	95.51	0.00	ftp://ftp.ncbi.nlm.nih.gov/genomes/all/GCF/000/146/025/GCF_000146025.2_ASM14602v1
GCF_002797575.1	TQMD	Polynucleobacter sp.	98.96	0.52	ftp://ftp.ncbi.nlm.nih.gov/genomes/all/GCF/002/797/575/GCF_002797575.1_ASM279757v1
GCF_003858425.1	TQMD	Leucobacter sp.	94.83	0.00	ftp://ftp.ncbi.nlm.nih.gov/genomes/all/GCF/003/858/425/GCF_003858425.1_ASM385842v1
GCF_001481685.1	TQMD	Ignicoccus islandicus	99.37	1.48	ftp://ftp.ncbi.nlm.nih.gov/genomes/all/GCF/001/481/685/GCF_001481685.1_ASM148168v1
GCF_000376885.1	TQMD	Alloscardovia criceti	99.77	0.00	ftp://ftp.ncbi.nlm.nih.gov/genomes/all/GCF/000/376/885/GCF_000376885.1_ASM37688v1
GCF_001318295.1	TQMD	Xiphinematobacter sp.	89.86	0.00	ftp://ftp.ncbi.nlm.nih.gov/genomes/all/GCF/001/318/295/GCF_001318295.1_ASM131829v1
GCF_003264935.1	TQMD	Methanothermobacter tenebrarum	99.20	0.00	ftp://ftp.ncbi.nlm.nih.gov/genomes/all/GCF/003/264/935/GCF_003264935.1_ASM326493v1
GCF_009829915.1	TQMD	Pantoea sp.	59.36	0.00	ftp://ftp.ncbi.nlm.nih.gov/genomes/all/GCF/009/829/915/GCF_009829915.1_ASM982991v1
GCF_000257665.1	TQMD	Aquiluna sp.	97.51	0.00	ftp://ftp.ncbi.nlm.nih.gov/genomes/all/GCF/000/257/665/GCF_000257665.1_ASM25766v1
GCF_009696505.1	TQMD	Bifidobacterium tsurumiense	99.09	0.11	ftp://ftp.ncbi.nlm.nih.gov/genomes/all/GCF/009/696/505/GCF_009696505.1_ASM969650v1
GCF_001053655.1	TQMD	Alloscardovia omnicolens	99.88	0.00	ftp://ftp.ncbi.nlm.nih.gov/genomes/all/GCF/001/053/655/GCF_001053655.1_ASM105365v1
GCF_009725755.1	TQMD	Bombella sp.	99.30	0.25	ftp://ftp.ncbi.nlm.nih.gov/genomes/all/GCF/009/725/755/GCF_009725755.1_ASM972575v1
GCF_000420065.1	TQMD	Varibaculum cambriense	99.53	0.95	ftp://ftp.ncbi.nlm.nih.gov/genomes/all/GCF/000/420/065/GCF_000420065.1_ASM42006v1
GCF_000025605.1	TQMD	Thermocrinis albus	99.39	0.00	ftp://ftp.ncbi.nlm.nih.gov/genomes/all/GCF/000/025/605/GCF_000025605.1_ASM2560v1
GCF_003951975.1	TQMD	Bifidobacterium dolichotidis	97.56	0.67	ftp://ftp.ncbi.nlm.nih.gov/genomes/all/GCF/003/951/975/GCF_003951975.1_ASM395197v1
GCF_900205755.1	TQMD	Polynucleobacter meluiroseus	99.12	0.00	ftp://ftp.ncbi.nlm.nih.gov/genomes/all/GCF/900/205/755/GCF_900205755.1_IMG-taxon_2710724120_annotated_assembly
GCF_000327505.1	TQMD	Aciduliprofundum sp.	100.00	0.00	ftp://ftp.ncbi.nlm.nih.gov/genomes/all/GCF/000/327/505/GCF_000327505.1_ASM32750v1
GCF_003664005.1	TQMD	Hydrogenivirga caldilitoris	99.59	0.00	ftp://ftp.ncbi.nlm.nih.gov/genomes/all/GCF/003/664/005/GCF_003664005.1_ASM366400v1
GCF_000800805.1	TQMD	Methanoplasma termitum	97.85	1.61	ftp://ftp.ncbi.nlm.nih.gov/genomes/all/GCF/000/800/805/GCF_000800805.1_ASM80080v1

GCF_001907275.1	TQMD	Boudabousia liubingyangii	97.27	1.18	ftp://ftp.ncbi.nlm.nih.gov/genomes/all/GCF/001/907/275/GCF_001907275.1_ASM190727v1
GCF_000195915.1	TQMD	Thermoplasma acidophilum	97.52	0.00	ftp://ftp.ncbi.nlm.nih.gov/genomes/all/GCF/000/195/915/GCF_000195915.1_ASM19591v1
GCF_000304455.1	TQMD	Cardinium endosymbiont	72.93	0.55	ftp://ftp.ncbi.nlm.nih.gov/genomes/all/GCF/000/304/455/GCF_000304455.1_CCh_cEper1
GCF_000011185.1	TQMD	Thermoplasma volcanium	97.97	0.00	ftp://ftp.ncbi.nlm.nih.gov/genomes/all/GCF/000/011/185/GCF_000011185.1_ASM1118v1
GCF_001653975.1	TQMD	Chlamydia sp.	98.98	0.00	ftp://ftp.ncbi.nlm.nih.gov/genomes/all/GCF/001/653/975/GCF_001653975.1_ASM165397v1
GCF_000319205.1	TQMD	Helicobacter heilmannii	94.56	4.84	ftp://ftp.ncbi.nlm.nih.gov/genomes/all/GCF/000/319/205/GCF_000319205.1_ASM31920v1
GCF_012790675.1	TQMD	Anaplasma platys	98.15	1.53	ftp://ftp.ncbi.nlm.nih.gov/genomes/all/GCF/012/790/675/GCF_012790675.1_ASM1279067v1
GCF_000803625.1	TQMD	Nanosynbacter lyticus	65.38	0.00	ftp://ftp.ncbi.nlm.nih.gov/genomes/all/GCF/000/803/625/GCF_000803625.1_ASM80362v1
GCF_900604515.1	TQMD	Arcanobacterium ihumii	99.71	0.86	ftp://ftp.ncbi.nlm.nih.gov/genomes/all/GCF/900/604/515/GCF_900604515.1_PRJEB25658
GCF_003072485.1	TQMD	Fokinia solitaria	89.38	0.00	ftp://ftp.ncbi.nlm.nih.gov/genomes/all/GCF/003/072/485/GCF_003072485.1_ASM307248v1
GCF_000454725.1	TQMD	Chlamydia ibidis	99.49	0.00	ftp://ftp.ncbi.nlm.nih.gov/genomes/all/GCF/000/454/725/GCF_000454725.1_ibidis.assembly
GCF_002906215.1	TQMD	Nitrosocaldus islandicus	99.03	0.00	ftp://ftp.ncbi.nlm.nih.gov/genomes/all/GCF/002/906/215/GCF_002906215.1_ASM290621v1
GCF_000392435.1	TQMD	Saccharimonas aalborgensis	67.13	0.93	ftp://ftp.ncbi.nlm.nih.gov/genomes/all/GCF/000/392/435/GCF_000392435.1_ASM39243v1
GCF_003391295.1	TQMD	Sulcia muelleri	41.98	0.00	ftp://ftp.ncbi.nlm.nih.gov/genomes/all/GCF/003/391/295/GCF_003391295.1_ASM339129v1
GCF_000185805.1	TQMD	Thermovibrio ammonificans	99.58	0.84	ftp://ftp.ncbi.nlm.nih.gov/genomes/all/GCF/000/185/805/GCF_000185805.1_ASM18580v1
GCF_002159705.1	TQMD	Elusimicrobium sp.	96.63	0.00	ftp://ftp.ncbi.nlm.nih.gov/genomes/all/GCF/002/159/705/GCF_002159705.1_ASM215970v1
GCF_009696615.1	TQMD	Scrofimicrobium canadense	99.53	1.66	ftp://ftp.ncbi.nlm.nih.gov/genomes/all/GCF/009/696/615/GCF_009696615.1_ASM969661v1
GCF_902705475.1	TQMD	Chlamydia sp.	88.13	0.00	ftp://ftp.ncbi.nlm.nih.gov/genomes/all/GCF/902/705/475/GCF_902705475.1_17-3921_L77
GCF_009936135.1	TQMD	Chazhemtobacterium aquaticus	64.76	0.00	ftp://ftp.ncbi.nlm.nih.gov/genomes/all/GCF/009/936/135/GCF_009936135.1_ASM993613v1
GCF_000711215.1	TQMD	Methanomicrobium mobile	97.39	0.65	ftp://ftp.ncbi.nlm.nih.gov/genomes/all/GCF/000/711/215/GCF_000711215.1_ASM71121v1
GCF_900089565.1	TQMD	Hydrotalea flava	34.73	0.00	ftp://ftp.ncbi.nlm.nih.gov/genomes/all/GCF/900/089/565/GCF_900089565.1_K06
GCF_014635685.1	TQMD	Galliscardovia ingluviei	98.64	1.06	ftp://ftp.ncbi.nlm.nih.gov/genomes/all/GCF/014/635/685/GCF_014635685.1_ASM1463568v1
GCF_002073895.1	TQMD	Riesia pediculischaeffi	91.46	0.00	ftp://ftp.ncbi.nlm.nih.gov/genomes/all/GCF/002/073/895/GCF_002073895.1_ASM207389v1
GCF_000758825.1	TQMD	Arcanobacterium sp.	96.55	0.00	ftp://ftp.ncbi.nlm.nih.gov/genomes/all/GCF/000/758/825/GCF_000758825.1_04_NF40_HMP1631v01
GCF_016806735.1	TQMD	Micrarchaeum sp.	81.78	0.93	ftp://ftp.ncbi.nlm.nih.gov/genomes/all/GCF/016/806/735/GCF_016806735.1_ASM1680673v1
GCF_002214165.1	TQMD	Mancarchaeum acidiphilum	82.40	0.00	ftp://ftp.ncbi.nlm.nih.gov/genomes/all/GCF/002/214/165/GCF_002214165.1_ASM221416v1
GCF_002259585.1	TQMD	Bombiscardovia coagulans	92.43	1.16	ftp://ftp.ncbi.nlm.nih.gov/genomes/all/GCF/002/259/585/GCF_002259585.1_ASM225958v1
GCF_000565015.1	TQMD	Porphyromonas catoniae	95.05	0.00	ftp://ftp.ncbi.nlm.nih.gov/genomes/all/GCF/000/565/015/GCF_000565015.1_PcatATCC51270v1.0
GCF_000483125.1	TQMD	Atopobium fossor	100.00	0.00	ftp://ftp.ncbi.nlm.nih.gov/genomes/all/GCF/000/483/125/GCF_000483125.1_ASM48312v1
GCF_001687305.1	TQMD	Actinomyces vulturis	98.82	1.18	ftp://ftp.ncbi.nlm.nih.gov/genomes/all/GCF/001/687/305/GCF_001687305.1_ASM168730v1
GCF_002904345.1	TQMD	Bacteroidetes	94.80	0.00	ftp://ftp.ncbi.nlm.nih.gov/genomes/all/GCF/002/904/345/GCF_002904345.1_ASM290434v1

		endosymbion t			
GCF_016 901835.1	TQMD	Parasutterell a secunda	97.40	0.62	ftp://ftp.ncbi.nlm.nih.gov/genomes/all/GCF/016/901/835/GCF_016901 835.1_ASM1690183v1
GCF_004 332295.1	TQMD	Alloscardovi a theropitheci	99.32	0.53	ftp://ftp.ncbi.nlm.nih.gov/genomes/all/GCF/004/332/295/GCF_004332 295.1_ASM433229v1
GCF_016 125955.1	TQMD	Anaerobacul um sp.	100.00	0.49	ftp://ftp.ncbi.nlm.nih.gov/genomes/all/GCF/016/125/955/GCF_016125 955.1_ASM1612595v1
GCF_001 028545.1	TQMD	Treponema endosymbion t	55.76	3.62	ftp://ftp.ncbi.nlm.nih.gov/genomes/all/GCF/001/028/545/GCF_001028 545.1_ASM102854v1
GCF_000 379705.1	TQMD	Corynebacter ium caspium	98.13	0.00	ftp://ftp.ncbi.nlm.nih.gov/genomes/all/GCF/000/379/705/GCF_000379 705.1_ASM37970v1
GCF_001 936115.1	TQMD	Boudabousia marimammal ium	98.01	0.47	ftp://ftp.ncbi.nlm.nih.gov/genomes/all/GCF/001/936/115/GCF_001936 115.1_ASM193611v1
GCF_900 188135.1	TQMD	Rhodoplanes sp.	10.34	0.00	ftp://ftp.ncbi.nlm.nih.gov/genomes/all/GCF/900/188/135/GCF_900188 135.1_IMG-taxon_2675903048_annotated_assembly
GCF_001 039595.1	TQMD	Bizionia psychrotoler ans	20.69	0.00	ftp://ftp.ncbi.nlm.nih.gov/genomes/all/GCF/001/039/595/GCF_001039 595.1_ASM103959v1
GCF_002 872015.1	TQMD	Actinotignu m urinale	99.78	0.00	ftp://ftp.ncbi.nlm.nih.gov/genomes/all/GCF/002/872/015/GCF_002872 015.1_ASM287201v1
GCF_000 378005.1	TQMD	Coprothermo bacter platensis	98.21	0.00	ftp://ftp.ncbi.nlm.nih.gov/genomes/all/GCF/000/378/005/GCF_000378 005.1_ASM37800v1
GCF_001 431085.1	TQMD	Symbiothrix dinenymphae	26.06	1.11	ftp://ftp.ncbi.nlm.nih.gov/genomes/all/GCF/001/431/085/GCF_001431 085.1_ASM143108v1
GCF_001 931505.1	TQMD	Pajaroelloba cter abortibovis	81.08	0.00	ftp://ftp.ncbi.nlm.nih.gov/genomes/all/GCF/001/931/505/GCF_001931 505.1_ASM193150v1
GCF_000 159015.1	TQMD	Gleimia coleocanis	99.01	0.87	ftp://ftp.ncbi.nlm.nih.gov/genomes/all/GCF/000/159/015/GCF_000159 015.1_ASM15901v1
GCF_001 552785.1	TQMD	Atopobium deltae	100.00	0.00	ftp://ftp.ncbi.nlm.nih.gov/genomes/all/GCF/001/552/785/GCF_001552 785.1_ASM155278v1
GCF_009 805595.2	TQMD	Tremblaya phenacola	42.34	1.68	ftp://ftp.ncbi.nlm.nih.gov/genomes/all/GCF/009/805/595/GCF_009805 595.2_ASM980559v2
GCF_001 881485.1	TQMD	Rickettsiella grylli	89.49	0.52	ftp://ftp.ncbi.nlm.nih.gov/genomes/all/GCF/001/881/485/GCF_001881 485.1_ASM188148v1
GCF_011 319365.1	TQMD	Rhizobium flavescens	0.00	0.00	ftp://ftp.ncbi.nlm.nih.gov/genomes/all/GCF/011/319/365/GCF_011319 365.1_ASM1131936v1
GCF_008 189685.1	TQMD	Sneabacter namystus	74.64	0.49	ftp://ftp.ncbi.nlm.nih.gov/genomes/all/GCF/008/189/685/GCF_008189 685.1_ASM818968v1
GCF_004 210305.1	TQMD	Finniella inopinata	95.70	0.00	ftp://ftp.ncbi.nlm.nih.gov/genomes/all/GCF/004/210/305/GCF_004210 305.1_ASM421030v1
GCF_004 210275.1	TQMD	Rickettsiales endosymbion t	96.70	0.00	ftp://ftp.ncbi.nlm.nih.gov/genomes/all/GCF/004/210/275/GCF_004210 275.1_ASM421027v1
GCF_900 608495.1	TQMD	Olavius algarvensis	96.00	3.20	ftp://ftp.ncbi.nlm.nih.gov/genomes/all/GCF/900/608/495/GCF_900608 495.1_OalgB6SA-Spiro
GCF_000 417715.1	TQMD	Chlamydia psittaci	97.69	0.00	ftp://ftp.ncbi.nlm.nih.gov/genomes/all/GCF/000/417/715/GCF_000417 715.1_ASM41771v1
GCF_000 711905.1	TQMD	Methermicoc cus shengliensis	100.00	0.00	ftp://ftp.ncbi.nlm.nih.gov/genomes/all/GCF/000/711/905/GCF_000711 905.1_ASM71190v1
GCF_003 788695.1	TQMD	Cardinium hertigii	68.06	9.84	ftp://ftp.ncbi.nlm.nih.gov/genomes/all/GCF/003/788/695/GCF_003788 695.1_ASM378869v1
GCF_004 151455.1	TQMD	Nanogingiva lis gingivitcus	65.31	0.00	ftp://ftp.ncbi.nlm.nih.gov/genomes/all/GCF/004/151/455/GCF_004151 455.1_ASM415145v1
GCF_000 262525.1	TQMD	Leptothrix ochracea	23.28	0.00	ftp://ftp.ncbi.nlm.nih.gov/genomes/all/GCF/000/262/525/GCF_000262 525.1_ASM26252v1
GCF_004 138395.1	TQMD	Nanosyncocc us alces	63.17	0.00	ftp://ftp.ncbi.nlm.nih.gov/genomes/all/GCF/004/138/395/GCF_004138 395.1_ASM413839v1
GCF_016 751895.1	TQMD	Sarmatiella mevalonica	86.10	0.47	ftp://ftp.ncbi.nlm.nih.gov/genomes/all/GCF/016/751/895/GCF_016751 895.1_ASM1675189v1
GCF_000 702425.1	TQMD	Thermocrinis sp.	98.78	0.00	ftp://ftp.ncbi.nlm.nih.gov/genomes/all/GCF/000/702/425/GCF_000702 425.1_ASM70242v1
GCF_003 339615.1	TQMD	Similichlamy dia laticola	62.19	0.00	ftp://ftp.ncbi.nlm.nih.gov/genomes/all/GCF/003/339/615/GCF_003339 615.1_ASM333961v1
GCF_002 153915.1	TQMD	Methanonatr onarchaeum	97.22	2.61	ftp://ftp.ncbi.nlm.nih.gov/genomes/all/GCF/002/153/915/GCF_002153 915.1_ASM215391v1

		thermophilum			
GCF_014646795.1	TQMD	Thermogymnomonas acidicola	98.75	0.81	ftp://ftp.ncbi.nlm.nih.gov/genomes/all/GCF/014/646/795/GCF_014646795.1_ASM1464679v1
GCF_002803295.2	TQMD	Caedibacter taeniospiralis	68.44	0.19	ftp://ftp.ncbi.nlm.nih.gov/genomes/all/GCF/002/803/295/GCF_002803295.2_ASM280329v2
GCF_000742475.1	TQMD	Hepatobacter penaei	91.94	0.00	ftp://ftp.ncbi.nlm.nih.gov/genomes/all/GCF/000/742/475/GCF_000742475.1_ASM74247v1
GCF_013282035.1	TQMD	Hakubanella thermoalkaliphilus	54.78	1.82	ftp://ftp.ncbi.nlm.nih.gov/genomes/all/GCF/013/282/035/GCF_013282035.1_ASM1328203v1
GCF_004138445.1	TQMD	Nanoperiomorbus periodonticus	56.55	1.71	ftp://ftp.ncbi.nlm.nih.gov/genomes/all/GCF/004/138/445/GCF_004138445.1_ASM413844v1
GCF_004138405.1	TQMD	Nanosyncoccus nanoralicus	74.84	1.72	ftp://ftp.ncbi.nlm.nih.gov/genomes/all/GCF/004/138/405/GCF_004138405.1_ASM413840v1
GCF_004138385.1	TQMD	Nanosynsaccharı sp.	58.81	1.95	ftp://ftp.ncbi.nlm.nih.gov/genomes/all/GCF/004/138/385/GCF_004138385.1_ASM413838v1
